# Sharp-SSL: Selective High-Dimensional Axis-Aligned Random Projections for Semi-Supervised Learning

**DOI:** 10.1080/01621459.2024.2340792

**Published:** 2024-04-12

**Authors:** Tengyao Wang, Edgar Dobriban, Milana Gataric, Richard J. Samworth

**Affiliations:** a Department of Statistics, London School of Economics, London, UK; b Department of Statistics and Data Science, University of Pennsylvania, Philadelphia, PA; c Statistical Laboratory, University of Cambridge, Cambridge, UK

**Keywords:** Ensemble learning, High-dimensional statistics, Random projection, Semi-supervised learning, Sparsity

## Abstract

We propose a new method for high-dimensional semi-supervised learning problems based on the careful aggregation of the results of a low-dimensional procedure applied to many axis-aligned random projections of the data. Our primary goal is to identify important variables for distinguishing between the classes; existing low-dimensional methods can then be applied for final class assignment. To this end, we score projections according to their class-distinguishing ability; for instance, motivated by a generalized Rayleigh quotient, we can compute the traces of estimated whitened between-class covariance matrices on the projected data. This enables us to assign an importance weight to each variable for a given projection, and to select our signal variables by aggregating these weights over high-scoring projections. Our theory shows that the resulting Sharp-SSL algorithm is able to recover the signal coordinates with high probability when we aggregate over sufficiently many random projections and when the base procedure estimates the diagonal entries of the whitened between-class covariance matrix sufficiently well. For the Gaussian EM base procedure, we provide a new analysis of its performance in semi-supervised settings that controls the parameter estimation error in terms of the proportion of labeled data in the sample. Numerical results on both simulated data and a real colon tumor dataset support the excellent empirical performance of the method. Supplementary materials for this article are available online, including a standardized description of the materials available for reproducing the work.

## 1 Introduction

Semi-supervised learning, where we attempt to assign observations to one of finitely many groups based on partially-labeled training data, represents a core modern statistical challenge. It is sufficiently general to incorporate, at either extreme, the unsupervised case of no labeled training data (clustering) and the supervised setting of fully-labeled training data (classification). Such tasks abound in many application areas, including genomics (e.g., Eisen et al. 1998), image processing (Jain and Flynn 1996; Cheplygina, de Bruijne, and Pluim 2019), natural language processing (Liang 2005; Turian, Ratinov, and Bengio 2010) and anomaly detection (Akcay, Atapour-Abarghouei, and Breckon 2019; Wang et al. 2019). Entry points to the literature on semi-supervised learning include Zhu (2005), Zhu and Goldberg (2009), Chapelle, Schölkopf, and Zien (2006), and Van Engelen and Hoos (2020). For introductions to clustering, see Xu and Wunsch (2005), Kaufman and Rousseeuw (2009), and Xu and Tian (2015), and for classification, see Devroye, Györfi, and Lugosi (2013) and Hastie, Tibshirani, and Friedman (2009).

A common feature of contemporary semi-supervised learning problems is high-dimensionality, since we may record many covariates having a possible association with the labels. corresponding to different observations. This represents a significant challenge, as can be seen by considering a simple two-class problem with more covariates than observations. For any given assignment of class labels, if no subset of *n*_0_ observations lies in an $\left(n_{0}-2\right)$ -dimensional affine space, then we can find hyperplanes with orthogonal normal vectors, each of which achieves zero training error (in other words, they perfectly separate the classes). Nevertheless, even in the simple setting where the true Bayes decision boundary is linear, many such hyperplanes may be little better than a random guess on test data.

An appealing approach to tackling high-dimensionality is via random projections into lower-dimensional spaces. Such projections may almost preserve the pairwise distances between observations, as seen from the Johnson–Lindenstrauss lemma (Johnson and Lindenstrauss 1984; Dasgupta and Gupta 2003). Moreover, in cases where we have reason to believe that only a relatively small proportion of the variables recorded are relevant for the learning task, we can choose our random projections to be axis-aligned in order to preserve this structure. A third benefit is the possibility of aggregating results over multiple random projections, though this must be done with care so as to avoid noise accumulation. These attractions have meant that random projections have now been employed in many high-dimensional statistical problems, including precision matrix estimation (Marzetta, Tucci, and Simon 2011), two-sample mean testing (Lopes, Jacob, and Wainwright 2011), classification (Durrant and Kabán 2015; Cannings and Samworth 2017), (sparse) principal component analysis (Yang et al. 2021; Gataric, Wang, and Samworth 2020), linear regression (Thanei, Heinze, and Meinshausen 2017; Slawski 2018; Dobriban and Liu 2019; Ahfock, Astle, and Richardson 2021), clustering (Dasgupta 1999; Fern and Brodley 2003; Han and Boutin 2015; Yellamraju and Boutin 2018; Anderlucci, Fortunato, and Montanari 2022) and dimensionality reduction (Bingham and Mannila 2001; Reeve, Kabán, and Bootkrajang 2022). See Cannings (2021) for a review of recent developments in the area.

In this article, we propose a new method, called Sharp-SSL (short for **S**elective **h**igh-dimensional, **a**xis-aligned **r**andom **p**rojections for **S**emi-**S**upervised **L**earning). Our primary goal is to identify a small subset of variables that are particularly helpful for label assignment; existing low-dimensional methods can then be used to complete the learning task. To this end, we generate a large number of axis-aligned random projections, and apply a base learning procedure such as a semi-supervised version of the Gaussian Expectation–Maximization (EM) algorithm to our projected data. We seek to score projections according to their ability to distinguish the classes; for instance, motivated by the notion of a generalized Rayleigh quotient (see (2) for a formal definition), and to avoid the noise accumulation issue mentioned above, we can compute the traces of the corresponding estimated whitened between-class covariance matrices. This enables us to assign an importance weight to each variable for a given projection, and we select our signal variables by aggregating these importance weights over the high-scoring projections. See Section 2 for a more detailed description of our methodology.

Section 3 is devoted to a theoretical analysis of our Sharp-SSL algorithm. We first show in Theorem 2 that provided the low-dimensional base learning procedure estimates the variable importance scores sufficiently well, the corresponding high-dimensional semi-supervised learning algorithm can recover the signal coordinates with high probability when we aggregate over sufficiently many random projections. It turns out that both Linear Discriminant Analysis and an EM algorithm are examples of low-dimensional learning procedures that satisfy this proximity guarantee, as we prove in Theorems 3 and 6, respectively. The latter is particularly challenging, and one of the main novel contributions of our analysis is to provide a guarantee on the performance of a *d*-dimensional Gaussian EM algorithm in a semi-supervised setting. In particular, we control the parameter estimation error in terms of the proportion of labeled data in the sample, showing that with a sample size of *n* it smoothly interpolates between the ${\left(d/n\right)}^{1/4}$ rate for unsupervised learning and the ${\left(d/n\right)}^{1/2}$ rate for fully-labeled data, up to logarithmic factors. An advantage of the modular approach to our analysis is that it illustrates the way in which the Sharp-SSL algorithm can be used with different base learning algorithms to adapt to different problem settings and reflect the preferences of the practitioner.

In Section 4, we study the numerical performance of the Sharp-SSL algorithm. Section 4.1 presents the results of a simulation study involving the Sharp-SSL method, as well as five alternative approaches, on high-dimensional clustering tasks (since not all of the competing methods are able to leverage partial label information). We find that the Sharp-SSL algorithm is able to attain a misclustering rate very close to that of the optimal Bayes classifier, even with only around 50 observations per cluster, in settings where these alternative techniques may perform poorly. In Section 4.2, we investigate the extent to which the different versions of the Sharp-SSL method are able to leverage partial label information. The results here are consistent with the phase transition phenomenon articulated by our theory. Finally, in Section 4.3, we apply the Sharp-SSL algorithm, as well as the other methods from our simulation study, on a colon tumor dataset, where we withhold the true labels from the algorithms in order to assess performance. Our analysis supports the ability of the Sharp-SSL algorithm to identify signal coordinates (genes) that are useful for identifying patients with and without tumors.

In the broader literature on high-dimensional learning, a large number of methods have been developed to leverage sparse low-dimensional structures for both clustering (Witten and Tibshirani 2010; Azizyan, Singh, and Wasserman 2013; Wasserman, Azizyan, and Singh 2014; Azizyan, Singh, and Wasserman 2015; Jin and Wang 2016; Verzelen and Arias-Castro 2017; Löffler, Wein, and Bandeira 2022; Löffler, Zhang, and Zhou 2021) and classification (Cai and Liu 2011; Witten and Tibshirani 2011; Mai, Zou, and Yuan 2012; Cai and Zhang 2019). These methods are not designed for partially labeled (semi-supervised) settings. Another common approach is to project the data into the span of the top few principal components, and run a standard low-dimensional method such as *k*-means clustering or the EM algorithm (Butler et al. 2018). This approach can fail if the directions of largest variation in the data are not aligned with the directions separating the clusters. Finally, recent developments in other aspects of semi-supervised learning include self-training (Oymak and Gulcu 2020), mean estimation (Zhang, Brown, and Cai 2019), choice of *k* in *k*-nearest neighbor classification (Cannings, Berrett, and Samworth 2020) and linear regression (Chakrabortty and Cai 2018).

Proofs of all of our results, as well as some additional simulation results, are provided in the online supplementary material. We conclude this introduction with some notation used throughout the article. We write $S^{d\times d}$ for the set of *d*-dimensional symmetric positive semi-definite matrices, and $S_{+}^{d\times d}$ for the subset that are invertible. We also write $S_{K-1}^{d\times d}$ the subset of matrices in $S^{d\times d}$ of rank at most *K* – 1. For p≥d , let $O^{p\times d}$ denote the set of *p* × *d* matrices with orthonormal columns. For p∈[1,∞] , the $l_{p}$ -norm of a vector *x* is denoted by $\|x{\|}_{p}$ ; we also abbreviate the Euclidean norm of *x* as ‖x‖ . The operator norm of a matrix is denoted by $\|\cdot {\|}_{\text{op}}$ , so that $\left\|A{\|}_{\text{op}}:=\underset{\left\{x:\|x\|=1\right\}}{\text{sup}}\|\text{Ax}\right\|$ . Given two sequences $\left(a_{n}\right)$ and $\left(b_{n}\right)$ , we write $a_{n}≲b_{n}$ when there exists a universal constant *C* > 0 such that $a_{n}\leq Cb_{n}$ , and, given an additional problem parameter *R*, we write $a_{n}{≲}_{R}b_{n}$ when there exists *C* > 0, depending only on *R*, such that $a_{n}\leq Cb_{n}$ . If $S\subseteq R^{d}$ , we define sargmaxS to be the smallest element in the argmax in the lexicographic order. For a positive integer *k*, we define [k]:={1,…,k} . For a vector $v={\left(v_{1},\ldots ,v_{k}\right)}^{⊤}\in R^{k}$ , and j∈[k] , we define $v_{-j}={\left(v_{1},\ldots ,v_{j-1},v_{j+1},\ldots ,v_{k}\right)}^{⊤}\in R^{k-1}$ .

## 2 The Sharp-SSL Algorithm

In this section, we describe in detail the Sharp-SSL algorithm for *K*-class semi-supervised learning, with K≥2 . We aim to provide a unified treatment of clustering, semi-supervised learning and classification. To this end, we assume that for i∈[n] , the observation $x_{i}\in R^{p}$ has a true label $y_{i}^{*}\in [K]$ , but it may be the case that we do not observe $y_{i}^{*}$ . Instead, we assume that our observed label *y_i_* takes values in [K]∪{0} , where $y_{i}:=y_{i}^{*}$ when the true class label is observed, and $y_{i}:=0$ otherwise. Thus, our data can be regarded as $\left(x_{1},y_{1}),\ldots ,(x_{n},y_{n})\in R^{p}\times ([K]\cup \{0\}\right)$ , and our goal is to construct a *data-dependent classifier*^1^, that is a Borel measurable function $C:R^{p}\times {\left(R^{p}\times ([K]\cup \{0\})\right)}^{n}\to [K]$ , with the interpretation that $C(x;(x_{1},y_{1}),\ldots ,(x_{n},y_{n}))$ is the predicted class of $x\in R^{p}$ .

To motivate our Sharp-SSL algorithm, it is instructive first to consider a canonical Gaussian classification problem, where our data can be regarded as *n* independent realizations of a pair (*X*, *Y*) taking values in $R^{p}\times [K]$ , with prior probability $\pi_{k}:=P(Y=k)$ for the *k*th class and $X|Y=k∼N_{p}(\nu_{k},\Sigma_{w})$ , for class means $\nu_{1},\ldots ,\nu_{K}\in R^{p}$ and *within-class covariance matrix* $\Sigma_{w}\in S_{+}^{p\times p}$ . Let $\nu :=\sum_{k=1}^{K}\pi_{k}\nu_{k}\in R^{p}$ denote the grand population mean, let

(1) $$\Sigma_{b}:=\sum_{k=1}^{K}\pi_{k}(\nu_{k}-\nu ){\left(\nu_{k}-\nu \right)}^{⊤}\in S_{K-1}^{p\times p}$$

denote the *between-class covariance matrix*, and consider $D\in O^{p\times (K-1)}$ with a column space spanned by $\left(\Sigma_{w}^{-1}(\nu_{k}-\nu ):k\in [K]\right)$ . Observe that for k≠l , the likelihood ratio

$$\begin{array}{c} \;\text{log}\;\{\frac{P(Y=k|X=x)}{P(Y=l|X=x)}\}\\ =\;\text{log}\;(\frac{\pi_{k}}{\pi_{l}})-\frac{1}{2}{\left(\nu_{k}+\nu_{l}\right)}^{⊤}\Sigma_{w}^{-1}(\nu_{k}-\nu_{l})+x^{⊤}\Sigma_{w}^{-1}(\nu_{k}-\nu_{l}), \end{array}$$

and hence the Bayes classifier $x\mapsto a\text{rgma}x_{k\in [K]}P(Y=k|X=x)$ , only depends on *x* through $D^{⊤}x$ . Thus, for the purposes of classification, no signal would be lost (and the noise would be reduced) if *X* were replaced with $D^{⊤}X$ .

In high-dimensional settings with p≫n , the matrix $\Sigma_{w}^{-1}$ is not consistently estimable in general, but we can nevertheless make progress if the vectors $\Sigma_{w}^{-1}(\nu_{1}-\nu ),\ldots ,\Sigma_{w}^{-1}(\nu_{K}-\nu )$ are sparse. In other words, writing *S*_0_ for the union of the set of coordinates for which these vectors are nonzero, we suppose that $|S_{0}|\ll p$ ; this is a very common assumption in high-dimensional LDA (e.g., Cai and Liu 2011; Witten and Tibshirani 2011; Mai, Zou, and Yuan 2012; Cai and Zhang 2019).

In such a setting, the column space of *D* has a sparse basis, so it is natural to consider projecting the data onto a small subset of its coordinates. For d∈[p] , define the set of axis-aligned projection matrices $P_{d}:=\{P\in {\left\{0,1\right\}}^{d\times p}:PP^{⊤}=I_{d}\}$ , that is the set of binary *d* × *p* matrices with orthonormal rows. We refer to these projections as axis-aligned because each row of any $P\in P_{d}$ contains a single entry equal to 1, with all others equal to zero, so if $x\in R^{p}$ then $\text{Px}\in R^{d}$ simply selects the *d* coordinates of *x* corresponding to the columns of *P* that contain a nonzero entry. By the argument above, if $d\geq |S_{0}|$ then there exists $P^{*}\in P_{d}$ such that the error of the Bayes classifier is unchanged by projecting the data along $P^{*}$ . In practice, it would typically be computationally too expensive to enumerate through all p(p−1)⋯(p−d+1) axis-aligned projections. Instead, we consider a randomly chosen subset of projections within $P_{d}$ . An axis-aligned projection chosen uniformly at random is unlikely to capture all the signal coordinates *S*_0_, but by aggregating over a carefully-chosen subset of these random projections, we can nevertheless recover the set of signal coordinates under suitable conditions; see Theorem 2. To describe our method for choosing good projections, for $V\in O^{p\times d}$ , we define the *generalized Rayleigh quotient* along *V* by

(2) $$J(V;\Sigma_{b},\Sigma_{w}):=\text{tr}\{{\left(V^{⊤}\Sigma_{w}V\right)}^{-1}(V^{⊤}\Sigma_{b}V)\}.$$

Proposition 1 motivates seeking to choose projections to maximize the generalized Rayleigh quotient by showing that the column span of any maximizer $J(V;\Sigma_{b},\Sigma_{w})$ over $V\in O^{p\times d}$ must contain the column space of *D*.

Proposition 1. Let K≥2 and d≥K−1 . Assume that the convex hull of $\nu_{1},\ldots ,\nu_{K}$ is (K−1) -dimensional, and let $V^{*}\in a\text{rgma}x_{V\in O^{p\times d}}J(V;\Sigma_{b},\Sigma_{w})$ . Then the column space of $V^{*}$ contains the eigenspace corresponding to the *K* – 1 nonzero eigenvalues^2^ of $\Sigma_{w}^{-1}\Sigma_{b}$ , which is equal to the space spanned by $\left(\Sigma_{w}^{-1}(\nu_{k}-\nu ):k\in [K]\right)$ .

Based on Proposition 1, a natural conceptual approach to maximizing the generalized Rayleigh quotient is to compute the leading (K−1) -dimensional eigenspace of $\Sigma_{w}^{-1}\Sigma_{b}$ . This strategy, however, runs into difficulties when we replace these population quantities with their sample versions in the setting of the opening paragraph of this section. More precisely, writing $n_{k}:=\sum_{i=1}^{n}1_{\left\{y_{i}=k\right\}}$ for k∈[K] , as well as ${\tilde{\Sigma }}_{w}:=\frac{1}{n}\sum_{k=1}^{K}\sum_{i=1}^{n}(x_{i}-{\hat{\nu }}_{k}){\left(x_{i}-{\hat{\nu }}_{k}\right)}^{⊤}1_{\left\{y_{i}=k\right\}}\in R^{p\times p}$ and ${\tilde{\Sigma }}_{b}:=\sum_{k=1}^{K}\frac{n_{k}}{n}({\hat{\nu }}_{k}-\hat{\nu }){\left({\hat{\nu }}_{k}-\hat{\nu }\right)}^{⊤}\in R^{p\times p},$ for the sample versions of the within-class and between-class covariance matrices, respectively, the matrix ${\tilde{\Sigma }}_{w}$ is not invertible whenever *p* > *n*. Fortunately, though, this issue can be resolved by working with the projected data, as long as we choose d≤n−K : the projected data $\left\{PX_{i}:i\in [n]\right\}$ has within-class covariance matrix $P\Sigma_{w}P^{⊤}\in R^{d\times d}$ and between-class covariance matrix $P\Sigma_{b}P^{⊤}\in R^{d\times d}$ , so with probability one, the sample version $P{\tilde{\Sigma }}_{w}P^{⊤}$ is invertible.

Returning to the general setting of the opening paragraph of this section, then, we seek projections *P* with large $J(P^{⊤};{\tilde{\Sigma }}_{b},{\tilde{\Sigma }}_{w})=\text{tr}\{{\left(P{\tilde{\Sigma }}_{w}P^{⊤}\right)}^{-1}(P{\tilde{\Sigma }}_{b}P^{⊤})\}$ . To this end, for fixed A,B∈N , we sample a set of axis-aligned projections $\left\{P^{a,b}:a\in [A],b\in [B]\right\}$ uniformly at random from $P_{d}$ . We further assume that we have access to a base algorithm $\psi :{\left(R^{d}\times ([K]\cup \{0\})\right)}^{n}\to {\left[0,\infty \right)}^{d}$ , which takes low-dimensional semi-supervised data as an input and returns a vector of estimated importance scores for distinguishing the classes for each of the variables. We suppose throughout for convenience that *ψ* is *permutation equivariant* in the sense that $\psi ((\Pi z_{1},y_{1}),\ldots ,(\Pi z_{n},y_{n}))=\Pi \psi ((z_{1},y_{1}),\ldots ,(z_{n},y_{n}))$ for every permutation matrix $\Pi \in R^{d\times d}$ . By applying *ψ* to the projected data $\left(P^{a,b}x_{1},y_{1}),\ldots ,(P^{a,b}x_{n},y_{n}\right)$ , we obtain for each *a* and *b* an estimator ${\hat{w}}^{a,b}$ of the projected importance scores. In Sections 3.2 and 3.3, we take *ψ* to be the operator that when applied to the projected data returns the diagonal of the *whitened between-class (projected) covariance matrix* ${\left(P^{a,b}\Sigma_{w}P^{a,b,⊤}\right)}^{-1}(P^{a,b}\Sigma_{b}P^{a,b,⊤})$ .

To choose projections, for each a∈[A] , we define $b^{*}(a):={\text{sargmax}}_{b\in [B]}\sum_{j=1}^{d}{\hat{w}}_{j}^{a,b}$ to be the projection within the *a*th group with the largest sum of importance scores, and select $P^{a,b^{*}(a)}$ . The main rationale for dividing the projections into *A* groups and selecting one within each group—as opposed to selecting the *A* projections with the largest sum of importance scores—is that, conditional on the original data, the selected projections are independent and identically distributed. This facilitates our theoretical analysis by enabling the application of concentration inequalities in the proof of Theorem 2.

The selected importance scores $\left\{{\hat{w}}^{a,b^{*}(a)}:a\in [A]\right\}$ within each group can be “back-projected” into the original higher-dimensional space and aggregated to form the overall vector of importance scores $\hat{w}:=\frac{1}{A}\sum_{a=1}^{A}P^{a,b^{*}(a),⊤}{\hat{w}}^{a,b^{*}(a)}\in {\left[0,\infty \right)}^{p}.$ Finally, we rank the *p* variables by their overall importance scores, and our estimate $\hat{S}$ of the set of signal coordinates is given by the largest l entries in $\hat{w}$ , breaking ties arbitrarily if necessary, where l∈[p] is specified by the practitioner. Pseudocode for the variable selection aspect of the Sharp-SSL procedure is given in Algorithm 1.

Algorithm 1: Variable selection via ensembles of axis-aligned random projections.

**Input:** $\left(x_{1},y_{1}),\ldots ,(x_{n},y_{n})\in R^{p}\times ([K]\cup \{0\}\right)$ (*y_i_* = 0 denotes a missing label);

Projected dimension d∈[min(p,n−K)] , number of selected signal coordinates l∈[p] ;

A,B∈N for groups of projections and number of projections per group;

Permutation equivariant base algorithm $\psi :{\left(R^{d}\times ([K]\cup \{0\})\right)}^{n}\to {\left[0,\infty \right)}^{d}$ .

Generate axis-aligned random projections $\left\{P^{a,b}:a\in [A],b\in [B]\right\}$ independently and uniformly from $P_{d}$ .**for** a∈[A] **do for** b∈[B] **do**

Let ${\hat{w}}^{a,b}:=\psi ((P^{a,b}x_{1},y_{1}),\ldots ,(P^{a,b}x_{n},y_{n}))$ .**end**

Set $b^{*}(a):={\text{sargmax}}_{b\in [B]}\sum_{j=1}^{d}{\hat{w}}_{j}^{a,b}$ .**end**

Let $\hat{w}:=A^{-1}\sum_{a=1}^{A}P^{a,b^{*}(a),⊤}{\hat{w}}^{a,b^{*}(a)}$ .

**Output:** $\hat{S}\subseteq [p]$ , defined as the index set of the l largest components of $\hat{w}$ .

After applying Algorithm 1 to obtain an estimated set $\hat{S}$ of signal variables, the Sharp-SSL procedure then applies any existing low-dimensional semi-supervised learning method with input ${\left(P_{\hat{S}}x_{i,},y_{i}\right)}_{i\in [n]}$ , where $P_{\hat{S}}$ is the projection onto the coordinates in $\hat{S}$ .

Algorithm 2: Base learning using only labeled data

**Input:** $\left(z_{1},y_{1}),\ldots ,(z_{n},y_{n})\in R^{d}\times ([K]\cup \{0\}\right)$ . A closed constraint set $C\subseteq S^{d\times d}$ , with default $C=S^{d\times d}$ .**for** k∈[K] **do**

Set $n_{k}:=|\{i:y_{i}=k\}|$ and ${\hat{\mu }}_{k}:=n_{k}^{-1}\sum_{i:y_{i}=k}z_{i}$ (if *n_k_* = 0, then set ${\hat{\mu }}_{k}:=0$ ).**end**

Compute $n\prime :=\sum_{k=1}^{K}n_{k}$ and $\hat{\mu }:={\left(n\prime \right)}^{-1}\sum_{i=1}^{n}z_{i}$ . Compute the within-class and between-class covariance matrices as

(3) $${\hat{\Gamma }}_{w}:=\frac{1}{n\prime }\sum_{i=1}^{n}(z_{i}-{\hat{\mu }}_{y_{i}}){\left(z_{i}-{\hat{\mu }}_{y_{i}}\right)}^{⊤}\;\text{and}\;{\hat{\Gamma }}_{b}:=\sum_{k=1}^{K}\frac{n_{k}}{n\prime }({\hat{\mu }}_{k}-\hat{\mu }){\left({\hat{\mu }}_{k}-\hat{\mu }\right)}^{⊤}.$$

**Output:** $\psi ({\left(z_{i},y_{i}\right)}_{i\in [n]}):={\left({\left({\left\{{\text{Proj}}_{C}{\hat{\Gamma }}_{w}\right\}}^{-1}{\hat{\Gamma }}_{b}\right)}_{j,j}\right)}_{j=1}^{d}$ , where ${\text{Proj}}_{C}:S^{d\times d}\to C$ denotes the Euclidean projection operator onto C ; here we take the pseudoinverse if ${\text{Proj}}_{C}{\hat{\Gamma }}_{w}$ is not invertible.

### 2.1 Base Learning Methods

Algorithm 1 relies on a base learning method for low-dimensional data to estimate the diagonal of the projected whitened between-class covariance matrix from the projected data. When all or almost all of the input data are labeled, we can use the procedure outlined in Algorithm 2, which ignores any unlabeled data, for this purpose. On the other hand, when we have a substantial amount of unlabeled data, Algorithm 2 may be inaccurate. In such circumstances, it may be preferable to use Algorithm 3, which runs an *Expectation–Maximization* (EM) procedure to predict the unobserved labels and subsequently estimate the whitened between-class covariance matrix and its diagonal. More precisely, from *M* random initializations of the cluster means and the within-class covariance matrix, Algorithm 3 uses the EM algorithm to update these quantities, and thereby compute the whitened between-cluster sample covariance matrix estimators $\left\{{\hat{Q}}^{\left[m\right]}:m\in [M]\right\}$ . We select $\hat{m}\in [M]$ such that ${\hat{Q}}^{\left[\hat{m}\right]}$ is in best agreement with results from the other EM runs.

Algorithm 3 also allows the practitioner to incorporate prior knowledge about the true cluster means and within-cluster covariance matrices, both through optimizing over a restricted constraint set C in the M step of the EM algorithm, and through the choice of a distribution supported on C for the initialization of these quantities. An alternative to the EM algorithm for unsupervised learning would be to apply *k*-means clustering as a base procedure. Previous studies have suggested that these approaches have comparable empirical performance (e.g., de Souto et al. 2008; Rodriguez et al. 2019, and references therein), but the EM algorithm is more amenable to theoretical analysis in our setting.

Algorithm 3: Base learning using partially labeled data via an EM algorithm

**Input:** Data $\left(z_{1},y_{1}),\ldots ,(z_{n},y_{n})\in R^{d}\times ([K]\cup \{0\}\right)$ . A constraint set $C\subseteq {\left(R^{d}\right)}^{K}\times S^{d\times d}$ and a probability distribution $\pi_{C}$ supported on C . Number of random initializations *M*. Number of iterations *T*.**for** m∈[M] **do**

Randomly sample $({\hat{\mu }}_{1},\ldots ,{\hat{\mu }}_{K},{\hat{\Gamma }}_{w})∼\pi_{C}$ .**for** t∈[T] **do**

(E step) Compute the soft-label matrix ${\left(L_{i,k}\right)}_{i\in [n],k\in [K]}$ with entries

(4) $$L_{i,k}:=(\frac{e^{-\frac{1}{2}{\left(z_{i}-{\hat{\mu }}_{k}\right)}^{⊤}{\hat{\Gamma }}_{w}^{-1}(z_{i}-{\hat{\mu }}_{k})}}{\sum_{l=1}^{K}e^{-\frac{1}{2}{\left(z_{i}-{\hat{\mu }}_{l}\right)}^{⊤}{\hat{\Gamma }}_{w}^{-1}(z_{i}-{\hat{\mu }}_{l})}})1_{\left\{y_{i}=0\right\}}+1_{\left\{y_{i}=k\right\}}.$$

(M step) Update parameter estimates by

(5) $$({\hat{\mu }}_{1},\ldots ,{\hat{\mu }}_{K},{\hat{\Gamma }}_{w}):={\text{argmin}}_{(\mu_{1},\ldots ,\mu_{K},\Gamma )\in C}\{\frac{1}{n}\sum_{i=1}^{n}\sum_{k=1}^{K}L_{i,k}{\left(z_{i}-\mu_{k}\right)}^{⊤}\Gamma^{-1}(z_{i}-\mu_{k})+\;\text{log det}\Gamma \}.$$

**end**

Compute ${\left(L_{i,k}\right)}_{i\in [n],k\in [K]}$ using the final values of $\left({\hat{\mu }}_{1},\ldots ,{\hat{\mu }}_{K},{\hat{\Gamma }}_{w}\right)$ as in (4).

Set ${\hat{\mu }}_{\text{tot}}:=\frac{1}{n}\sum_{i=1}^{n}\sum_{k=1}^{K}L_{i,k}{\hat{\mu }}_{k}$ and ${\hat{\Gamma }}_{b}:=\frac{1}{n}\sum_{i=1}^{n}\sum_{k=1}^{K}L_{i,k}({\hat{\mu }}_{k}‐{\hat{\mu }}_{\text{tot}}){\left({\hat{\mu }}_{k}‐{\hat{\mu }}_{\text{tot}}\right)}^{⊤}.$

Set ${\hat{Q}}^{\left[m\right]}:={\hat{\Gamma }}_{w}^{-1}{\hat{\Gamma }}_{b}$ .**end**

Set $\hat{m}\in {\text{argmin}}_{m\in [M]}\text{median}(\|{\hat{Q}}^{\left[m\right]}-{\hat{Q}}^{\left[m\prime \right]}{\|}_{\text{op}}:m\prime \in [M]\setminus \{m\})$ and $\hat{Q}:={\hat{Q}}^{\left[\hat{m}\right]}$ .

**Output:** ${\left({\hat{Q}}_{j,j}\right)}_{j=1}^{d}$ .

## 3 Theoretical Guarantees

### 3.1 Results for the High-Level Algorithm

Here we consider independent triples $\left(X_{1},Y_{1},Y_{1}^{*}),\ldots ,(X_{n},Y_{n},Y_{n}^{*}\right)$ taking values in $R^{p}\times ([K]\cup \{0\})\times [K]$ . We recall that $Y_{i}^{*}$ denotes the true label of the *i*th observation, and that $Y_{i}:=Y_{i}^{*}$ if the *i*th label is observed, and $Y_{i}:=0$ otherwise. For a fixed vector $w={\left(w_{1},\ldots ,w_{p}\right)}^{⊤}\in R^{p}$ , we define $S_{0}:=\{j\in [p]:w_{j}>0\},$ and write $s_{0}:=|S_{0}|$ . As we will see later in Sections 3.2 and 3.3, in specific applications, *w* will be defined to be a direction that best distinguishes different classes/clusters and *S*_0_ can be interpreted as the set of signal coordinates.

Our first main theoretical result shows that if the base algorithm is accurate on each low-dimensional projection and *A* is large, then with high probability, all signal coordinates are selected.

Theorem 2. Define $\gamma_{\text{min}}:={\text{min}}_{j\in S_{0}}w_{j}$ and $\gamma_{\text{max}}:={\text{max}}_{j\in S_{0}}w_{j}$ . Let $\hat{S}$ be the output of Algorithm 1 with input *K*, *p*, $\left(X_{1},Y_{1}),\ldots ,(X_{n},Y_{n}\right)$ , *A*, *B*, $s_{0}\leq d\leq \text{min}(p,n-K),\;l\geq s_{0}$ and permutation equivariant base procedure *ψ*. For $P\in P_{d}$ , write ${\hat{w}}^{P}:=\psi ({\left(PX_{i},Y_{i}\right)}_{i\in [n]})$ and

(6) $$\varepsilon :=P({\text{max}}_{P\in P_{d}}\|{\hat{w}}^{P}-\text{Pw}{\|}_{1}\geq \frac{\gamma_{\text{min}}}{4}).$$

Then $P(S_{0}\subseteq \hat{S})\geq 1-\varepsilon -pe^{-A\gamma_{{\text{min}}^{2}}/(50p^{2}\gamma_{{\text{max}}^{2}})}.$

In fact, we can see from the proof of Theorem 2 that the following stronger conclusion holds: for any realization ${\left(x_{i},y_{i}\right)}_{i\in [n]}$ of the data satisfying ${\text{max}}_{P\in P_{d}}\|{\hat{w}}^{P}-\text{Pw}{\|}_{1}<\gamma_{\text{min}}/4$ , we have $P(S_{0}\subseteq \hat{S}|{\left(X_{i},Y_{i}\right)}_{i\in [n]}={\left(x_{i},y_{i}\right)}_{i\in [n]})\geq 1-pe^{-A\gamma_{{\text{min}}^{2}}/(50p^{2}\gamma_{{\text{max}}^{2}})}$ . Note here that, after conditioning on the data, the probability is taken over the randomness in the projections. An attraction of Theorem 2 is its generality, and in particular the fact that we do not impose strong distributional assumptions—we simply require control of *ε* in (6). The price we pay for this generality is that the probability bound may be loose in particular cases; for example, the bound holds even with *B* = 1, though in practice we would expect it to improve as *B* increases, at least for small values of *B*.

### 3.2 Theory for Base Learning Using Labeled Data

In this subsection, for k∈[K] , let $\pi_{k}:=P(Y_{1}^{*}=k)$ and $\nu_{k}^{*}:=E(X_{1}|Y_{1}^{*}=k)$ denote the prior probability and the cluster mean of the *k*th cluster, respectively. Let $\nu^{*}:=\sum_{k=1}^{K}\pi_{k}\nu_{k}^{*}$ denote the weighted cluster mean and let $\Sigma_{w}:=\text{Cov}(X_{1}|Y_{1}^{*}=k)$ denote the common within-cluster covariance matrix. We demonstrate how the high-level result in Theorem 2 can be used to derive performance guarantees for the estimated variable importance scores in a high-dimensional classification setting where we apply Algorithm 1 in conjunction with the low-dimensional base method described in Algorithm 2.

Algorithm 2 takes as an input a closed constraint set $C\subseteq S^{d\times d}$ . This allows the user to impose prior knowledge on the structure of the within-class covariance matrix of our low-dimensional (projected) data, by outputting the diagonal of ${\left\{{\text{Proj}}_{C}{\hat{\Gamma }}_{w}\right\}}^{-1}{\hat{\Gamma }}_{b}$ . The following theorem provides uniform control of the output of Algorithm 2 for all axis-aligned *d*-dimensional projected datasets when C is the set of *d* × *d* diagonal positive semidefinite matrices. For positive integers n,d,p,K with p≥d and ε>0 , we denote

(7) $$E(n,d,p,K,\varepsilon ):=\frac{K}{n}+\sqrt{\frac{\;\text{log}\;(8d\left(\begin{array}{c} p\\ d \end{array}\right))+\;\text{log}\;(1/\varepsilon )}{n}}.$$

Theorem 3. Fix ε∈(0,1], K∈{2,3,…,} , and p,d∈N with p≥d . Suppose that $\left(X_{1},Y_{1}),\ldots ,(X_{n},Y_{n}\right)$ are independent and identically distributed pairs, with $P(Y_{1}=k)=\pi_{k}$ and $X_{1}|Y_{1}=k∼N_{p}(\nu_{k}^{*},\Sigma_{w})$ for k∈[K] , and let $\psi ({\left(PX_{i},Y_{i}\right)}_{i\in [n]})$ denote the output of Algorithm 2 with input ${\left(PX_{i},Y_{i}\right)}_{i\in [n]}$ , for $P\in P_{d}$ , and C as the set of *d* × *d* diagonal positive semi-definite matrices. Suppose that ${\text{max}}_{k\in [K]}\|\nu_{k}^{*}-\nu^{*}{\|}_{\infty }\leq R_{1}$ for some $R_{1}>0$ , and that $\Sigma_{w}$ is diagonal and well-conditioned in the sense that $\text{max}\{\|\Sigma_{w}{\|}_{\text{op}},\|\Sigma_{w}^{-1}{\|}_{\text{op}}\}\leq R_{2}$ for some $R_{2}\geq 1$ . Then there exists $c_{1}>0$ , depending only on *R*_1_ and *R*_2_, such that if $E(n,d,p,K,\varepsilon )\leq c_{1}$ , then for $\Sigma_{b}=\sum_{k=1}^{K}\pi_{k}(\nu_{k}^{*}-\nu^{*}){\left(\nu_{k}^{*}-\nu^{*}\right)}^{⊤}$ and $w^{P}={\left({\left(P\Sigma_{w}^{-1}\Sigma_{b}P^{⊤}\right)}_{j,j}\right)}_{j=1}^{d}$ , we have with probability at least 1−ε that

$${\text{max}}_{P\in P_{d}}\|\psi ({\left(PX_{i},Y_{i}\right)}_{i\in [n]})-w^{P}{\|}_{\infty }{≲}_{R_{1},R_{2}}E(n,d,p,K,\varepsilon ).$$

We remark that in the setting of Theorem 3, $\Sigma_{w}^{-1/2}(\nu_{k}^{*}-\nu_{k\prime }^{*})$ is parallel to the linear discriminant direction distinguishing between class *k* and class k′ . Hence, $w:={\left({\left(\Sigma_{w}^{-1}\Sigma_{b}\right)}_{j,j}\right)}_{j=1}^{p}$ can be viewed as an entrywise weighted sum of squares of all pairwise linear discriminant direction vectors for classification. In fact, there is a sense in which $w_{j}>w_{j\prime }$ if *j* is a more important variable than j′ . Indeed, focusing on the two-class setting for simplicity, the sum of the components of *w* is the Mahalanobis distance between the classes, and if we project the data onto the *j*th coordinate, then the Bayes risk in the resulting one-dimensional problem is a decreasing function of *w_j_*. This follows because the Bayes risk is $\pi_{1}\Phi (-w_{j}^{1/2}/2-\;\text{log}\;(\pi_{1}/\pi_{2})/w_{j}^{1/2})+\pi_{2}\Phi (-w_{j}^{1/2}/2+\;\text{log}\;(\pi_{1}/\pi_{2})/w_{j}^{1/2})$ , where *π*_1_ and *π*_2_ are prior probabilities of the respective classes and Φ denotes the standard normal distribution function. Furthermore, by the argument following (1), if we reduce our covariates to their coordinates in *S*_0_ (i.e., where the corresponding components of *w* are nonzero), then the Bayes risk is unaffected. The vector *w^P^* in Theorem 3 is the restriction of *w* under the projection *P*.

The sample size condition $E(n,d,p,K,\varepsilon )\leq c_{1}$ is implied by $n{≳}_{R_{1},R_{2}}d\;\text{log}\;(p/d)+\;\text{log}\;(d/\varepsilon )+K$ , so may be regarded as mild. Thinking of *K* as a constant, Theorem 3 confirms that the uniform control of Algorithm 2 is at the parametric rate, up to a logarithmic factor. The following corollary then follows immediately by combining Theorems 2 and 3.

Corollary 4. Fix ε∈(0,1] . Suppose that the conditions of Theorem 3 hold, and moreover that ${\text{min}}_{j\in [p]}{\left(\Sigma_{b}\right)}_{j,j}\geq 1/R_{3}$ for some $R_{3}>0$ . Define the set of signal coordinates $S_{0}:=\{j\in [p]:{\left(\Sigma_{w}^{-1}\Sigma_{b}\right)}_{j,j}>0\}$ and $s_{0}:=|S_{0}|$ . Then there exist $C_{1},C_{2}>0$ , depending only on *R*_1_, *R*_2_ and *R*_3_, such that if $C_{1}E(n,d,p,K,\varepsilon )\leq 1/d$ , then the output $\hat{S}$ of Algorithm 1 with input *K*, *p*, $s_{0}\leq d\leq \text{min}(p,n-K),\;l\geq s_{0},\;(X_{1},Y_{1}),\ldots ,(X_{n},Y_{n})$ , *A*, *B*, the set C of *d* × *d* diagonal positive semi-definite matrices, and base procedure *ψ* from Algorithm 2 satisfies

(8) $$P(S_{0}\subseteq \hat{S})\geq 1-\varepsilon -p\;\text{exp}\;(-\frac{A}{C_{2}p^{2}}).$$

Thus, under the conditions of Corollary 4, the Sharp-SSL algorithm can, with high probability, select all of the signal variables, provided that the number *A* of groups of random projections is large by comparison with *p*^2^. In other words, the algorithm reduces the problem to a low-dimensional one, for which standard learning techniques can be applied. The guarantees for these methods (e.g., Anderson 2003, Theorem 6.6.1) can then be combined on the high-probability event of Corollary 4 to establish theoretical results for the full procedure. Further, in Section S2, we provide an algorithm and analysis for the more general case where we allow the within-class covariance matrices to be different for different classes.

### 3.3 Theory for Semi-Supervised Base Learning

When the proportion of labeled data is low, Algorithm 2 may be inaccurate when used as the base procedure in Algorithm 1. The aim of this subsection, therefore, is to study the base procedure of Algorithm 3, which is able to leverage both the labeled and unlabeled data via an EM algorithm to estimate variable importance scores for each projected dataset. Our analysis builds on several recent breakthroughs in our understanding of the EM algorithm. This line of work includes Balakrishnan, Wainwright, and Yu (2017), Daskalakis, Tzamos, and Zampetakis (2017), Yan, Yin, and Sarkar (2017), Dwivedi et al. (2020a), Dwivedi et al. (2020b), Minsker, Ndaoud, and Shen (2021), Ho et al. (2020), Ndaoud (2022), Wu and Zhou (2022), and Doss et al. (2023), all of which focus on the unsupervised case. While our main focus in this section is on the EM algorithm, we also mention that a similar semi-supervised procedure could be developed based on Lloyd’s algorithm for *k*-means clustering. We refer to the recent works of Lu and Zhou (2016) and Ndaoud (2022) for theoretical analyses of Lloyd’s algorithm.

For simplicity, we will focus on the setting where independent and identically distributed $\left(X_{1},Y_{1}^{*}),\ldots ,(X_{n},Y_{n}^{*}\right)$ are generated from a mixture of two Gaussians with opposite means and identity covariance matrix:

(9) $$\begin{array}{c} Y_{i}^{*}∼\text{Unif}(\{1,2\}),X_{i}|Y_{i}^{*}∼N_{p}({\left(-1\right)}^{Y_{i}^{*}}\nu^{*},I_{p}),\;\text{and}\\ Y_{i}=Y_{i}^{*}1_{\left\{i\leq n_{L}\right\}}\text{for all }i\in [n]. \end{array}$$

We assume that we observe $(X_{1},Y_{1}),\ldots ,(X_{n_{L}},Y_{n_{L}}),X_{n_{L}+1},\ldots ,X_{n}$ for some $n_{L}\in \{0,\ldots ,n\}$ . In other words, we are given $n_{L}$ labeled observations and $n_{U}:=n-n_{L}$ unlabeled ones. Thus, $n_{L}=0$ corresponds to the fully unsupervised case, that is, clustering, while $n_{L}=n$ corresponds to the supervised case, that is, classification. We define $Y_{i}=Y_{i}^{*}$ for $i\in [n_{L}]$ , and *Y _i_* = 0 for $i\in \{n_{L}+1,\ldots ,n\}$ . In this setup, if all labels are known, then $\nu^{*}$ is the optimal (linear discriminant) direction for distinguishing the two clusters. Algorithm 1 using Algorithm 3 as the base procedure can be used to recover the nonzero coordinates of $w:={\left({\left(\nu_{j}^{*}\right)}^{2}\right)}_{j=1}^{p}$ .

In addition to allowing more general class-conditional covariance matrices, it would be of interest to extend our methodology beyond the Gaussian setting. Recent work on spectral estimation of sub-Gaussian mixtures in the unsupervised case includes Abbe, Fan, and Wang (2022) and Zhang and Zhou (2022). Although sub-Gaussianity, which only controls tail behavior, is insufficient to guarantee the existence of a maximum likelihood estimator, one could also consider other global constraints such as log-concavity (Walther 2002; Samworth 2018). Indeed, Cule, Samworth, and Stewart (2010) considered a log-concave EM algorithm for fitting finite mixtures of (low-dimensional) log-concave densities but did not study the theoretical properties of this algorithm. One significant issue is related to identifiability: for instance, writing $\phi_{d}$ for the standard *d*-dimensional Gaussian density, the mixture density $\pi_{1}\phi_{d}(\cdot -\mu )+(1-\pi_{1})\phi_{d}(\cdot +\mu )$ with $\pi_{1}\in [0,1]$ is itself log-concave whenever ‖μ‖≤1 . See Balabdaoui and Doss (2018) for a univariate EM algorithm in the context of a two-component symmetric log-concave mixture.

As in Section 3.2, to understand the performance of the sharp-SSL algorithm in this setting, we first study the performance of the EM procedure after the covariates have been projected into a lower-dimensional space. In other words, for some fixed $P\in P_{d}$ , define $Z_{i}:=PX_{i}$ for i∈[n] and $\mu^{*}:=P\nu^{*}\in R^{d}$ , so that $Z_{i}|Y_{i}^{*}∼N_{d}({\left(-1\right)}^{Y_{i}^{*}}\mu^{*},I_{d})$ . In this setting, we have a single unknown parameter $\mu^{*}$ to estimate, and this can be achieved by applying Algorithm 3 to ${\left(Z_{i},Y_{i}\right)}_{i\in [n]}$ with *K* = 2 and the constraint set

(10) $$C:=\{(-\mu ,\mu ,I_{d}):\mu \in R^{d}\}.$$

After initializing the EM algorithm at some fixed $(-{\hat{\mu }}^{\left(0\right)},{\hat{\mu }}^{\left(0\right)},I_{d})\in C$ , for t∈N , the *t*th iterate of the EM iteration described in (4) and (5) is $\left(-{\hat{\mu }}^{\left(t\right)},{\hat{\mu }}^{\left(t\right)},I_{d}\right)$ , where

(11) $${\hat{\mu }}^{\left(t\right)}:=\frac{1}{n}\{\sum_{i:Y_{i}\neq 0}{\left(-1\right)}^{Y_{i}}Z_{i}+\sum_{i:Y_{i}=0}Z_{i}\text{tanh}〈Z_{i},{\hat{\mu }}^{\left(t-1\right)}〉\};$$

see Lemma S10. Since we allow $n_{L}=0$ , where *μ* is only identifiable up to sign, and since the between-class sample covariance matrix ${\hat{\Gamma }}_{b}$ computed in Algorithm 3 is equal to ${\hat{\Gamma }}_{b}={\hat{\mu }}_{1}{\hat{\mu }}_{1}^{⊤}-{\hat{\mu }}_{\text{tot}}{\hat{\mu }}_{\text{tot}}^{⊤}$ , which is invariant to flipping the signs of ${\hat{\mu }}_{1}$ and ${\hat{\mu }}_{2}$ simultaneously, it is natural to consider the loss function $L:R^{d}\times R^{d}\to [0,\infty )$ given by

L(μ,μ′):=‖μ−μ′‖∧‖μ+μ′‖.

Proposition 5 provides a theoretical guarantee for this semi-supervised EM algorithm. For notational simplicity, we define $\gamma :=n_{L}/n$ , $\omega_{0}:=\sqrt{\{d\;\text{log}\;n+\;\text{log}\;(1/\delta )\}/n_{U}}$ and $\zeta_{0}:=\text{min}\{\omega_{0}\gamma^{-1/2},\omega_{0}^{1/2}\}$ throughout this section. Thus, treating *d* as a constant and ignoring polylogarithmic terms, *ω*_0_ is of order $n_{U}^{-1/2}$ and *ζ*_0_ is of order $\text{min}\{n_{L}^{-1/2},n_{U}^{-1/4}\}$ when γ<1/2 . We remark that $n_{L}^{-1/2}$ is the critical $l_{2}$ -testing radius for distinguishing the means of two labeled Gaussian distributions with identity covariance using $n_{L}$ observations. On the other hand, as we show in Lemma S11, no test of the null hypothesis $H_{0}:N_{d}(0,I_{d})$ against the two-component mixture alternative $H_{1}:\frac{1}{2}N_{d}(\mu^{*},I_{d})+\frac{1}{2}N_{d}(-\mu^{*},I_{d})$ based on $n_{U}$ observations can have large power unless the signal strength $\left\|\mu^{*}\right\|$ is at least of order $n_{U}^{-1/4}$ .

Proposition 5. Fix $\delta \in (2e^{-n},1]$ and r≥1 , and suppose that $\|\mu^{*}\|\leq r$ and γ<1/2 . There exists *c* > 0, depending only on *r*, such that if $\omega_{0}\leq c$ and n≥3 , then the following statements hold:

- For any ${\hat{\mu }}^{\left(0\right)}\in R^{d}$ with $\|{\hat{\mu }}^{\left(0\right)}\|\leq r+3$ , we have with probability at least 1−2δ that $\underset{t\to \infty }{\text{limsup}}L({\hat{\mu }}^{\left(t\right)},\mu^{*}){≲}_{r}\zeta_{0}\vee \|\mu^{*}\|.$
- There exists *C* > 0, depending only on *r*, such that if $\|\mu^{*}\|\geq C\zeta_{0}\sqrt{d\;\text{log}\;n}$ and ${\hat{\mu }}^{\left(0\right)}=(\zeta_{0}\vee r\omega_{0})\eta_{0}$ with $\eta_{0}∼\text{Unif}(S^{d-1})$ , then with probability at least $1-2\delta -\sqrt{2/(\pi \;\text{log}\;n_{U})}$ , we have $\underset{t\to \infty }{\text{limsup}}L({\hat{\mu }}^{\left(t\right)},\mu^{*}){≲}_{r}\frac{\omega_{0}}{\left\|\mu^{*}\right\|}\wedge \frac{\omega_{0}}{\gamma^{1/2}}.$

In order to interpret Proposition 5*(i)*, consider the regime where $\|\mu^{*}\|\leq \zeta_{0}$ . In this case, as discussed above, the two mixture components are essentially indistinguishable, and the bound reveals that the EM algorithm performs no worse than the trivial zero estimator, up to constant factors. On the other hand, part *(ii)* studies the more interesting regime where the two mixture components are distinguishable, and we establish a faster convergence rate for the EM algorithm in this strong signal regime.

The following theorem combines the two convergence regimes in Proposition 5 to derive a convergence guarantee for the estimated variable importance scores output by Algorithm 3. To state the result, recall the definition of C from (10). For any ζ>0 , we write U(ζ) for the pushforward measure on C induced by $\text{Unif}(\zeta S^{d-1})$ under the map $\mu \mapsto (-\mu ,\mu ,I_{d})$ .

Theorem 6. Fix $\delta \in (2e^{-n},1]$ , and r≥1 and suppose that $\|\mu^{*}\|\leq r$ and γ<1/2 . There exists *c* > 0, depending only on *r*, such that if $\omega_{0}\leq \text{min}\{c,{\left(d\;\text{log}\;n\right)}^{-3}\}$ and n≥108 , then the sequence of outputs ${\left({\hat{Q}}^{\left(T\right)}\right)}_{T\in N}$ of Algorithm 3 with inputs $(Z_{1},Y_{1}),\ldots ,(Z_{n},Y_{n}),\;C,\;\pi_{C}=U(\zeta_{0}\vee r\omega_{0}),\;M\in N$ and T∈N satisfies with probability at least $1-3\delta -e^{-M/50}$ that

$$\underset{T\to \infty }{\text{limsup}}{\text{max}}_{j\in [d]}|{\hat{Q}}_{j,j}^{\left(T\right)}-{\left(\mu_{j}^{*}\right)}^{2}|{≲}_{r}\frac{\omega_{0}}{\left\|\mu^{*}\right\|}\wedge \zeta_{0}.$$

Finally in this section, we study the implications of Theorem 6 for the recovery of the signal coordinates, that is the nonzero coordinates of $\nu^{*}\in R^{p}$ , in the semi-supervised learning setting. Recalling the definition of *w* in the second paragraph of this subsection, we write $S_{0}:=\{j:w_{j}\neq 0\}=\{j:\nu_{j}^{*}\neq 0\}$ and let $s_{0}:=|S_{0}|$ . We write $\psi^{\left(M,T\right)}$ for the base procedure that takes ${\left(z_{i},y_{i}\right)}_{i\in [n]}\in R^{d}\times ([K]\cup \{0\})$ as input and returns ${\left({\hat{Q}}_{j,j}\right)}_{j=1}^{d}$ , where $\hat{Q}$ is the output of Algorithm 3 when run with these inputs together with $C,\;\pi_{C}$ , *M*, and *T*.

Corollary 7. Fix $\varepsilon \in (8e^{-n/2},1]$ , r≥1 , and suppose that $\|\mu^{*}\|\leq r,\;M\geq 50\;\text{log}\;(4/\varepsilon )+50d\;\text{log}\;p$ and γ<1/2 . Let $\nu_{\text{max}}^{*}:=\|\nu^{*}{\|}_{\infty }$ and let $\nu_{{\text{min}}^{*}}$ denote the minimum absolute value of a nonzero component of $\nu^{*}$ . There exist $C_{1},C_{2}>0$ , depending only on *r*, such that if $n\geq C_{1}{\left(d\;\text{log}\;p\right)}^{6}\{d\;\text{log}\;p+\;\text{log}\;(1/\varepsilon )\}$ , and

$$\begin{array}{c} C_{2}\text{min}[{\left\{\frac{d\;\text{log}\;(p\vee n)+\;\text{log}\;(1/\varepsilon )}{n}\right\}}^{1/4},\sqrt{\frac{d\;\text{log}\;(p\vee n)+\;\text{log}\;(1/\varepsilon )}{n_{L}}}]\\ \;\leq \frac{{\left(\nu_{\text{min}}^{*}\right)}^{2}}{4d}, \end{array}$$

then the sequence of outputs ${\left({\hat{S}}^{\left(T\right)}\right)}_{T\geq 1}$ of Algorithm 1 with inputs *K* = 2, *p*, $s_{0}\leq d\leq \text{min}(p,n-K),\;l\geq s_{0},\;{\left(X_{i},Y_{i}\right)}_{i\in [n]}$ , *A*, *B* and base procedure $\psi^{\left(M,T\right)}$ satisfies $\underset{T\to \infty }{\text{liminf}}P(S_{0}\subseteq {\hat{S}}^{\left(T\right)})\geq 1-\varepsilon -pe^{-A{\left(\nu_{\text{min}}^{*}\right)}^{4}/(50p^{2}{\left(\nu_{\text{max}}^{*}\right)}^{4})}.$

Corollary 7 reveals in particular that, treating $\nu_{\text{max}}^{*}$ and $\nu_{\text{min}}^{*}$ as constants and under the stated sample size conditions, we again recover all of the signal coordinates in the top *s*_0_ output entries, provided that *A* is large by comparison with *p*^2^. Thus, in this sense, we can achieve a similar guarantee to that provided by Corollary 4, though the number of groups of projections required for a high probability guarantee in Corollary 7 may be significantly larger in settings where the ratio $\nu_{{\text{max}}^{*}}/\nu_{{\text{min}}^{*}}$ is large.

## 4 Numerical Studies

Throughout this section, unless otherwise stated, data ${\left(X_{i},Y_{i},Y_{i}^{*}\right)}_{i\in [n]}$ are sampled from an equal-probability normal mixture as follows: $P(Y_{i}^{*}=k)=1/K$ for $k\in [K],\;P(Y_{i}=Y_{i}^{*})=1-P(Y_{i}=0)=\gamma$ and $X_{i}|Y_{i}^{*}∼N_{p}(\mu_{Y_{i}^{*}},\Sigma_{w})$ . The cluster means ${\left(\mu_{k}\right)}_{k\in [K]}$ are chosen to be *s*_0_-sparse and we define the signal-to-noise ratio of the problem to be^3^ $\text{SNR}:=\frac{{\text{min}}_{k,k\prime \in [K],k\neq k\prime }\|\mu_{k}-\mu_{k\prime }\|}{\sqrt{\text{tr}(\Sigma_{w})/p}}.$ In our numerical studies, we slightly modify Algorithm 3 so that instead of randomly initializing the cluster means and the covariance matrix, we use the output of hierarchical clustering to initialize the EM algorithm as implemented in the mclust R package (Fraley and Raftery 1998). This allow us to run Algorithm 3 with *M* = 1.

### 4.1 Comparison with Existing Methods

Our goal here is to compare the empirical performance of the Sharp-SSL algorithm in high-dimensional clustering tasks with several existing approaches. We apply the Sharp-SSL algorithm using the EM algorithm of Algorithm 3 as a base procedure, with input parameters *A* = 150, *B* = 75, $d=l=s_{0}$ (choice of tuning parameters is discussed in Section S4.1), and our final estimated cluster labels are then obtained as described there.

We compare the Sharp-SSL algorithm with five alternative high-dimensional clustering methods: spectral clustering (e.g., von Luxburg 2007), the $l_{1}$ -penalized approach of Witten and Tibshirani (2010) and the RPEClus algorithm of Anderlucci, Fortunato, and Montanari (2022) as well as a pair of methods that, like Sharp-SSL, apply dimension reduction prior to a low-dimensional clustering algorithm.

In more detail, the spectral clustering approach first constructs a *J*-nearest neighbor graph adjacency matrix $A={\left(A_{i,i\prime }\right)}_{i,i\prime \in [n]}\in {\left\{0,1\right\}}^{n\times n}$ , where $A_{i,i\prime }:=1$ if either *X_i_* is one of the *J* = 10 nearest neighbors of $X_{i\prime }$ in Euclidean distance or vice versa, and $A_{i,i\prime }:=0$ otherwise. It then computes an *n* × *K* matrix of eigenvectors associated with the *K* smallest nonzero eigenvalues of the Laplacian matrix L:=D−A , where $D\in R^{n\times n}$ is a diagonal matrix with diagonal entries $D_{i,i}:=\sum_{i\prime \in [n]}A_{i,i\prime }$ . The final step is to apply the *K*-means clustering algorithm (Lloyd 1982), as implemented in the kmeans base R function with 100 random initializations, to the rows of *L* with the oracle choice of *K*.

The Witten and Tibshirani (2010) method, which is implemented in the sparcl R package, determines the estimated cluster memberships by maximizing a coordinatewise-weighted between-cluster sum of squares criterion, subject to an $l_{1}$ constraint on the weights. A permutation approach is used to select the $l_{1}$ tuning parameter.

In the RPEClus algorithm of Anderlucci, Fortunato, and Montanari (2022), we generate *B* random orthogonal projections and incorporate the *d*-dimensional projected data as covariates for a linear regression with the orthogonal complement of the projected data as the response. We then use the Bayesian Information Criteria (BIC) from both an application of the EM algorithm to the projected data and the aforementioned regression to identify good projections, and aggregate using the consensus clustering technique of Dimitriadou, Weingessel, and Hornik (2002) over the best $B^{*}$ projections chosen according to the sum of the BIC scores. Following the recommendation of Anderlucci, Fortunato, and Montanari (2022), we took *B* = 1000 and $B^{*}=100$ as well as *d* = *s*_0_. It turned out that this approach had a misclustering rate almost identical to that of a random guess, primarily because it did not leverage the sparsity of the signal. We therefore modified this method by generating random axis-aligned projections instead of orthogonal ones, and report this version in our comparison.

The first of the two-stage approaches applies principal component analysis (PCA) to project the data into the oracle choice of *K* – 1 dimensions (the dimension of the space spanned by the *K* cluster means); the second uses sparse principal component analysis (SPCA), as implemented in the SPCAvRP algorithm (Gataric, Wang, and Samworth 2020) with the default choices of *A* = 600 groups of *B* = 200 random projections in each group, and the oracle choices to project into *d* = *s*_0_ dimensions and return *K* – 1 eigenvectors having sparsity $l=s_{0}$ . Thereafter, both algorithms apply *K*-means to the projected data as above. We also explored the option of replacing the *K*-means steps in these latter algorithms with the EM algorithm, but observed very little difference, so do not report these results here.

**Fig. 1:**
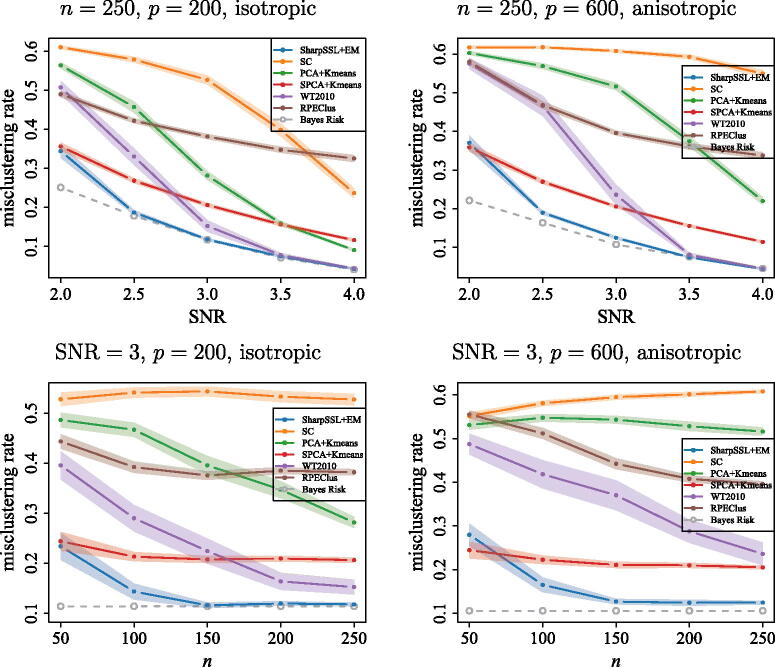
Average misclustering rate over 100 repetitions using Sharp-SSL followed by the EM algorithm, as well as using the other methods from Section 4.1. Data are generated from the normal mixture distribution described at the beginning of Section 4 with *K* = 3 and *p* = 200 (left) as well as *p* = 600 (right). The three cluster means are given by $\mu_{1}=a(1,1,0,0_{p-3}),\;\mu_{2}=a(-1,0,1,0_{p-3})$ and $\mu_{3}=a(0,-1,-1,0_{p-3})$ , where the scale *a* is chosen such that their pairwise distances are all equal to SNR . For isotropic settings (left), $\Sigma_{w}=I_{p}$ ; for anisotropic settings (right), $\Sigma_{w}=V\Lambda V^{⊤}$ , where $\Lambda \in R^{p\times p}$ is diagonal with independent Unif[0,2] diagonal entries and *V* is independent of Λ, and sampled from the Haar measure on $O^{p\times p}$ . The Bayes risk is shown as the gray dashed line. In the top panels, *n* = 250 and the SNR varies; in the bottom panels, SNR=3 and *n* varies. The shaded regions represent interpolated 95% confidence intervals at each of the points.

Given true labels $y_{1},\ldots ,y_{n}\in [K]$ and estimated labels ${\hat{y}}_{1},\ldots ,{\hat{y}}_{n}\in [K]$ from a clustering algorithm, we measure the performance of the algorithm via its *misclustering rate*, defined as^4^

$$L(\{y_{1},\ldots ,y_{n}\},\{{\hat{y}}_{1},\ldots ,{\hat{y}}_{n}\}):={\text{min}}_{\pi \in S_{K}}\frac{1}{n}\sum_{i=1}^{n}1_{\left\{\pi ({\hat{y}}_{i})\neq y_{i}\right\}},$$

where *S_K_* is the group of all permutations of [K] . In particular, Figure 1 presents the average misclustering rates over 100 Monte Carlo repetitions of the different high-dimensional clustering algorithms described above. Across two different dimensions p∈{200,600} , isotropic and anisotropic settings, and for different values of n∈{50,100,150,200,250} and SNR∈{2,2.5,3,3.5,4} , we see a consistent picture of the Sharp-SSL algorithm combined with EM producing the lowest misclustering rates, often by a large margin. Indeed, for all but the smallest sample sizes or values of SNR , the Sharp-SSL + EM algorithm nearly attains the Bayes risk in all of the problems considered here. Additional comparisons in misspecfied settings between the Sharp-SSL + EM method and alternative approaches are given in Section S4.2.

### 4.2 Effect of Observed Fraction on Misclustering Rate

One of the key attractions of our procedure is that it offers a unified framework to perform classification or clustering with an arbitrary fraction of labeled observations. In this subsection, we explore the performance of the algorithm as we vary the proportion of observed labels.

**Fig. 2:**
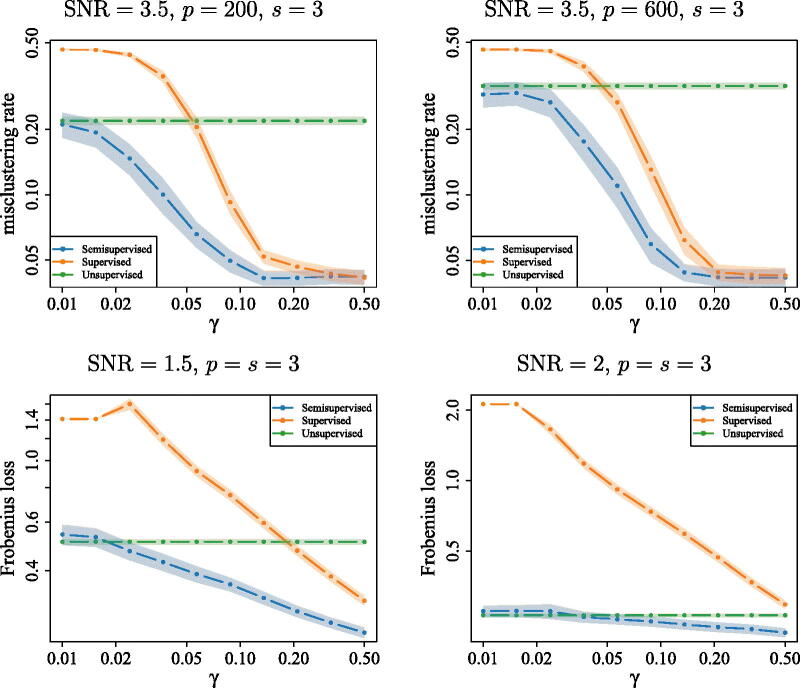
Effect of label fraction on performance of supervised, semi-supervised and unsupervised Sharp-SSL learning methods. Data are generated from the normal mixture distribution described at the beginning of Section 4 with *K* = 2 and $\Sigma_{w}=I_{p},\;\mu_{1}=-\mu_{2}=a{\left(1_{s},0_{p-s}\right)}^{⊤}\in R^{p}$ , where *a* is chosen such that $\|\mu_{1}-\mu_{2}\|=\text{SNR}$ . Bottom: average Frobenius loss of estimating the $(\mu_{1},\mu_{2})\in R^{p\times 2}$ over 100 repetitions via the semi-supervised approach (Algorithm 3), supervised approach (Algorithm 2) and unsupervised approach (Algorithm 3 without using the labels). Top: average misclustering rate over 100 repetitions from applying the above three methods as base algorithms in Algorithm 1. The shaded regions represent interpolated 95% confidence intervals at each of the points.

Recall that we have two different options for the way in which we implement the Sharp-SSL algorithm to estimate the set of signal coordinates: we can either use only the labeled data, as in the supervised learning approach of Algorithm 2, or we can try to leverage in addition the unlabeled data via the semi-supervised EM approach of Algorithm 3. In Figure 2 we compare the performance of these two methods with the baseline EM approach that ignores all labels in both high- and low-dimensional versions of the normal mixture distribution data generation mechanism described at the beginning of Section 4 as the proportion *γ* of observed labels varies. More precisely, for the semi-supervised and unsupervised algorithms, we adopt the same implementation of Sharp-SSL as described at the beginning of Section 4.1. The supervised algorithm is very similar, but applies Algorithm 2 in place of Algorithm 3 to select coordinates, and obtains final predicted labels by applying LDA again on the projected labeled data. In cases where the proportion of labeled data was so small that the convex hull of the projected labeled data was less than full-dimensional for every class, we forced Algorithm 2 to return a zero matrix (this only happened when *γ* was very small).

The top panels of Figure 2 present the results in high-dimensional settings with p∈{200,600} . Since the unsupervised approach has no access to the labels, it has constant misclustering rate. The performance of the semi-supervised approach is always at least as good as that of the unsupervised algorithm, and improves as *γ* increases. In other words, it effectively leverages the additional information provided by the class labels. When *γ* is very small, the supervised algorithm—which ignores the unlabeled data—is inaccurate, as it has very little data to work with. On the other hand, its performance also improves as *γ* increases, and once around 5% of our data are labeled, it outperforms the unsupervised algorithm. Further, it essentially matches the semi-supervised approach when about a third of the data are labeled. We truncate the plot at γ=1/2 to ensure that we have enough test data on which to compute the misclustering rate.

In the bottom panels of Figure 2, we explore the performance of the three algorithms above in two low-dimensional settings with different values of SNR, in order to provide further insight into the phenomena described in the previous paragraph. Here, we take *K* = 2 and report the average Frobenius norm loss $L(({\hat{\mu }}_{1},{\hat{\mu }}_{2}),(\mu_{1},\mu_{2})):=\text{min}\{\|({\hat{\mu }}_{1},{\hat{\mu }}_{2})-(\mu_{1},\mu_{2}){\|}_{F},\|({\hat{\mu }}_{2},{\hat{\mu }}_{1})-(\mu_{1},\mu_{2}){\|}_{F}\}$ of the estimated means, over 100 repetitions. If there are insufficient labeled data to run Algorithm 2, then we output ${\hat{\mu }}_{1}={\hat{\mu }}_{2}=0_{p}$ . We see that, already in these low-dimensional problems, a similar picture emerges: if the proportion of labeled data is small, then the unsupervised algorithm outperforms the supervised one, but this situation may be reversed when *γ* is larger. The semi-supervised algorithm is able to leverage both the unlabeled and labeled data to obtain the best of both worlds. These empirical observations agree with our theory from Section 3, in particular in the way in which Theorem 6 bounds the accuracy of mean estimation for the semi-supervised algorithm by a minimum of a term that does not depend on *γ* and one that decreases as *γ* increases. It appears that the switch in the minimum occurs around γ=0.02 in these examples.

### 4.3 Empirical Data Analysis

We apply Sharp-SSL, as well as several competing methods, to the gene expression dataset from Alon et al. (1999), which contains observations on 62 patients. A preprocessed version of the data can be downloaded from the R package “datamicroarray” (Ramey 2016), with a total of 2000 features (genes) measured on 40 patients with colon tumors and 22 without tumors. We first exclude 9 genes to remove perfect collinearity and then standardize each of the remaining *p* = 1991 columns of the dataset to have unit variance.

We apply the Sharp-SSL algorithm using EM (Algorithm 3) as the base procedure, with input parameters *A* = 150, *B* = 75, d=l=5 . In addition to our approach (Sharp-SSL + EM), we also compare the performance of spectral clustering (SC), the Witten and Tibshirani (2010) method (WT2010, as well as four two-stage methods (PCA + Kmeans, PCA + EM, SPCA + Kmeans, SPCA + EM), where we first reduce dimension of the data to a 5-dimensional subspace using either PCA or SPCA and then apply either the EM algorithm or *K*-means clustering on the low-dimensional data. For SPCA, we use the SPCAvRP algorithm (Gataric, Wang, and Samworth 2020) with inputs *A* = 600, *B* = 200 and d=l=5 . The true labels are hidden to all algorithms and are only used to evaluate the final misclustering rate.

**Fig. 3:**
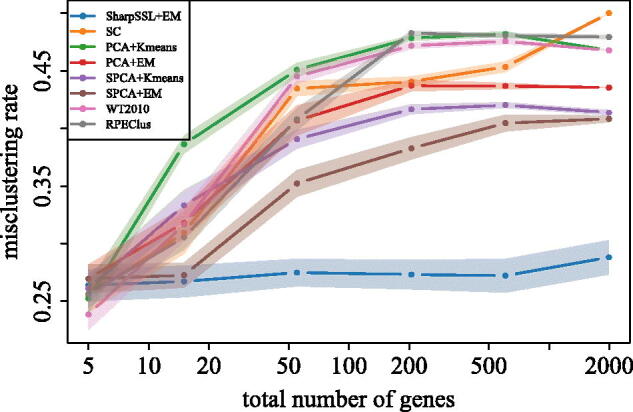
Average misclustering rate (over 100 repetitions for randomized algorithms) for the colon tumor data, using Sharp-SSL followed by the EM algorithm, as well as the other methods described in Section 4.3. The right-hand data points plot the average misclustering rate on the full dataset. The other points were obtained by applying each method to a subset of genes formed from the top five genes identified by Sharp-SSL together with randomly sampled genes. The shaded regions represent interpolated 95% confidence intervals at each of the points.

Over 100 Monte Carlo repetitions of the randomized algorithms, the Sharp-SSL + EM method had an average misclustering rate of 28.8%, whereas all other competitors had a misclustering rate above 40%, as can be seen from the right-hand data points in Figure 3. To investigate this performance further, we applied each method to a subset of the features. These were constructed from the top l=5 genes identified through Sharp-SSL, together with m=0,10,50,200 , and 600 randomly chosen genes from the remaining 1986. The results are presented as the other data points in Figure 3. We see that the improved performance of the Sharp-SSL + EM method relative to the other methods persists, even when only a small number of potentially non-discriminative covariates are present. When *m* = 0, Sharp-SSL + EM has a slight disadvantage as other algorithms benefit from the ensemble effect of combining two different learning methods; nevertheless it remains competitive. This reinforces the point that the primary contribution of the Sharp-SSL algorithm is to identify signal coordinates that are helpful for semi-supervised learning, and once this has been accomplished, a variety of low-dimensional procedures are available to the practitioner.

## 5 Discussion

The main contribution of this work is to propose the Sharp-SSL method for high-dimensional semi-supervising learning based on a careful aggregation of variables selected by running a low-dimensional algorithm on axis-aligned random projections of the data. An attraction of our framework is the way in which it can be combined with different base learning algorithms according to the proportion of labeled data and the desired characteristics of the low-dimensional learning algorithm. Our theory ensures that when our base procedure estimates our variable importance scores sufficiently well, the Sharp-SSL algorithm is able to recover the signal coordinates with high probability, provided we aggregate over sufficiently many random projections. Moreover, our numerical results on both simulated and real data illustrate that our methodology performs favorably in comparison with several state-of-the-art methods. In future work, one could study modifications of the Sharp-SSL algorithm presented in Algorithm 1 that might be applicable to other high-dimensional problems, such as sparse PCA or sparse graphical models. In a theoretical direction, it would be of interest to understand the performance of the Sharp-SSL algorithm in more general settings, for instance to study its robustness to outliers or heavy-tailed distributions.

## Supplementary Material

suppl_data__2_.zip

sharpSSL_supp.pdf

acc-form-2021.pdf

## Notes

1 It is convenient to use the term “classifier” here, even though some or all of the labels may be unobserved.

2 Even though $\Sigma_{w}^{-1}\Sigma_{b}$ is not guaranteed to be symmetric, it is similar (i.e., conjugate) to the symmetric matrix $\Sigma_{w}^{-1/2}\Sigma_{b}\Sigma_{w}^{-1/2}$ , so has real eigenvalues and eigenvectors.

3 In some of our simulations, $\Sigma_{w}$ was generated randomly for convenience. In such settings, we replaced $\text{tr}(\Sigma_{w})/p$ in the denominator of the SNR definition with $E\{\text{tr}(\Sigma_{w})\}/p$ .

4 Here, the minimum over permutations is taken because it is only the cluster groupings, and not the labels themselves, that are important.

## Supplementary Materials

The supplementary materials contain proofs and extensions of the theoretical results and additional numerical simulation experiments.

## References

[CIT0001] Abbe, E., Fan, J., and Wang, K. (2022), “An $l_{p}$ Theory of PCA and Spectral Clustering,” *The Annals of Statistics*, 50, 2359–2385.

[CIT0002] Ahfock, D. C., Astle, W. J., and Richardson, S. (2021), “Statistical Properties of Sketching Algorithms,” *Biometrika*, 108, 283–297. DOI: 10.1093/biomet/asaa062. 35125502 PMC7612324

[CIT0003] Akcay, S., Atapour-Abarghouei, A., and Breckon, T. P. (2019), “Ganomaly: Semi-Supervised Anomaly Detection via Adversarial Training,” in *14th Asian Conference on Computer Vision, Revised Selected Papers, Part* *III 14*, pp. 622–637, Springer.

[CIT0004] Alon, U., Barkai, N., Notterman, D. A., Gish, K., Ybarra, S., Mack, D., and Levine, A. J. (1999), “Broad Patterns of Gene Expression Revealed by Clustering Analysis of Tumor and Normal Colon Tissues Probed by Oligonucleotide Arrays,” *Proceedings of the National Academy of Sciences of the United States of America*, 96, 6745–6750. DOI: 10.1073/pnas.96.12.6745. 10359783 PMC21986

[CIT0005] Anderlucci, L., Fortunato, F., and Montanari, A. (2022), “High-Dimensional Clustering via Random Projections,” *Journal of Classification*, 39, 191–216. DOI: 10.1007/s00357-021-09403-7.

[CIT0006] Anderson, T. W. (2003), *An Introduction to Multivariate Statistical Analysis*, Wiley Series in Probability and Statistics, Hoboken, NJ: Wiley.

[CIT0007] Azizyan, M., Singh, A., and Wasserman, L. (2013), “Minimax Theory for High-Dimensional Gaussian Mixtures with Sparse Mean Separation,” in *Advances in Neural Information Processing Systems*, pp. 2139–2147.

[CIT0008] —– (2015), “Efficient Sparse Clustering of High-Dimensional Non-spherical Gaussian Mixtures,” in *Proceedings of the 18th International Conference on Artificial Intelligence and Statistics*, pp. 37–45.

[CIT0009] Balabdaoui, F., and Doss, C. R. (2018), “Inference for a Two-Component Mixture of Symmetric Distributions Under Log-Concavity,” *Bernoulli*, 24, 1053–1071. DOI: 10.3150/16-BEJ864.

[CIT0010] Balakrishnan, S., Wainwright, M. J., and Yu, B. (2017), “Statistical Guarantees for the EM Algorithm: From Population to Sample-based Analysis,” *The Annals of Statistics*, 45, 77–120. DOI: 10.1214/16-AOS1435.

[CIT0011] Bingham, E., and Mannila, H. (2001), “Random Projection in Dimensionality Reduction: Applications to Image and Text Data,” in *Proceedings of the Seventh ACM SIGKDD International Conference on Knowledge Discovery and Data Mining*, pp. 245–250.

[CIT0012] Butler, A., Hoffman, P., Smibert, P., Papalexi, E., and Satija, R. (2018), “Integrating Single-Cell Transcriptomic Data Across Different Conditions, Technologies, and Species,” *Nature Biotechnology*, 36, 411–420. DOI: 10.1038/nbt.4096. PMC6700744 29608179

[CIT0013] Cai, T. T., and Liu, W. (2011), “A Direct Estimation Approach to Sparse Linear Discriminant Analysis,” *Journal of the American Statistical Association*, 106, 1566–1577. DOI: 10.1198/jasa.2011.tm11199.

[CIT0014] Cai, T. T., and Zhang, L. (2019), “High Dimensional Linear Discriminant Analysis: Optimality, Adaptive Algorithm and Missing Data,” *Journal of the Royal Statistical Society*, Series B, 81, 675–705. DOI: 10.1111/rssb.12326.

[CIT0015] Cannings, T. I. (2021), “Random Projections: Data Perturbation for Classification Problems,” *Wiley Interdisciplinary Reviews: Computational Statistics*, 13, e1499.

[CIT0016] Cannings, T. I., Berrett, T. B., and Samworth, R. J. (2020), “Local Nearest Neighbour Classification with Applications to Semi-Supervised Learning,” *The Annals of Statistics*, 48, 1789–1814. DOI: 10.1214/19-AOS1868.

[CIT0017] Cannings, T. I., and Samworth, R. J. (2017), “Random-Projection Ensemble Classification,” *Journal of the Royal Statistical Society*, Series B, 79, 959–1035. DOI: 10.1111/rssb.12228.

[CIT0018] Chakrabortty, A., and Cai, T. (2018), “Efficient and Adaptive Linear Regression in Semi-Supervised Settings,” *The Annals of Statistics*, 46, 1541–1572. DOI: 10.1214/17-AOS1594.

[CIT0019] Chapelle, O., Schölkopf, B., and Zien, A. (eds.) (2006), *Semi-Supervised Learning*, Cambridge, MA: The MIT Press.

[CIT0020] Cheplygina, V., de Bruijne, M., and Pluim, J. P. (2019), “Not-So-Supervised: A Survey of Semi-Supervised, Multi-Instance, and Transfer Learning in Medical Image Analysis,” *Medical Image Analysis*, 54, 280–296. DOI: 10.1016/j.media.2019.03.009. 30959445

[CIT0021] Cule, M., Samworth, R., and Stewart, M. (2010), “Maximum Likelihood Estimation of a Multi-Dimensional Log-Concave Density,” *Journal of the Royal Statistical Society*, Series B, 72, 545–607. DOI: 10.1111/j.1467-9868.2010.00753.x.

[CIT0022] Dasgupta, S. (1999), “Learning Mixtures of Gaussians,” in *The 40th Annual Symposium on Foundations of Computer Science*, pp. 634–644.

[CIT0023] Dasgupta, S. and Gupta, A. (2003), “An elementary proof of a theorem of Johnson and Lindenstrauss,” *Random Structures & Algorithms*, 22, 60–65. DOI: 10.1002/rsa.10073.

[CIT0024] Daskalakis, C., Tzamos, C., and Zampetakis, M. (2017), “Ten Steps of EM Suffice for Mixtures of Two Gaussians,” in *Conference on Learning Theory*, PMLR, pp. 704–710.

[CIT0025] de Souto, M. C., Costa, I. G., de Araujo, D. S., Ludermir, T. B., and Schliep, A. (2008), “Clustering Cancer Gene Expression Data: A Comparative Study,” *BMC Bioinformatics*, 9, 497. DOI: 10.1186/1471-2105-9-497. 19038021 PMC2632677

[CIT0026] Devroye, L., Györfi, L., and Lugosi, G. (2013), *A Probabilistic Theory of Pattern Recognition* (Vol. 31), New York: Springer.

[CIT0027] Dimitriadou, E., Weingessel, A., and Hornik, K. (2002), “A Combination Scheme for Fuzzy Clustering,” *International Journal of Pattern Recognition and Artificial Intelligence*, 16, 901–912. DOI: 10.1142/S0218001402002052.

[CIT0028] Dobriban, E., and Liu, S. (2019), “Asymptotics for Sketching in Least Squares Regression,” in *Advances in Neural Information Processing Systems*, pp. 3675–3685.

[CIT0029] Doss, N., Wu, Y., Yang, P., and Zhou, H. H. (2023), “Optimal Estimation of High-Dimensional Gaussian Mixtures,” *The Annals of Statistics*, 51, 62–95. DOI: 10.1214/22-AOS2207.

[CIT0030] Durrant, R. J., and Kabán, A. (2015), “Random Projections as Regularizers: Learning a Linear Discriminant From Fewer Observations than Dimensions,” *Machine Learning*, 99, 257–286. DOI: 10.1007/s10994-014-5466-8.

[CIT0031] Dwivedi, R., Ho, N., Khamaru, K., Wainwright, M., Jordan, M., and Yu, B. (2020a), “Sharp Analysis of Expectation-Maximization for Weakly Identifiable Models,” in *International Conference on Artificial Intelligence and Statistics*, PMLR, pp. 1866–1876.

[CIT0032] Dwivedi, R., Ho, N., Khamaru, K., Wainwright, M. J., Jordan, M. I., and Yu, B. (2020b), “Singularity, Misspecification and the Convergence Rate of EM,” *The Annals of Statistics*, 48, 3161–3182. DOI: 10.1214/19-AOS1924.

[CIT0033] Eisen, M. B., Spellman, P. T., Brown, P. O., and Botstein, D. (1998), “Cluster Analysis and Display of Genome-Wide Expression Patterns,” *Proceedings of the National Academy of Sciences of the United States of America*, 95, 14863–14868. DOI: 10.1073/pnas.95.25.14863. 9843981 PMC24541

[CIT0034] Fern, X. Z., and Brodley, C. E. (2003), “Random Projection for High Dimensional Data Clustering: A Cluster Ensemble Approach,” in *Proceedings of the 20th International Conference on Machine Learning*, pp. 186–193.

[CIT0035] Fraley, C., and Raftery, A. (1998), “MCLUST: Software for Model-based Cluster and Discriminant Analysis,” Technical Report, 342, 1312.

[CIT0036] Gataric, M., Wang, T., and Samworth, R. J. (2020), “Sparse Principal Component Analysis via Axis-Aligned Random Projections.” *Journal of the Royal Statistical Society*, Series B, 82, 329–359. DOI: 10.1111/rssb.12360.

[CIT0037] Han, S., and Boutin, M. (2015), “The Hidden Structure of Image Datasets,” in *2015 IEEE International Conference on Image Processing (ICIP),* IEEE, pp. 1095–1099.

[CIT0038] Hastie, T., Tibshirani, R., and Friedman, J. H. (2009), *The Elements of Statistical Learning: Data Mining, Inference, and Prediction* (Vol. 2), New York: Springer.

[CIT0039] Ho, N., Khamaru, K., Dwivedi, R., Wainwright, M. J., Jordan, M. I., and Yu, B. (2020), “Instability, Computational Efficiency and Statistical Accuracy,” arXiv preprint arXiv:2005.11411.

[CIT0040] Jain, A. K., and Flynn, P. J. (1996), “Image Segmentation Using Clustering,” in *Advances in Image Understanding: A Festschrift for Azriel Rosenfeld*, pp. 65–83 Piscataway, NJ: IEEE Press.

[CIT0041] Jin, J., and Wang, W. (2016), “Influential Features PCA for High Dimensional Clustering,” *The Annals of Statistics*, 44, 2323–2359. DOI: 10.1214/15-AOS1423.

[CIT0042] Johnson, W. B., and Lindenstrauss, J. (1984), “Extensions of Lipschitz Maps into a Hilbert Space,” *Contemporary Mathematics*, 26, 189–206.

[CIT0043] Kaufman, L., and Rousseeuw, P. J. (2009), *Finding Groups in Data: An Introduction to Cluster Analysis* (Vol. 344), Hoboken, NJ: Wiley.

[CIT0044] Liang, P. (2005), “Semi-Supervised Learning for Natural Language,” Ph.D. Thesis, Massachusetts Institute of Technology.

[CIT0045] Lloyd, S. (1982), “Least Squares Quantization in PCM,” *IEEE Transactions on Information Theory*, 28, 129–137. DOI: 10.1109/TIT.1982.1056489.

[CIT0046] Löffler, M., Wein, A. S., and Bandeira, A. S. (2022), “Computationally Efficient Sparse Clustering,” *Information and Inference: A Journal of the IMA*, 11, 1255–1286. DOI: 10.1093/imaiai/iaac019.

[CIT0047] Löffler, M., Zhang, A. Y., and Zhou, H. H. (2021), “Optimality of Spectral Clustering in the Gaussian Mixture Model,” *The Annals of Statistics*, 49, 2506–2530. DOI: 10.1214/20-AOS2044.

[CIT0048] Lopes, M., Jacob, L., and Wainwright, M. J. (2011), “A More Powerful Two-Sample Test in High Dimensions Using Random Projection,” in *Advances in Neural Information Processing Systems*, pp. 1206–1214.

[CIT0049] Lu, Y., and Zhou, H. H. (2016), “Statistical and Computational Guarantees of Lloyd’s Algorithm and its Variants,” arXiv preprint arXiv:1612.02099.

[CIT0050] Mai, Q., Zou, H., and Yuan, M. (2012), “A Direct Approach to Sparse Discriminant Analysis in Ultra-High Dimensions,” *Biometrika*, 99, 29–42. DOI: 10.1093/biomet/asr066.

[CIT0051] Marzetta, T. L., Tucci, G. H., and Simon, S. H. (2011), “A Random Matrix-Theoretic Approach to Handling Singular Covariance Estimates,” *IEEE Transactions on Information Theory*, 57, 6256–6271. DOI: 10.1109/TIT.2011.2162175.

[CIT0052] Minsker, S., Ndaoud, M., and Shen, Y. (2021), “Minimax Supervised Clustering in the Anisotropic Gaussian Mixture Model: A New Take on Robust Interpolation,” arXiv preprint arXiv:2111.07041.

[CIT0053] Ndaoud, M. (2022), “Sharp Optimal Recovery in the Two Component Gaussian Mixture Model,” *The Annals of Statistics*, 50, 2096–2126. DOI: 10.1214/22-AOS2178.

[CIT0054] Oymak, S., and Gulcu, T. C. (2020), “Statistical and Algorithmic Insights for Semi-Supervised Learning with Self-Training,” Preprint, arxiv:2006.11006.

[CIT0055] Ramey, J. A. (2016), Datamicroarray: Collection of Data Sets for Classification, R Package, available at https://rdrr.io/github/ramhiser/datamicroarray/.

[CIT0056] Reeve, H. W., Kabán, A., and Bootkrajang, J. (2022), “Heterogeneous Sets in Dimensionality Reduction and Ensemble Learning,” *Machine Learning*, 113, 1683–1704. DOI: 10.1007/s10994-022-06254-0.

[CIT0057] Rodriguez, M. Z., Comin, C. H., Casanova, D., Bruno, O. M., Amancio, D. R., Costa, L. d. F., and Rodrigues, F. A. (2019), “Clustering Algorithms: A Comparative Approach,” *PloS One*, 14, e0210236. DOI: 10.1371/journal.pone.0210236. 30645617 PMC6333366

[CIT0058] Samworth, R. J. (2018), “Recent Progress in Log-Concave Density Estimation,” *Statistical Science*, 33, 493–509. DOI: 10.1214/18-STS666.

[CIT0059] Slawski, M. (2018), “On Principal Components Regression, Random Projections, and Column Subsampling,” *Electronic Journal of Statistics*, 12, 3673–3712. DOI: 10.1214/18-EJS1486.

[CIT0060] Thanei, G.-A., Heinze, C., and Meinshausen, N. (2017), “Random Projections for Large-Scale Regression,” in *Big and Complex Data Analysis*, ed. S. Ejaz Ahmed, pp. 51–68, Cham: Springer.

[CIT0061] Turian, J., Ratinov, L., and Bengio, Y. (2010), “Word Representations: A Simple and General Method for Semi-Supervised Learning,” in *Proceedings of the 48th Annual Meeting of the Association for Computational Linguistics*, pp. 384–394.

[CIT0062] Van Engelen, J. E., and Hoos, H. H. (2020), “A Survey on Semi-Supervised Learning,” *Machine Learning*, 109, 373–440. DOI: 10.1007/s10994-019-05855-6.

[CIT0063] Verzelen, N., and Arias-Castro, E. (2017), “Detection and Feature Selection in Sparse Mixture Models,” *The Annals of Statistics*, 45, 1920–1950. DOI: 10.1214/16-AOS1513.

[CIT0064] von Luxburg, U. (2007), “A Tutorial on Spectral Clustering,” *Statistics and Computing*, 17, 395–416. DOI: 10.1007/s11222-007-9033-z.

[CIT0065] Walther, G. (2002), “Detecting the Presence of Mixing with Multiscale Maximum Likelihood,” *Journal of the American Statistical Association*, 97, 508–513. DOI: 10.1198/016214502760047032.

[CIT0066] Wang, D., Lin, J., Cui, P., Jia, Q., Wang, Z., Fang, Y., Yu, Q., Zhou, J., Yang, S., and Qi, Y. (2019), “A Semi-Supervised Graph Attentive Network for Financial Fraud Detection,” in *2019 IEEE International Conference on Data Mining (ICDM),* IEEE, pp. 598–607.

[CIT0067] Wasserman, L., Azizyan, M., and Singh, A. (2014), “Feature Selection for High-Dimensional Clustering,” Preprint, arxiv:1406.2240.

[CIT0068] Witten, D. M., and Tibshirani, R. (2010), “A Framework for Feature Selection in Clustering,” *Journal of the American Statistical Association*, 105, 713–726. DOI: 10.1198/jasa.2010.tm09415. 20811510 PMC2930825

[CIT0069] —– (2011), “Penalized Classification Using Fisher’s Linear Discriminant,” *Journal of the Royal Statistical Society*, Series B, 73, 753–772. 10.1111/j.1467-9868.2011.00783.x PMC3272679 22323898

[CIT0070] Wu, Y., and Zhou, H. H. (2022), “Randomly Initialised EM Algorithm for Two-Component Gaussian Mixture Achieves Near Optimality in $O(\sqrt{n})$ Iterations,” *Mathematical Statistics and Learning*, 4, 143–220.

[CIT0071] Xu, D., and Tian, Y. (2015), “A Comprehensive Survey of Clustering Algorithms,” *Annals of Data Science*, 2, 165–193. DOI: 10.1007/s40745-015-0040-1.

[CIT0072] Xu, R., and Wunsch, D. (2005), “Survey of Clustering Algorithms,” *IEEE Transactions on Neural Networks*, 16, 645–678. DOI: 10.1109/TNN.2005.845141. 15940994

[CIT0073] Yan, B., Yin, M., and Sarkar, P. (2017), “Convergence of gradient EM on multi-component mixture of Gaussians,” in *Advances in Neural Information Processing Systems*, pp. 6956–6966.

[CIT0074] Yang, F., Liu, S., Dobriban, E., and Woodruff, D. P. (2021), “How To Reduce Dimension with PCA and Random Projections?” *IEEE Transactions on Information Theory*, 67, 8154–8189. DOI: 10.1109/tit.2021.3112821. 35695837 PMC9173709

[CIT0075] Yellamraju, T., and Boutin, M. (2018), “Clusterability and Clustering of Images and Other “Real” High-Dimensional Data,” *IEEE Transactions on Image Processing*, 27, 1927–1938. DOI: 10.1109/TIP.2017.2789327. 33156781

[CIT0076] Zhang, A., Brown, L. D., and Cai, T. T. (2019), “Semi-Supervised Inference: General Theory and Estimation of Means,” *The Annals of Statistics*, 47, 2538–2566. DOI: 10.1214/18-AOS1756.

[CIT0077] Zhang, A. Y., and Zhou, H. H. (2022), “Leave-One-Out Singular Subspace Perturbation Analysis for Spectral Clustering,” arXiv preprint arXiv:2205.14855.

[CIT0078] Zhu, X., and Goldberg, A. B. (2009), “Introduction to Semi-Supervised Learning,” in *Synthesis Lectures on Artificial Intelligence and Machine Learning*, eds. R. J. Brachman and T. Dietterich, pp. 1–130, Kentfield, CA: Morgan & Claypool Publishers. DOI: 10.2200/S00196ED1V01Y200906AIM006.

[CIT0079] Zhu, X. J. (2005), “Semi-Supervised Learning Literature Survey,” Technical Report, University of Wisconsin-Madison Department of Computer Sciences.

