# Supplementary material for: Sharp-SSL: Selective High-Dimensional Axis-Aligned Random Projections for Semi-Supervised Learning
Source: J Am Stat Assoc. 2024 Apr 12;120(549):395–407. doi: 10.1080/01621459.2024.2340792 (PMC12012707; doi:10.1080/01621459.2024.2340792)
Supplement: sharpSSL_supp.pdf [file UASA_A_2340792_SM6405.pdf]

Sharp-SSL: Selective high-dimensional axis-aligned random projections for semi-supervised learning

Tengyao Wang*, Edgar Dobriban†, Milana Gataric‡
and Richard J. Samworth‡

*Department of Statistics, London School of Economics
†Department of Statistics and Data Science, University of Pennsylvania
‡Statistical Laboratory, University of Cambridge

April 4, 2024

In this supplementary material, we provide proofs of the results stated in the main text (Section S1), an extension of the main results in the supervised setting to unequal within class covariances (Section S2), as well as auxiliary results and their proofs (Section S3). In Section S4, we present additional simulation results that illustrate the empirical performance of the `Sharp-SSL` algorithm.

S1 Proofs of the main results

Supplementary notation. The elementwise product of two vectors a, b of the same size is denoted by $a \odot b$. Moreover, we denote the elementwise inverse of a vector a of non-zero elements by a^{-1} and elementwise square of a vector a by $a^{\odot 2}$. For $M = (M_{qr}) \in \mathbb{R}^{q\times q}$, we write $\overrightarrow{\mathrm{diag}}\, M := (m_{11}, \ldots, m_{qq})^\top \in \mathbb{R}^q$ for the vector of diagonal elements of M. For any set $S \subseteq \mathbb{R}^d$ and $d \leq |S|$, we write $\binom{S}{d} := \{A \subseteq S : |A| = d\}$.

S1.1 Proof of Proposition 1

The assumption that the convex hull of $\nu_1, \ldots, \nu_K$ is $(K-1)$-dimensional implies that Σ_{b} is of rank $K-1$. Define $A := \Sigma_{\mathrm{w}}^{-1/2}\Sigma_{\mathrm{b}}\Sigma_{\mathrm{w}}^{-1/2} \in \mathbb{R}^{p\times p}$, which has rank $K-1$. Given

The research of the first, third and last authors was supported by EPSRC grants EP/T02772X/1, EP/T017961/1, EP/P031447/1 and EP/N031938/1, as well as ERC Advanced Grant 101019498. The second author was supported in part by NSF award DMS 2046874 (CAREER).

$V \in \mathbb{O}^{p\times d}$, we can find $Q \in \mathbb{O}^{p\times d}$ with the same column span as that of $\Sigma_{\mathrm{w}}^{1/2}V$ and let $R := Q^\top \Sigma_{\mathrm{w}}^{1/2} V \in \mathbb{R}^{d\times d}$, so that R is invertible, and $\Sigma_{\mathrm{w}}^{1/2}V = QR$. We observe that

$$\mathrm{tr}\{(V^\top \Sigma_{\mathrm{w}} V)^{-1}(V^\top \Sigma_{\mathrm{b}} V)\} = \mathrm{tr}\{(R^\top R)^{-1}(R^\top Q^\top AQR)\} = \mathrm{tr}(Q^\top AQ).$$

Thus, $J(V;\Sigma_{\mathrm{b}},\Sigma_{\mathrm{w}})$ depends on V only through the column space of $\Sigma_{\mathrm{w}}^{1/2}V$. Moreover, $\mathrm{tr}(Q^\top AQ)$ is maximized when Q, or equivalently $\Sigma_{\mathrm{w}}^{1/2}V$, spans a d-dimensional space that contains the $(K-1)$-dimensional eigenspace corresponding to the non-zero eigenvalues of A. Note that if for some $v \in \mathbb{R}^p \setminus \{0\}$ and $\lambda \geq 0$, we have $Av = \lambda v$, then $\Sigma_{\mathrm{w}}^{-1}\Sigma_{\mathrm{b}}\Sigma_{\mathrm{w}}^{-1/2}v = \Sigma_{\mathrm{w}}^{-1/2}Av = \lambda\Sigma_{\mathrm{w}}^{-1/2}v$, so $\Sigma_{\mathrm{w}}^{-1/2}v$ is an eigenvector of $\Sigma_{\mathrm{w}}^{-1}\Sigma_{\mathrm{b}}$ with eigenvalue λ. Hence V maximizes $J(V;\Sigma_{\mathrm{b}},\Sigma_{\mathrm{w}})$ over $\mathbb{O}^{p\times d}$ if and only if V spans a d-dimensional space that contains the $(K-1)$-dimensional eigenspace corresponding to the $K-1$ non-zero eigenvalues of $\Sigma_{\mathrm{w}}^{-1}\Sigma_{\mathrm{b}}$. Finally, for any $v \in \mathbb{R}^p \setminus \{0\}$,

$$v^\top \Sigma_{\mathrm{w}}^{-1/2}\Sigma_{\mathrm{b}}\Sigma_{\mathrm{w}}^{-1/2}v = \sum_{k=1}^{K} \pi_k v^\top \Sigma_{\mathrm{w}}^{-1/2}(\nu_k - \nu)(\nu_k - \nu)^\top \Sigma_{\mathrm{w}}^{-1/2} v \neq 0$$

if and only if $v^\top \Sigma_{\mathrm{w}}^{-1/2}(\nu_k - \nu) \neq 0$ for some $k \in [K]$. Thus, the eigenspace corresponding to the non-zero eigenvalues of A is spanned by $\bigl(\Sigma_{\mathrm{w}}^{-1/2}(\nu_k - \nu) : k \in [K]\bigr)$, and so the eigenspace corresponding to non-zero eigenvalues of $\Sigma_{\mathrm{w}}^{-1}\Sigma_{\mathrm{b}}$ is spanned by $\bigl(\Sigma_{\mathrm{w}}^{-1}(\nu_k - \nu) : k \in [K]\bigr)$.

S1.2 Proof of Theorem 2

We write $S^{a,b} := \{j \in [p] : (P^{a,b,\top}P^{a,b})_{j,j} = 1\}$. For any $S = \{j_1,\ldots,j_d\}^\top \in \binom{[p]}{d}$, we identify the set S with the sequence $j_{i_1} < \ldots < j_{i_d}$ sorted in increasing order; and with a slight abuse of notation, we will use S to refer to either object, which will always be clear depending on the context. We define $P^S \in \mathcal{P}_d$ by $(P^S)_{\ell,j} := \mathbb{1}_{\{j=j_\ell\}}$, so that $P^{S,\top}P^S = \mathrm{diag}\bigl((\mathbb{1}_{\{j\in S\}})_{j\in[p]}\bigr)$. Define $w^S := P^S w$ and $\hat{w}^S := \psi\bigl((P^S X_i, Y_i)_{i\in[n]}\bigr) \in \mathbb{R}^d$. Note that $\hat{w}^{a,b} = \hat{w}^{S^{a,b}}$ in this notation, and we will similarly denote $w^{a,b} := w^{S^{a,b}}$ for simplicity. Moreover, for any S, we define $t^S = \sum_{j\in S} w_j^S$, $t^{a,b} = t^{S^{a,b}}$, $\hat{t}^S = \sum_{j\in S}\hat{w}_j^S$, $\hat{t}^{a,b} = \hat{t}^{S^{a,b}}$. Defining

$$\Omega = \left\{ \max_{S\in\binom{[p]}{d}} \bigl\|\hat{w}^S - w^S\bigr\|_1 < \frac{\gamma_{\min}}{4} \right\},$$

recalling that ψ is permutation-equivariant, we have $\mathbb{P}(\Omega) \geq 1 - \varepsilon$ by (6). We will work on the event Ω throughout the remainder of the proof, and recall that by definition, $\gamma_{\min} > 0$.

By the triangle inequality, for any $S, S' \in \binom{[p]}{d}$ such that $S \cap S_0$ is a proper subset of $S' \cap S_0$, we have on Ω that

$$\hat{t}^S - \hat{t}^{S'} = \hat{t}^S - t^S - (\hat{t}^{S'} - t^{S'}) - (t^{S'} - t^S) \tag{S1}$$
$$\leq \big\|\hat{w}^S - w^S\big\|_1 + \big\|\hat{w}^{S'} - w^{S'}\big\|_\infty - \Bigg(\sum_{j \in S' \cap S_0} w_j - \sum_{j \in S \cap S_0} w_j\Bigg) \leq \frac{\gamma_{\min}}{2} - \sum_{j \in (S' \setminus S) \cap S_0} w_j < 0.$$

Fix $a \in [A]$, and for any $\tilde{j} \in [p]$, define $q_{\tilde{j}} := \mathbb{P}\big(\tilde{j} \in S^{a,b^*(a)} \mid (X_i, Y_i)_{i \in [n]}\big)$. Now fix some $j \in S_0$ and $j' \in [p] \setminus S_0$. We claim that

$$(q_j - q_{j'})\mathbb{1}_\Omega \geq 0. \tag{S2}$$

To verify this claim, define for $\tilde{j} \in \{j, j'\}$ and $b \in [B]$ the sets

$$\mathcal{S}_{b,\tilde{j}} := \big\{(S^{a,1}, \ldots, S^{a,B}) : b^*(a) = b, \tilde{j} \in S^{a,b}\big\} \quad \text{and} \quad \mathcal{S}_b := \big\{(S^{a,1}, \ldots, S^{a,B}) : b^*(a) = b\big\}.$$

Let $f : \binom{[p]}{d} \to \binom{[p]}{d}$ be a map defined by

$$f(S) := \begin{cases} (S \setminus \{j'\}) \cup \{j\} & \text{if } j \notin S \text{ and } j' \in S \\ S & \text{otherwise.} \end{cases}$$

If $j \notin S^{a,b}$ and $j' \in S^{a,b}$, then $f(S^{a,b}) \cap S_0 = (S^{a,b} \cup \{j\}) \cap S_0 = (S^{a,b} \cap S_0) \cup \{j\}$, so $S^{a,b} \cap S_0$ is a proper subset of $f(S^{a,b}) \cap S_0$; on the other hand, if either $j \in S^{a,b}$ or $j, j' \notin S^{a,b}$, then $f(S^{a,b}) = S^{a,b}$. It follows by (S1) that on Ω we have

$$\hat{t}^{S^{a,b}} \leq \hat{t}^{f(S^{a,b})}. \tag{S3}$$

Now let $F : \mathcal{S}_{b,j'} \to \mathcal{S}_{b,j}$ be defined as

$$F(S^{a,1}, \ldots, S^{a,B}) := \big(S^{a,1}, \ldots, S^{a,b-1}, f(S^{a,b}), S^{a,b+1}, \ldots, S^{a,B}\big).$$

We claim that F is both well-defined and injective on Ω. For the first of these claims, we note that since $j' \in S^{a,b}$, we must have $j \in f(S^{a,b})$. Moreover, if $(S^{a,1}, \ldots, S^{a,B}) \in \mathcal{S}_{b,j'}$, then $b^*(a) = b$. But (S3) holds on Ω, so $\big(S^{a,1}, \ldots, S^{a,b-1}, f(S^{a,b}), S^{a,b+1}, \ldots, S^{a,B}\big) \in \mathcal{S}_{b,j}$.

Hence F is well-defined. For the second claim, suppose that $S_1, S_2 \in \binom{[p]}{d}$ are such that $j' \in S_1 \cap S_2$ and $f(S_1) = f(S_2)$. If $j \in S_1 \cap S_2$, then $S_1 = f(S_1) = f(S_2) = S_2$; if $j \in S_1$ but $j \notin S_2$, then $j \in f(S_2) \setminus f(S_1)$, a contradiction. Similarly, we cannot have $j \notin S_1$ but $j \in S_2$. Finally, if $j \in S_1^{\mathrm{c}} \cap S_2^{\mathrm{c}}$, then $S_1 = \bigl(f(S_1) \setminus \{j\}\bigr) \cup \{j'\} = \bigl(f(S_2) \setminus \{j\}\bigr) \cup \{j'\} = S_2$. We deduce that f is injective on $\{S : j' \in S\}$. Since $j' \in S^{a,b}$ for $(S^{a,1}, \ldots, S^{a,B}) \in \mathcal{S}_{b,j'}$, this establishes the injectivity of F. In particular, $|\mathcal{S}_{b,j'}| \leq |\mathcal{S}_{b,j}|$. Consequently, on Ω, we have for all $b \in [B]$ that

$$\begin{aligned}\mathbb{P}\bigl(j \in S^{a,b^*(a)} \mid (X_i, Y_i)_{i\in[n]}, b^*(a) = b\bigr) &= \frac{\mathbb{P}\bigl(j \in S^{a,b^*(a)}, b^*(a) = b \mid (X_i, Y_i)_{i\in[n]}\bigr)}{\mathbb{P}\bigl(b^*(a) = b \mid (X_i, Y_i)_{i\in[n]}\bigr)} = \frac{|\mathcal{S}_{b,j}|}{|\mathcal{S}_b|} \\ &\geq \frac{|\mathcal{S}_{b,j'}|}{|\mathcal{S}_b|} = \frac{\mathbb{P}\bigl(j' \in S^{a,b^*(a)}, b^*(a) = b \mid (X_i, Y_i)_{i\in[n]}\bigr)}{\mathbb{P}\bigl(b^*(a) = b \mid (X_i, Y_i)_{i\in[n]}\bigr)} \\ &= \mathbb{P}\bigl(j' \in S^{a,b^*(a)} \mid (X_i, Y_i)_{i\in[n]}, b^*(a) = b\bigr),\end{aligned}$$

which implies Claim (S2). We remark that one consequence of (S2) is that, since $d \geq s_0$, we have on Ω that

$$q_j \geq \frac{\sum_{\tilde{j}\in([p]\setminus S_0)\cup\{j\}} q_{\tilde{j}}}{p - s_0 + 1} = \frac{d - \sum_{\tilde{j}\in S_0\setminus\{j\}} q_{\tilde{j}}}{p - s_0 + 1} \geq \frac{d - s_0 + 1}{p - s_0 + 1} \geq \frac{1}{p}. \tag{S4}$$

Again fixing $j \in S_0$ and $j' \notin S_0$, we observe on $\Omega \cap \{j \in S^{a,b^*(a)}\}$ that

$$\begin{aligned}\frac{3}{4}\gamma_{\min} &\leq \bigl[P^{a,b^*(a),\top} w^{a,b^*(a)}\bigr]_j - \bigl\|\hat{w}^{a,b^*(a)} - w^{a,b^*(a)}\bigr\|_\infty \leq \bigl[P^{a,b^*(a),\top} \hat{w}^{a,b^*(a)}\bigr]_j \\ &\leq \bigl[P^{a,b^*(a),\top} w^{a,b^*(a)}\bigr]_j + \bigl\|\hat{w}^{a,b^*(a)} - w^{a,b^*(a)}\bigr\|_\infty \leq \frac{5}{4}\gamma_{\max},\end{aligned}$$

and similarly on $\Omega \cap \{j' \in S^{a,b^*(a)}\}$ that $\bigl|\bigl[P^{a,b^*(a),\top} \hat{w}^{a,b^*(a)}\bigr]_{j'}\bigr| \leq \gamma_{\min}/4$. Recall also that $\bigl[P^{a,b^*(a),\top} \hat{w}^{a,b^*(a)}\bigr]_{\tilde{j}} = 0$ for all $\tilde{j} \notin S^{a,b^*(a)}$. Combining the above bounds on the diagonal entries of $\hat{Q}^{a,b^*(a)}$ with (S2) and (S4), we have on Ω that

$$\begin{aligned}&\mathbb{E}\bigl(\bigl[P^{a,b^*(a),\top} \hat{w}^{a,b^*(a)}\bigr]_j - \bigl[P^{a,b^*(a),\top} \hat{w}^{a,b^*(a)}\bigr]_{j'} \mid (X_i, Y_i)_{i\in[n]}\bigr) \\ &\quad = \mathbb{E}\bigl(\bigl[P^{a,b^*(a),\top} \hat{w}^{a,b^*(a)}\bigr]_j \mathbb{1}_{\{j\in S^{a,b^*(a)}\}} - \bigl[P^{a,b^*(a),\top} \hat{w}^{a,b^*(a)}\bigr]_{j'} \mathbb{1}_{\{j'\in S^{a,b^*(a)}\}} \mid (X_i, Y_i)_{i\in[n]}\bigr) \\ &\quad \geq \frac{q_j \gamma_{\min}}{2} \geq \frac{\gamma_{\min}}{2p}.\end{aligned} \tag{S5}$$

Now, let a, j, j' be freely varying again. Since $\hat{w} = A^{-1}\sum_{a=1}^{A} P^{a,b^*(a),\top}\hat{w}^{a,b^*(a)}$, on Ω we have for any $j \in S_0$ and $j' \notin S_0$ that $\mathbb{E}\bigl(\hat{w}_j - \hat{w}_{j'} \mid (X_i, Y_i)_{i\in[n]}\bigr) \geq \gamma_{\min}/(2p)$ from (S5). Since $\ell \geq s_0$, we have by Hoeffding's inequality that on Ω,

$$
\begin{aligned}
\mathbb{P}\bigl(S_0 \not\subseteq \hat{S} \mid (X_i, Y_i)_{i\in[n]}\bigr) &\leq \mathbb{P}\Bigl(\min_{j\in S_0} \hat{w}_j \leq \max_{j'\notin S_0} \hat{w}_{j'} \Bigm| (X_i, Y_i)_{i\in[n]}\Bigr) \\
&\leq \sum_{j\in S_0} \mathbb{P}\biggl\{\hat{w}_j - \mathbb{E}(\hat{w}_j \mid (X_i, Y_i)_{i\in[n]}) \leq -\frac{\gamma_{\min}}{4p} \biggm| (X_i, Y_i)_{i\in[n]}\biggr\} \\
&\quad + \sum_{j\notin S_0} \mathbb{P}\biggl\{\hat{w}_j - \mathbb{E}(\hat{w}_j \mid (X_i, Y_i)_{i\in[n]}) \geq \frac{\gamma_{\min}}{4p} \biggm| (X_i, Y_i)_{i\in[n]}\biggr\} \\
&\leq p\exp\biggl\{-\frac{A}{2}\Bigl(\frac{\gamma_{\min}}{4p}\Bigr)^2 \Bigm/ \Bigl(\frac{5\gamma_{\max}}{4}\Bigr)^2\biggr\} \leq pe^{-A\gamma_{\min}^2/(50p^2\gamma_{\max}^2)},
\end{aligned}
$$

as desired.

S1.3 Proof of Theorem 3

The main ingredient of the proof of Theorem 3 is the following proposition, which controls the rate of convergence of the sample between- and within-class covariance matrices to their respective population versions in a classification problem.

Proposition S1. *Suppose that $(Z_1, Y_1), \ldots, (Z_n, Y_n)$ are independent and identically distributed data-label pairs, such that $\mathbb{P}(Y_1 = k) = \pi_k$ and $Z_1 \mid Y_1 = k \sim \mathcal{N}_d(\mu_k, \Gamma_{\mathrm{w}})$ for $k \in [K]$, where $\Gamma_{\mathrm{w}} \in \mathbb{S}_+^{d\times d}$. Write $\mu := \sum_{k\in[K]} \pi_k \mu_k$ and $\Gamma_{\mathrm{b}} := \sum_{k\in[K]} \pi_k(\mu_k - \mu)(\mu_k - \mu)^\top$ and let $\hat{\Gamma}_{\mathrm{w}}$ and $\hat{\Gamma}_{\mathrm{b}}$ be computed as in (3) applied to $(Z_1, Y_1), \ldots, (Z_n, Y_n)$. If $\|\mu_k - \mu\|_\infty \leq R_1$ for all $k \in [K]$ and $\|\overrightarrow{\mathrm{diag}}(\Gamma_{\mathrm{w}})\|_\infty \leq R_2$ for some $R_1, R_2 > 0$, then for every $\delta \in (0, 1/(8d)]$, we have with simultaneous probability at least $1 - \delta$ that*

$$
\|\overrightarrow{\mathrm{diag}}(\hat{\Gamma}_{\mathrm{b}}) - \overrightarrow{\mathrm{diag}}(\Gamma_{\mathrm{b}})\|_\infty \leq \frac{3R_2\{K + \log(8d/\delta)\}}{n} + R_1\bigl(3R_1 + 2\sqrt{R_2}\bigr)\sqrt{\frac{2\log(8d/\delta)}{n}}
$$

and

$$
\|\overrightarrow{\mathrm{diag}}(\hat{\Gamma}_{\mathrm{w}}) - \overrightarrow{\mathrm{diag}}(\Gamma_{\mathrm{w}})\|_\infty \leq \frac{2R_2\{K + \log(8d/\delta)\}}{n} + 2R_2\sqrt{\frac{\log(8d/\delta)}{n}}.
$$

Proof. For a vector v, we write $v^{\odot 2}$ for its entrywise squared vector. For $k \in [K]$, write $n_k := \sum_{i=1}^{n} \mathbb{1}_{\{Y_i = k\}}$ and $\tilde{\mu} := \sum_{k=1}^{K}(n_k/n)\mu_k$, define $\tilde{\mathcal{D}}_{\mathrm{b}}^{(1)} := \sum_{k=1}^{K}(n_k/n)(\mu_k - \mu)^{\odot 2}$ and

$\tilde{\mathcal{D}}_{\mathrm{b}}^{(2)} := \sum_{k=1}^{K}(n_k/n)(\mu_k - \tilde{\mu})^{\odot 2}$. We further denote $\mathcal{D}_{\mathrm{b}} := \overrightarrow{\mathrm{diag}}(\Gamma_{\mathrm{b}})$ and define $\mathcal{D}_{\mathrm{w}}, \hat{\mathcal{D}}_{\mathrm{b}}, \hat{\mathcal{D}}_{\mathrm{w}}$ similarly. We have

$$\|\hat{\mathcal{D}}_{\mathrm{b}} - \mathcal{D}_{\mathrm{b}}\|_\infty \leq \|\tilde{\mathcal{D}}_{\mathrm{b}}^{(1)} - \mathcal{D}_{\mathrm{b}}\|_\infty + \|\tilde{\mathcal{D}}_{\mathrm{b}}^{(2)} - \tilde{\mathcal{D}}_{\mathrm{b}}^{(1)}\|_\infty + \|\hat{\mathcal{D}}_{\mathrm{b}} - \tilde{\mathcal{D}}_{\mathrm{b}}^{(2)}\|_\infty. \tag{S6}$$

We will control the three terms on the right-hand side above separately. For the first term, since $n_1, \ldots, n_K$ are functions of $Y_1, \ldots, Y_n$, we have by McDiarmid's inequality (e.g. **?**, Theorem 6.2) that with probability at least $1 - \delta/4$,

$$\|\tilde{\mathcal{D}}_{\mathrm{b}}^{(1)} - \mathcal{D}_{\mathrm{b}}\|_\infty \leq R_1^2 \sum_{k=1}^{K} \left| \frac{n_k}{n} - \pi_k \right| \leq R_1^2 \sqrt{\frac{2\log(8/\delta)}{n}}. \tag{S7}$$

For the second term, we first apply McDiarmid's inequality again to see that with probability at least $1 - \delta/4$,

$$\|\tilde{\mu} - \mu\|_\infty \leq \sum_{k=1}^{K} \left| \frac{n_k}{n} - \pi_k \right| \|\mu_k - \mu\|_\infty \leq R_1 \sqrt{\frac{2\log(8/\delta)}{n}}.$$

Thus, we have with probability at least $1 - \delta/4$ that

$$\|\tilde{\mathcal{D}}_{\mathrm{b}}^{(2)} - \tilde{\mathcal{D}}_{\mathrm{b}}^{(1)}\|_\infty \leq \sum_{k=1}^{K} \frac{n_k}{n} \|\mu_k - \mu + \mu_k - \tilde{\mu}\|_\infty \|\mu - \tilde{\mu}\|_\infty \leq 2R_1^2 \sqrt{\frac{2\log(8/\delta)}{n}}. \tag{S8}$$

Finally, for the third term, we write $\hat{\mu}_k := n_k^{-1} \sum_{i:Y_i=k} Z_i$ and note that $V_k := n_k^{1/2}\hat{\mu}_k$ satisfies $V_k \mid Y_1, \ldots, Y_n \sim \mathcal{N}_d(n_k^{1/2}\mu_k, \Gamma_{\mathrm{w}})$. Defining $N := (n_1^{1/2}, \ldots, n_K^{1/2})^\top$ and $P := NN^\top/n \in \mathbb{R}^{K \times K}$, we may write

$$n\hat{\Gamma}_{\mathrm{b}} = \sum_{k=1}^{K} n_k \hat{\mu}_k \hat{\mu}_k^\top - n\hat{\mu}\hat{\mu}^\top = V^\top(I_K - P)V,$$

where $V := (V_1, \ldots, V_K)^\top$, and where $\hat{\mu} := n^{-1}\sum_{i=1}^{n} Z_i = \sum_{k=1}^{K}(n_k/n)\hat{\mu}_k$. By Lemma S12, we deduce that $n\hat{\Gamma}_{\mathrm{b}}$ conditional on $Y_1, \ldots, Y_n$ has a d-dimensional non-central Wishart distribution with $K - 1$ degrees of freedom, covariance matrix Γ_{w} and non-centrality matrix

$n\tilde{\Gamma}_{\mathrm{b}}^{(2)} := \sum_{k=1}^{K} n_k(\mu_k - \tilde{\mu})(\mu_k - \tilde{\mu})^\top$, which we denote as

$$n\hat{\Gamma}_{\mathrm{b}} \mid Y_1, \ldots, Y_n \sim \mathcal{W}_d(K-1, \Gamma_{\mathrm{w}}; n\tilde{\Gamma}_{\mathrm{b}}^{(2)});$$

a formal definition is given just before Lemma S12. For any fixed $u \in \mathcal{S}^{d-1}$, we have by **?**, Theorem 10.3.6 that

$$u^\top \hat{\Gamma}_{\mathrm{b}} u \mid Y_1, \ldots, Y_n \sim \frac{u^\top \Gamma_{\mathrm{w}} u}{n} \chi^2_{K-1}\biggl(\frac{n u^\top \tilde{\Gamma}_{\mathrm{b}}^{(2)} u}{u^\top \Gamma_{\mathrm{w}} u}\biggr),$$

where $\chi^2_r(\lambda)$ denotes a non-central chi-squared distribution with r degrees of freedom and non-centrality parameter λ. Letting u be the jth standard basis vector in $\mathbb{R}^p$, by **?**, Lemma 8.1, for every $\delta' \in (0, 1/2]$, we have with probability at least $1 - 2\delta'$ conditional on $Y_1, \ldots, Y_n$, that

$$\begin{aligned} \bigl|\hat{\mathcal{D}}_{\mathrm{b},j} - \tilde{\mathcal{D}}_{\mathrm{b},j}^{(2)}\bigr| &\leq \frac{\mathcal{D}_{\mathrm{w},j}}{n}\Biggl\{K + 2\sqrt{\biggl(K + \frac{2n\tilde{\mathcal{D}}_{\mathrm{b},j}^{(2)}}{\mathcal{D}_{\mathrm{w},j}}\biggr)\log(1/\delta') + 2\log(1/\delta')}\Biggr\} \\ &\leq \frac{\mathcal{D}_{\mathrm{w},j}}{n}\bigl\{2K + 3\log(1/\delta')\bigr\} + \sqrt{\frac{8\mathcal{D}_{\mathrm{w},j}\tilde{\mathcal{D}}_{\mathrm{b},j}^{(2)}\log(1/\delta')}{n}} \\ &\leq \frac{3\bigl(K + \log(1/\delta')\bigr)\|\mathcal{D}_{\mathrm{w}}\|_\infty}{n} + \sqrt{\frac{8\|\mathcal{D}_{\mathrm{w}}\|_\infty \|\tilde{\mathcal{D}}_{\mathrm{b}}^{(2)}\|_\infty \log(1/\delta')}{n}}. \end{aligned} \tag{S9}$$

Hence, by a union bound, and taking $\delta' := \delta/(8d)$, we have with probability at least $1 - \delta/4$ conditional on $Y_1, \ldots, Y_n$ that

$$\|\hat{\mathcal{D}}_{\mathrm{b}} - \mathcal{D}_{\mathrm{b}}\|_\infty \leq \frac{3R_2\bigl(K + \log(8d/\delta)\bigr)}{n} + \sqrt{\frac{8R_1^2 R_2 \log(8d/\delta)}{n}}. \tag{S10}$$

Combining (S6), (S7), (S8) and (S10), we have that the first of the desired bounds holds with probability at least $1 - 3\delta/4$.

We now turn to control $\|\hat{\mathcal{D}}_{\mathrm{w}} - \mathcal{D}_{\mathrm{w}}\|_\infty$. Let $Q := \sum_{k=1}^{K} (n_k^{-1} \mathbb{1}_{\{Y_i = k, Y_{i'} = k\}})_{i,i'=1}^{n} \in \mathbb{R}^{n \times n}$, so that

$$n\hat{\Gamma}_{\mathrm{w}} = \sum_{i=1}^{n} Z_i Z_i^\top - \sum_{k=1}^{K} n_k \hat{\mu}_k \hat{\mu}_k^\top = Z^\top (I - Q) Z,$$

where $Z := (Z_1, \ldots, Z_n)^\top$. It therefore follows again by Lemma S12 that $\hat{\Gamma}_{\mathrm{w}} \mid Y_1, \ldots, Y_n \sim n^{-1}\mathcal{W}_d(n-K, \Gamma_{\mathrm{w}})$. Another application of ?, Theorem 10.3.6 then yields for any $u \in \mathcal{S}^{d-1}$ that

$$u^\top \hat{\Gamma}_{\mathrm{w}} u \mid Y_1, \ldots, Y_n \sim \frac{u^\top \Gamma_{\mathrm{w}} u}{n} \chi^2_{n-K}.$$

Taking u to be the jth canonical basis vector again, by ?, Lemma 1, we have with probability at least $1 - 2\delta'$ that

$$|\hat{\mathcal{D}}_{\mathrm{w},j} - \mathcal{D}_{\mathrm{w},j}| \leq \frac{R_2}{n}\bigl\{K + 2\sqrt{n\log(1/\delta')} + 2\log(1/\delta')\bigr\}.$$

Again, taking $\delta' := \delta/(8d)$ as before, by a union bound, we conclude that with probability at least $1 - \delta/4$, we have

$$\|\hat{\mathcal{D}}_{\mathrm{w}} - \mathcal{D}_{\mathrm{w}}\|_\infty \leq \frac{2R_2}{n}\Bigl\{K + \sqrt{n\log(8d/\delta)} + \log(8d/\delta)\Bigr\},$$

as desired. □

Proof of Theorem 3. Define $\delta := \binom{p}{d}^{-1}\varepsilon$. Since Algorithm 2 is permutation-equivariant, by a union bound, it suffices to show that for every $P \in \mathcal{P}_d$, with probability at least $1-\delta$, the desired upper bound holds for $\bigl\|\psi\bigl((PX_i, Y_i)_{i\in[n]}\bigr) - w^P\bigr\|_\infty$. Recall that $n_k := \sum_{i=1}^n \mathbb{1}_{\{Y_i=k\}}$. Write $\Gamma_{\mathrm{w},P} := P\Sigma_{\mathrm{w}}P^\top$ and $\Gamma_{\mathrm{b},P} := P\Sigma_{\mathrm{b}}P^\top$, $\hat{\mu}_{k,P} := n_k^{-1}\sum_{i:y_i=k} PX_i$ (with $\hat{\mu}_{k,P} := 0$ if $n_k = 0$), $\hat{\mu}_P := n^{-1}\sum_{i=1}^n PX_i$,

$$\hat{\Gamma}_{\mathrm{w},P} := \frac{1}{n}\sum_{i=1}^n (PX_i - \hat{\mu}_{Y_i,P})(PX_i - \hat{\mu}_{Y_i,P})^\top \quad \text{and} \quad \hat{\Gamma}_{\mathrm{b},P} := \sum_{k=1}^K \frac{n_k}{n}(\hat{\mu}_{k,P} - \hat{\mu}_P)(\hat{\mu}_{k,P} - \hat{\mu}_P)^\top.$$

Below, we will denote $\mathcal{D}_{\mathrm{w},P} := \overrightarrow{\mathrm{diag}}(\Gamma_{\mathrm{w},P})$, and define $\mathcal{D}_{\mathrm{b},P}, \hat{\mathcal{D}}_{\mathrm{w},P}, \hat{\mathcal{D}}_{\mathrm{w},P}$ similarly. By Proposition S1 for $c_1 \leq 1/(8R_2^2)$, there is an event Ω_P with probability at least $1-\delta$, on which

$$\begin{aligned} \|\hat{\mathcal{D}}_{\mathrm{b},P} - \mathcal{D}_{\mathrm{b},P}\|_\infty &\lesssim_{R_1,R_2} \frac{K + \log(d/\delta)}{n} + \sqrt{\frac{\log(d/\delta)}{n}} \lesssim_{R_2} 1, \\ \|\hat{\mathcal{D}}_{\mathrm{w},P} - \mathcal{D}_{\mathrm{w},P}\|_\infty &\leq \frac{2R_2\{K + \log(8d/\delta)\}}{n} + 2R_2\sqrt{\frac{\log(8d/\delta)}{n}} \leq \frac{1}{2R_2}. \end{aligned} \tag{S11}$$

We will work on this event in the proof, which ensures that all entries of $\hat{\mathcal{D}}_{\mathrm{w},P}$ are positive.

By the triangle inequality, we have

$$\begin{aligned}\big\|\psi\big((PX_i,Y_i)_{i\in[n]}\big)-w^P\big\|_\infty &= \big\|\hat{\mathcal{D}}_{\mathrm{w},P}^{-1}\odot\hat{\mathcal{D}}_{\mathrm{b},P}-\mathcal{D}_{\mathrm{w},P}^{-1}\odot\mathcal{D}_{\mathrm{b},P}\big\|_\infty \\ &\leq \|\hat{\mathcal{D}}_{\mathrm{w},P}^{-1}\odot\hat{\mathcal{D}}_{\mathrm{b},P}-\mathcal{D}_{\mathrm{w},P}^{-1}\odot\hat{\mathcal{D}}_{\mathrm{b},P}\|_\infty \\ &\qquad + \|\mathcal{D}_{\mathrm{w},P}^{-1}\odot\hat{\mathcal{D}}_{\mathrm{b},P}-\mathcal{D}_{\mathrm{w},P}^{-1}\odot\mathcal{D}_{\mathrm{b},P}\|_\infty. \end{aligned} \tag{S12}$$

For the first term in (S12), on Ω_P, we have

$$\begin{aligned}\|\hat{\mathcal{D}}_{\mathrm{w},P}^{-1}\odot\hat{\mathcal{D}}_{\mathrm{b},P}-\mathcal{D}_{\mathrm{w},P}^{-1}\odot\hat{\mathcal{D}}_{\mathrm{b},P}\|_\infty &\leq \|\hat{\mathcal{D}}_{\mathrm{w},P}^{-1}-\mathcal{D}_{\mathrm{w},P}^{-1}\|_\infty\|\hat{\mathcal{D}}_{\mathrm{b},P}\|_\infty \\ &\leq \|\mathcal{D}_{\mathrm{w},P}^{-1}\|_\infty\|\hat{\mathcal{D}}_{\mathrm{w},P}^{-1}\|_\infty\|\hat{\mathcal{D}}_{\mathrm{b},P}\|_\infty\|\hat{\mathcal{D}}_{\mathrm{w},P}-\mathcal{D}_{\mathrm{w},P}\|_\infty \\ &\leq \frac{\big(\|\mathcal{D}_{\mathrm{b},P}\|_\infty+\|\hat{\mathcal{D}}_{\mathrm{b},P}-\mathcal{D}_{\mathrm{b},P}\|_\infty\big)\|\hat{\mathcal{D}}_{\mathrm{w},P}-\mathcal{D}_{\mathrm{w},P}\|_\infty}{\min(\mathcal{D}_{\mathrm{w},P})\big(\min(\mathcal{D}_{\mathrm{w},P})-\|\hat{\mathcal{D}}_{\mathrm{w},P}-\mathcal{D}_{\mathrm{w},P}\|_\infty\big)} \\ &\leq \frac{R_2\big(R_1^2+\|\hat{\mathcal{D}}_{\mathrm{b},P}-\mathcal{D}_{\mathrm{b},P}\|_\infty\big)\|\hat{\mathcal{D}}_{\mathrm{w},P}-\mathcal{D}_{\mathrm{w},P}\|_\infty}{\big(1/R_2-\|\hat{\mathcal{D}}_{\mathrm{w},P}-\mathcal{D}_{\mathrm{w},P}\|_\infty\big)} \\ &\lesssim_{R_1,R_2} \|\hat{\mathcal{D}}_{\mathrm{w},P}-\mathcal{D}_{\mathrm{w},P}\|_\infty \lesssim_{R_2} \frac{K}{n}+\sqrt{\frac{\log(d/\delta)}{n}}, \end{aligned} \tag{S13}$$

where we used (S11) in the penultimate inequality. For the second term in (S12), we also have on Ω_P that

$$\begin{aligned}\big\|\mathcal{D}_{\mathrm{w},P}^{-1}\odot\hat{\mathcal{D}}_{\mathrm{b},P}-\mathcal{D}_{\mathrm{w},P}^{-1}\odot\mathcal{D}_{\mathrm{b},P}\big\|_\infty &\leq \|\mathcal{D}_{\mathrm{w},P}^{-1}\|_\infty\|\hat{\mathcal{D}}_{\mathrm{b},P}-\mathcal{D}_{\mathrm{b},P}\|_\infty \\ &\lesssim_{R_1,R_2} \frac{K}{n}+\sqrt{\frac{\log(d/\delta)}{n}}. \end{aligned} \tag{S14}$$

The desired result follows by combining (S13) and (S14). □

S1.4 Proofs of Proposition 5 and Theorem 6

In the proof of Proposition 5, we show the convergence of the EM iterates $\hat{\mu}^{(t)}$ by analyzing their components parallel and orthogonal to μ^* separately. Writing $\eta := \mu^*/\|\mu^*\|$, let $\alpha_t \in \mathbb{R}$, $\beta_t \geq 0$ be defined by

$$\hat{\mu}^{(t)} = \alpha_t\eta + \beta_t\xi_t, \tag{S15}$$

where $\xi_t \in \mathcal{S}^{d-1}$ is orthogonal to η. Our proof will combine several propositions that control α_t and β_t under different conditions. We begin by laying some groundwork and defining

some quantities that will be used throughout this subsection.

First, it will be convenient to relabel the two classes as $\{-1, 1\}$ instead of $\{1, 2\}$. By the rotational symmetry of the problem, we may assume without loss of generality that $\mu^* = (s, 0, \ldots, 0)^\top \in \mathbb{R}^d$ for some $s \geq 0$, and that the first n_{L} observations are labeled (i.e., $Y_i \neq 0$ for $i \in [n_{\mathrm{L}}]$). We assume throughout this section that $s \leq r$ and $r \geq 1$. Let $\hat{\mu}_{n_{\mathrm{L}}} := n_{\mathrm{L}}^{-1} \sum_{i=1}^{n_{\mathrm{L}}} Z_i Y_i$, with the convention that $\hat{\mu}_{n_{\mathrm{L}}} := 0$ if $n_{\mathrm{L}} = 0$, and define the function $f_{n_{\mathrm{U}}} : \mathbb{R}^d \to \mathbb{R}^d$ by

$$f_{n_{\mathrm{U}}}(v) := \frac{1}{n_{\mathrm{U}}} \sum_{i=n_{\mathrm{L}}+1}^{n} Z_i \tanh\langle Z_i, v\rangle, \tag{S16}$$

with $f_{n_{\mathrm{U}}} := 0$ if $n_{\mathrm{U}} = 0$. Throughout, and without further comment, we assume that $n = n_{\mathrm{L}} + n_{\mathrm{U}} \geq 2$. In this notation, the EM update (11) can be rewritten, defining the function $g_n : \mathbb{R}^d \to \mathbb{R}^d$, as

$$\hat{\mu}^{(t)} = g_n(\hat{\mu}^{(t-1)}) := \gamma \hat{\mu}_{n_{\mathrm{L}}} + (1-\gamma) f_{n_{\mathrm{U}}}(\hat{\mu}^{(t-1)}).$$

The corresponding population quantities are

$$f(v) := \mathbb{E} Z_1 \tanh\langle v, Z_1\rangle \quad \text{and} \quad g(v) := \gamma \mu^* + (1-\gamma) f(v).$$

Writing $\Delta_{n_{\mathrm{U}}} := f_{n_{\mathrm{U}}} - f$, we have

$$g_n(v) = g(v) + (1-\gamma)\Delta_{n_{\mathrm{U}}}(v) + \gamma(\hat{\mu}_{n_{\mathrm{L}}} - \mu^*). \tag{S17}$$

For $\omega, \phi > 0$ and $r \geq 1$, we define the following two events that control the terms in the EM iteration involving the unlabeled and labeled data respectively:

$$\begin{aligned} \Omega_1(\omega) &:= \Bigl\{ \sup_{v \in \mathbb{R}^d} \|g_n(v)\| \leq 2(r + \sqrt{d}) \Bigr\} \cap \Bigl\{ \sup_{\substack{\|v\| \leq 2(r+\sqrt{d}) \\ v \neq 0}} \frac{\|\Delta_{n_{\mathrm{U}}}(v)\|}{\|v\|} \leq \omega \Bigr\} \\ \Omega_2(\phi) &:= \bigl\{ \|\hat{\mu}_{n_{\mathrm{L}}} - \mu^*\| \leq \phi \bigr\}. \end{aligned} \tag{S18}$$

Proposition S2. *There exists $C_r > 0$, depending only on r, such that for any $\delta \in (2e^{-n}, 1]$ and $\omega = C_r \sqrt{\frac{d \log n + \log(1/\delta)}{n_{\mathrm{U}}}}$, we have $\mathbb{P}\bigl(\Omega_1(\omega)^{\mathrm{c}}\bigr) \leq \delta$. Moreover, for any $\delta \in (0, 1]$ and for $\phi = \sqrt{\frac{2d + 3\log(1/\delta)}{n_{\mathrm{L}}}}$, we have $\mathbb{P}\bigl(\Omega_2(\phi)^{\mathrm{c}}\bigr) \leq \delta$.*

Proof. For any $v \in \mathbb{R}^d$,

$$\|g_n(v)\| = \|(1-\gamma)f_{n_{\mathrm{U}}}(v) + \gamma\hat{\mu}_{n_{\mathrm{L}}}\| \leq (1-\gamma)\cdot\frac{1}{n_{\mathrm{U}}}\sum_{i=n_{\mathrm{L}}+1}^{n}\|Z_i\| + \frac{\gamma}{n_{\mathrm{L}}}\sum_{i=1}^{n_{\mathrm{L}}}\|Z_i\|$$
$$= \frac{1}{n}\sum_{i=1}^{n}\|Z_i\| \leq \biggl(\frac{1}{n}\sum_{i=1}^{n}\|Z_i\|^2\biggr)^{1/2}.$$

Since $\sum_{i=1}^{n}\|Z_i\|^2 \sim \chi^2_{nd}(ns^2)$, by **?**, Lemma 8.1, we have with probability at least $1-\delta/2$ that

$$\sup_{v\in\mathbb{R}^d}\|g_n(v)\|^2 \leq d + s^2 + 2\sqrt{\frac{(d+2s^2)\log(2/\delta)}{n}} + \frac{2\log(2/\delta)}{n}$$
$$\leq 2d + 3s^2 + \frac{3\log(2/\delta)}{n} \leq 4(r+\sqrt{d})^2. \tag{S19}$$

Also, by a very similar argument as in the proof of Wu and Zhou (2022, Theorem 4), we have with probability at least $1-\delta/2$ that

$$\sup_{\substack{\|v\|\leq 2(r+\sqrt{d})\\ v\neq 0}}\frac{\|\Delta_{n_{\mathrm{U}}}(v)\|}{\|v\|} \leq C_r\sqrt{\frac{d\log n + \log(1/\delta)}{n_{\mathrm{U}}}}, \tag{S20}$$

for some $C_r > 0$ depending only on r. The first claim follows by combining (S19) and (S20).

For the second claim, we have $\hat{\mu}_{n_{\mathrm{L}}} \sim N_d(\mu^*, n_{\mathrm{L}}^{-1}I_d)$. Hence, by **?**, Lemma 1, we have

$$\mathbb{P}\bigl(\Omega_2(\phi)^{\mathrm{c}}\bigr) = \mathbb{P}\bigl(n_{\mathrm{L}}\|\hat{\mu}_{n_{\mathrm{L}}} - \mu^*\|^2 > n_{\mathrm{L}}\phi^2\bigr)$$
$$\leq \mathbb{P}\Bigl(n_{\mathrm{L}}\|\hat{\mu}_{n_{\mathrm{L}}} - \mu^*\|^2 > d + 2\sqrt{d\log(1/\delta)} + 2\log(1/\delta)\Bigr) \leq \delta,$$

as required. □

For any $a \in \mathbb{R}$, $b \in [0,\infty)$ and $\xi \in \mathcal{S}^{d-1}$ that is orthogonal to η, we define $F(a,b) := \eta^\top f(a\eta + b\xi)$ and $G(a,b) := \|(I_d - \eta\eta^\top)f(a\eta + b\xi)\|$. Note that the distribution of Z_1 is orthogonally invariant along the axis μ^*; in other words, if $P \in \mathbb{R}^{d\times d}$ is orthogonal and has μ^* as an eigenvector with eigenvalue 1, then $PZ_1 \overset{\mathrm{d}}{=} Z_1$. It follows that $f(a\eta + b\xi)$, and

hence $F(a,b)$ and $G(a,b)$, do not depend on ξ. We remark that

$$f(\alpha_t\eta + \beta_t\xi_t) = F(\alpha_t,\beta_t)\eta + G(\alpha_t,\beta_t)\xi'_{t+1}$$

for some $\xi'_{t+1} \in \mathcal{S}^{d-1}$ that is orthogonal to η.

Proposition S3 controls the magnitude of the component β_t of the EM algorithm iterates that is orthogonal to the signal direction η. We define $\zeta := \omega\gamma^{-1/2} \wedge \omega^{1/2}$.

Proposition S3. *Assume that $\phi\gamma^{1/2} \le \omega \le \min\{1/12, 1/(r+3)\}$ and that $\|\hat{\mu}^{(0)}\| \le r+3$. On the event $\Omega_1(\omega)\cap\Omega_2(\phi)$, we have*

$$\limsup_{t\to\infty} \beta_t \le 60(\zeta \vee r\omega).$$

Moreover, on the same event, if $\beta_{t_0} \le 60(\zeta\vee r\omega)$ for some $t_0 \in \mathbb{N}_0$, then $\beta_t \le 60(\zeta \vee r\omega)$ for all $t \ge t_0$.

Proof. We first claim that on the event $\Omega_1(\omega)\cap\Omega_2(\phi)$, we have $\|\hat{\mu}^{(t)}\| \le r+3$ for all $t \in \mathbb{N}_0$. The case $t=0$ is true by the assumption on the initializer $\hat{\mu}^{(0)}$, and if the claim holds for $t \in \mathbb{N}_0$, then since $2(r+\sqrt{d}) \ge 2(r+1) \ge r+3$, we have on $\Omega_1(\omega)\cap\Omega_2(\phi)$ that

$$\begin{aligned}
\|\hat{\mu}^{(t+1)}\| &\le (1-\gamma)\bigl\{|F(\alpha_t,\beta_t)| + G(\alpha_t,\beta_t) + \|\Delta_{n_{\mathrm{U}}}(\hat{\mu}^{(t)})\|\bigr\} + \gamma(s+\phi) \\
&\le s + 2\sqrt{2/\pi} + \omega\|\hat{\mu}^{(t)}\| + \gamma\phi \le r + 2 + \frac{\|\hat{\mu}^{(t)}\|}{r+3} \le r+3,
\end{aligned}$$

where the second inequality uses Wu and Zhou (2022, Lemma 5(5)). Moreover, from (S17), we have on $\Omega_1(\omega)\cap\Omega_2(\phi)$ that for $t\in\mathbb{N}$,

$$\begin{aligned}
\beta_{t+1} &= \bigl\|(I_d - \eta\eta^\top)\bigl\{(1-\gamma)\bigl(f(\hat{\mu}^{(t)}) + \Delta_{n_{\mathrm{U}}}(\hat{\mu}^{(t)})\bigr) + \gamma\hat{\mu}_{n_{\mathrm{L}}}\bigr\}\bigr\| \\
&\le (1-\gamma)\bigl\{G(\alpha_t,\beta_t) + \omega(|\alpha_t| + \beta_t)\bigr\} + \gamma\phi \\
&\le \beta_t(1-\gamma)\biggl\{1 + \omega - \frac{(\alpha_t^2+\beta_t^2)\wedge 1}{6}\biggr\} + \gamma\phi + \omega|\alpha_t|,
\end{aligned} \tag{S21}$$

where the final bound uses Wu and Zhou (2022, Lemma 5(8)). If $\alpha_t^2+\beta_t^2 > 1$ or $\gamma > 1/2$, then using the fact that $\omega \le 1/12$, we have from (S21) that

$$\beta_{t+1} \le \frac{11}{12}\beta_t + \gamma\phi + (r+3)\omega \le \frac{11}{12}\beta_t + (r+4)\omega. \tag{S22}$$

On the other hand, if $\alpha_t^2 + \beta_t^2 \leq 1$ and $\gamma \leq 1/2$, then

$$\beta_{t+1} \leq \beta_t \left(1 + \omega - \gamma - \frac{\alpha_t^2 + \beta_t^2}{12}\right) + \gamma\phi + \omega|\alpha_t|. \tag{S23}$$

Note that the right-hand side of (S22) is increasing in β_t and the right-hand side of (S23) is increasing in β_t for $\alpha_t^2 + \beta_t^2 \leq 1$ and $\gamma \leq 1/2$. Combining (S22) and (S23), denoting $\beta_\infty := \limsup_{t\to\infty} \beta_t$ and using the fact that $0 \leq \frac{3}{\beta_\infty}(\omega - |\alpha_t|\beta_\infty/6)^2 = 3\omega^2/\beta_\infty - \omega|\alpha_t| + \alpha_t^2\beta_\infty/12$, we have

$$\beta_\infty \leq \max\left\{\frac{11}{12}\beta_\infty + (r+4)\omega,\, \beta_\infty\left(1 + \omega - \gamma - \frac{\beta_\infty^2}{12}\right) + \gamma\phi + \frac{3\omega^2}{\beta_\infty}\right\}. \tag{S24}$$

From the first term in the maximum in (S24), we obtain

$$\beta_\infty \leq (r+38)\omega \leq 60r\omega. \tag{S25}$$

From the second term in the maximum in (S24), we obtain

$$\beta_\infty\left(\gamma - \omega + \frac{\beta_\infty^2}{12}\right) \leq \gamma\phi + \frac{3\omega^2}{\beta_\infty}. \tag{S26}$$

If $\gamma < 2\omega$, then from (S26),

$$\beta_\infty \leq 5\omega^{1/2} \leq 5\sqrt{2}\zeta, \tag{S27}$$

since otherwise we would have that the left-hand side would be at least $(-5 + 125/12)\omega^{3/2}$ and the right-hand side would at most $(\sqrt{2} + 3/5)\omega^{3/2}$, contradicting the inequality. On the other hand, if $\gamma \geq 2\omega$, then we derive from (S26) that

$$\beta_\infty^2 - 2\phi\beta_\infty - \frac{6\omega^2}{\gamma} \leq 0.$$

Solving this inequality, we find that

$$\beta_\infty \leq \phi + \sqrt{\phi^2 + 6\omega^2/\gamma} \leq 4\omega\gamma^{-1/2} = 4\zeta. \tag{S28}$$

The first claim of the proposition follows by combining (S24), (S25), (S27) and (S28). We now prove the second claim by induction on t. The base case $t = t_0$ is true by assumption, so we assume that $\beta_t \leq 60(\zeta \vee r\omega)$ for some $t \geq t_0$. Again we consider two cases. If

$\beta_t \leq 36(\zeta \vee r\omega)$, then from (S21) and using that $|\alpha_t| \leq \|\hat{\mu}^{(t)}\| \leq r+3$ for $t \geq 2$,

$$\beta_{t+1} \leq \beta_t(1+\omega) + \gamma\phi + \omega|\alpha_t| \leq 44(\zeta \vee r\omega),$$

as desired. On the other hand, if $\beta_t > 36(\zeta \vee r\omega)$, then combining (S22) and (S23), we obtain that

$$\beta_{t+1} \leq \max\biggl\{60(\zeta \vee r\omega),\ \beta_t\biggl(1+\omega-\gamma-\frac{\beta_t^2}{12}\biggr) + \gamma\phi + \frac{3\omega^2}{\beta_t}\biggr\}.$$

It suffices to show the second term in the maximum is no larger than β_t. To this end, if $\gamma \leq 2\omega$, then $\zeta^2 \leq \omega \leq 2\zeta^2$, and so

$$\begin{aligned}\beta_t\biggl(1+\omega-\gamma-\frac{\beta_t^2}{12}\biggr) + \gamma\phi + \frac{3\omega^2}{\beta_t} &\leq \beta_t + 2\zeta^2\beta_t - \frac{\beta_t^3}{12} + \gamma^{1/2}\omega + \frac{3\omega^2}{\beta_t} \\ &\leq \beta_t + 120(\zeta \vee r\omega)\zeta^2 - \frac{36^3(\zeta \vee r\omega)^3}{12} + 4\zeta^3 + \frac{\zeta^3}{3} \leq \beta_t.\end{aligned}$$

On the other hand, if $\gamma > 2\omega$, then $\zeta = \omega\gamma^{-1/2} \geq \phi$, and so

$$\begin{aligned}\beta_t\biggl(1+\omega-\gamma-\frac{\beta_t^2}{12}\biggr) + \gamma\phi + \frac{3\omega^2}{\beta_t} &\leq \beta_t - \frac{\gamma\beta_t}{2} + \gamma\phi + \frac{3\omega^2}{\beta_t} \\ &\leq \beta_t - 18\gamma\zeta + \gamma\zeta + \frac{\omega^2}{12\zeta} \leq \beta_t,\end{aligned}$$

as desired, which completes the induction. □

The following result bounds the magnitude of the signal component, α_t, of the EM iterates.

Proposition S4. *Assume that $\phi\gamma^{1/2} \leq \omega \leq \min\{1/12, 1/(r+3)\}$ and that $\|\hat{\mu}^{(0)}\| \leq r+3$. Then there exists $C_r > 0$, depending only on r, such that on the event $\Omega_1(\omega) \cap \Omega_2(\phi)$, we have*

$$\limsup_{t\to\infty} |\alpha_t| \leq C_r(\zeta \vee s).$$

Proof. By definition of α_{t+1} and (S17), we have for every $t \in \mathbb{N}_0$ that

$$\alpha_{t+1} = \eta^\top\bigl\{(1-\gamma)\bigl(f(\hat{\mu}^{(t)}) + \Delta_{n_{\mathrm{U}}}(\hat{\mu}^{(t)})\bigr) + \gamma\hat{\mu}_{n_{\mathrm{L}}}\bigr\}.$$

Thus, by the first claim in the proof of Proposition S3, we have on the event $\Omega_1(\omega) \cap \Omega_2(\phi)$ that

$$\big|\alpha_{t+1} - (1-\gamma)F(\alpha_t, \beta_t) - \gamma s\big| \leq (1-\gamma)\omega(|\alpha_t| + \beta_t) + \gamma\phi \tag{S29}$$

for every $t \in \mathbb{N}_0$. From Wu and Zhou (2022, Lemma 5(1) and Lemma 5(7)), $\alpha \mapsto F(\alpha, \beta)$ is an increasing and odd function satisfying $|F(\alpha,\beta) - F(\alpha, 0)| \leq (1+s^2)|\alpha|\beta^2$ for every $\alpha, \beta \in \mathbb{R}$. Hence, by (S29), we have on $\Omega_1(\omega) \cap \Omega_2(\phi)$ that

$$|\alpha_{t+1}| \leq (1-\gamma)\Big\{F(|\alpha_t|, 0) + (1+s^2)|\alpha_t|\beta_t^2 + \omega(|\alpha_t| + \beta_t)\Big\} + \gamma(s+\phi). \tag{S30}$$

Note the right-hand side of (S30) is increasing in $|\alpha_t|$. Define $\alpha_\infty := \limsup_{t\to\infty} |\alpha_t|$, so that $\alpha_\infty \leq r+3$ on $\Omega_1(\omega) \cap \Omega_2(\phi)$, again by the first claim in the proof of Proposition S3. We may also assume that $\alpha_\infty > s$, because otherwise the result is clear. Since $\alpha \mapsto F(\alpha, 0)$ is continuous, we have from (S30) that on $\Omega_1(\omega) \cap \Omega_2(\phi)$,

$$\alpha_\infty \leq (1-\gamma)\big\{F(\alpha_\infty, 0) + (1+r^2)\alpha_\infty\beta_\infty^2 + \omega(\alpha_\infty + \beta_\infty)\big\} + \gamma(s+\phi), \tag{S31}$$

where we recall that $\beta_\infty := \limsup_{t\to\infty} \beta_t$. Define $q : [0,\infty) \to \mathbb{R}$ by

$$q(\alpha) := \begin{cases} F(\alpha,0)/\alpha & \text{if } \alpha \neq 0 \\ 1+s^2 & \text{if } \alpha = 0. \end{cases} \tag{S32}$$

By Lemma S15, we have $q(s) = 1$ (which confirms that μ_* is a fixed point of the population EM iteration), and that $q'(\alpha) \leq -c_r\alpha$ for all $\alpha \in (0, r]$, where $c_r \in (0,1]$ depends only on r. Thus, dividing both sides of (S31) by α_∞, we have

$$\begin{aligned} 1 &\leq (1-\gamma)\bigg\{q(s) + \int_s^{\alpha_\infty} q'(\alpha)\,d\alpha + (1+r^2)\beta_\infty^2 + \omega\bigg(1 + \frac{\beta_\infty}{\alpha_\infty}\bigg)\bigg\} + \frac{\gamma(s+\phi)}{\alpha_\infty} \\ &\leq (1-\gamma)\bigg\{1 - \frac{c_r}{2}(\alpha_\infty^2 - s^2) + (1+r^2)\beta_\infty^2 + \omega\bigg(1 + \frac{\beta_\infty}{\alpha_\infty}\bigg)\bigg\} + \frac{\gamma(s+\phi)}{\alpha_\infty}. \end{aligned} \tag{S33}$$

Now $\beta_\infty \leq 60(1+r)\zeta$ by Proposition S3. We now claim that $\alpha_\infty \leq 4s + 120c_r^{-1/2}(1+r)^2\zeta$. Indeed, assuming the contrary, we would have $c_r\alpha_\infty^2/4 > c_r s^2/2 + (1+r^2)\beta_\infty^2$ and $\beta_\infty/\alpha_\infty < 1$. Hence from (S33), we have

$$1 \leq (1-\gamma)\bigg(1 - \frac{c_r\alpha_\infty^2}{4}\bigg) + \frac{\gamma s}{\alpha_\infty} + \bigg(2 + \frac{\gamma^{1/2}}{\alpha_\infty}\bigg)\omega.$$

We consider two cases. First, if $\gamma \leq 4\omega$, then $2\zeta \geq \omega^{1/2} \geq \gamma^{1/2}/2$ and hence

$$
\begin{aligned}
&(1-\gamma)\biggl(1 - \frac{c_r \alpha_\infty^2}{4}\biggr) + \frac{\gamma s}{\alpha_\infty} + \biggl(2 + \frac{\gamma^{1/2}}{\alpha_\infty}\biggr)\omega \\
&\leq \max\biggl\{1 - \frac{c_r\alpha_\infty^2}{4}, 0\biggr\} + \frac{\gamma}{4} + 3\omega \leq \max\biggl\{1 - \zeta^2, \frac{\gamma}{4} + 3\omega\biggr\} < 1,
\end{aligned}
$$

a contradiction. Second, if $\gamma > 4\omega$, then $\zeta = \omega\gamma^{-1/2}$ and

$$
(1-\gamma)\biggl(1 - \frac{c_r \alpha_\infty^2}{4}\biggr) + \frac{\gamma s}{\alpha_\infty} + \biggl(2 + \frac{\gamma^{1/2}}{\alpha_\infty}\biggr)\omega \leq 1 - \gamma + \frac{\gamma}{4} + 2\omega + \frac{\gamma\zeta}{\alpha_\infty} < 1,
$$

again a contradiction. This establishes the claimed upper bound on α_∞. □

Recall the definitions $L(\mu, \mu^*) = \|\mu - \mu^*\| \wedge \|\mu + \mu^*\|$. Our next result shows that if α_t ever becomes sufficiently large, then improved bounds can be derived on the limiting behaviour of α_t, β_t and $L(\hat{\mu}^{(t)}, \mu^*)$.

Proposition S5. *Assume that $\gamma \in [0, 1/2)$. Given any $c > 0$, there exists $C, c_1 > 0$, depending only on r and c, such that if $|\alpha_{t_0}| \geq cs$, $\beta_{t_0} \leq 60(\zeta \vee r\omega)$ for some iteration t_0, and $\phi\gamma^{1/2} \leq \omega \leq c_1$ and $s \geq C\zeta$, then on the event $\Omega_1(\omega) \cap \Omega_2(\phi)$, we have*

$$
\limsup_{t\to\infty} |\alpha_t - s| \lesssim_{r,c} \frac{\omega}{s} \wedge \frac{\omega}{\gamma^{1/2}}, \tag{S34}
$$

$$
\limsup_{t\to\infty} \beta_t \lesssim_{r,c} \frac{\omega}{s} \wedge \frac{\omega}{\gamma^{1/2}}, \tag{S35}
$$

$$
\limsup_{t\to\infty} L(\hat{\mu}^{(t)}, \mu^*) \lesssim_{r,c} \frac{\omega}{s} \wedge \frac{\omega}{\gamma^{1/2}}. \tag{S36}
$$

Proof. By flipping the sign of μ^* if necessary, we may assume without loss of generality that $\alpha_{t_0} \geq 0$ and that $c_1 \leq \min\{1/12, 1/(r+3)\}$. From (S29) and the argument immediately below it, we have

$$
\begin{aligned}
(1-\gamma)\Bigl\{F(\alpha_t, 0) - (1+s^2)\alpha_t\beta_t^2 - \omega(\alpha_t + \beta_t)\Bigr\} + \gamma(s - \phi) \leq \alpha_{t+1} \\
\leq (1-\gamma)\Bigl\{F(\alpha_t, 0) + (1+s^2)\alpha_t\beta_t^2 + \omega(\alpha_t + \beta_t)\Bigr\} + \gamma(s + \phi).
\end{aligned} \tag{S37}
$$

For any t such that $\alpha_t \geq cs$, since $\beta_t \leq 60r\omega^{1/2}$ by Proposition S3, we have that

$$(1+s^2)\alpha_t\beta_t^2 + \omega(\alpha_t+\beta_t) \leq \left\{(1+r^2)60^2r^2\omega + \omega\left(1+\frac{60s\zeta}{cs}\right)\right\}\alpha_t \leq c''\omega\alpha \tag{S38}$$

where $c'' := 60^2(1+r^2)r^2 + (1+60rc^{-1}C^{-1})$. Moreover, if $\alpha_t \geq cs$, then

$$\gamma\phi \leq \omega\gamma^{1/2} \leq \begin{cases} \gamma\zeta \leq C^{-1}\gamma s & \text{if } \gamma \geq \omega \\ \omega^{3/2} = \omega\zeta \leq c^{-1}C^{-1}\omega\alpha_t & \text{otherwise.} \end{cases} \tag{S39}$$

Let $c' := c'' + 2c^{-1}C^{-1}$ and define functions $H, L : [0,\infty) \to \mathbb{R}$ by

$$\begin{aligned} H(\alpha) &:= (1-\gamma)\big\{F(\alpha,0) + c'\omega\alpha\big\} + \gamma(s+\phi), \\ L(\alpha) &:= (1-\gamma)\big\{F(\alpha,0) - c'\omega\alpha\big\} + \gamma\max(s-\phi, s/2), \end{aligned}$$

From (S37), (S38) and (S39), we obtain that for $\alpha_t \geq cs$ and $C \geq 2$,

$$L(\alpha_t) \leq \alpha_{t+1} \leq H(\alpha_t). \tag{S40}$$

Define auxiliary sequences $(\alpha_t^+)_{t\geq t_0}$ and $(\alpha_t^-)_{t\geq 0}$ by $\alpha_{t_0}^+ := \alpha_{t_0} =: \alpha_{t_0}^-$ and for $t \geq t_0$,

$$\alpha_{t+1}^+ := H(\alpha_t^+) \quad \text{and} \quad \alpha_{t+1}^- := L(\alpha_t^-).$$

We first derive some properties of the two recursion maps H and L. For the former, we have by Wu and Zhou (2022, Lemma 3) that F, and hence H, is increasing and concave on $[0,\infty)$ with $H(0) > 0$ when $\gamma > 0$ and $H'(0) > \partial_1 F(0,0) > 1$ when $\gamma = 0$. Moreover, since F is bounded, we can choose $c_1 > 0$, depending only on r and c, such that $\lim_{\alpha\to\infty} H'(\alpha) = (1-\gamma)c'\omega \leq (1-\gamma)c'c_1 < 1/2$. On the other hand, we have $L(0) > 0$ when $\gamma > 0$. When $\gamma = 0$, we have $\omega^{1/2} = \zeta \leq s/C$, which means that after increasing $C \equiv C(r,c) > 0$ if necessary, $L'(0) = \partial_1 F(0,0) - c'\omega \geq 1 + s^2 - c's^2/C^2 > 1$. By Wu and Zhou (2022, Lemma 3), $\alpha \mapsto F(\alpha,0)$ is differentiable, increasing and concave for $\alpha \in [0,\infty)$. Reducing $c_1 \equiv c_1(r,c) > 0$ if necessary to ensure that $c_1 \leq \partial_1 F(r+3,0)/c'$, we have for $\alpha \in [0, r+3]$ that

$$L'(\alpha) = \partial_1 F(\alpha,0) - c'\omega \geq \partial_1 F(r+3,0) - c'c_1 \geq 0.$$

In other words, L is increasing on $[0, r+3]$, and moreover, similarly to H, it is also concave on this interval. Finally, we claim that for $\tilde{c} := \min\big\{c, 32(3+r^4)/3\big\}^{-1/2}\big\}$, and $\tilde{\alpha} := \tilde{c}s$, we have $L(\tilde{\alpha}) \geq \tilde{\alpha}$. To verify this, we note by Wu and Zhou (2022, Lemma 3(3) and

Equation (98)), we have

$$L(\tilde{\alpha}) \geq (1-\gamma)\tilde{\alpha}\left\{1+s^2-\frac{8}{3}(3+r^4)\tilde{\alpha}^2-c'\omega\right\}+2\gamma\tilde{\alpha} \geq (1-\gamma)\tilde{\alpha}\left(1+\frac{3s^2}{4}-c'\omega\right)+2\gamma\tilde{\alpha}. \quad \text{(S41)}$$

To control the right-hand side of (S41), if $\gamma \leq c'\omega$, we have $\zeta = \omega^{1/2} \wedge \omega\gamma^{-1/2} \geq (\omega/c')^{1/2}$. Hence, from the condition $s \geq C\zeta$, if we choose $C > 2c'$ (which is possible because c' is a decreasing function of C), we have $c'\omega \leq (c's/C)^2 \leq s^2/4$ and consequently the right-hand side of (S41) is at least $\tilde{\alpha}$. If $\gamma > c'\omega$, then we have $L(\tilde{\alpha}) \geq (1-\gamma)^2\tilde{\alpha} + 2\gamma\tilde{\alpha} \geq \tilde{\alpha}$ as desired. This establishes the claim.

We now show by induction that for all $t \geq t_0$,

$$\tilde{\alpha} \leq \alpha_t^- \leq \alpha_t \leq \alpha_t^+. \quad \text{(S42)}$$

The base case is clear by the definition of $\alpha_{t_0}^-$ and $\alpha_{t_0}^+$ above. Now suppose that (S42) holds for some iteration $t \geq 0$, so in particular, (S40) applies.

Using the monotonicity of H and (S40), we have $\alpha_{t+1} \leq H(\alpha_t) \leq H(\alpha_t^+) = \alpha_{t+1}^+$. Observe that $\alpha_t \leq \|\hat{\mu}^{(t)}\| \leq r+3$ by the proof of Proposition S3. Using the monotonicity of L on $[0, r+3]$, we find that $\alpha_{t+1} \geq L(\alpha_t) \geq L(\alpha_t^-) = \alpha_{t+1}^-$. Moreover,

$$\alpha_{t+1}^- = L(\alpha_t^-) \geq L(\tilde{\alpha}) \geq \tilde{\alpha},$$

which completes the induction.

To prove (S34), we will analyze the sequences $(\alpha_t^+)_{t\geq t_0}$ and $(\alpha_t^-)_{t\geq t_0}$, which sandwich $(\alpha_t)_{t\geq t_0}$. We start by considering the behaviour of $(\alpha_t^+)_{t\geq t_0}$. The properties of H derived above mean that we can apply Lemma S14 to obtain that α_t^+ converges to a limit, denoted α^+, satisfying $\alpha^+ = H(\alpha^+)$. By Lemma S15, we have $F(s,0) = s$ and so $H(s) = (1-\gamma)(s + c'\omega s) + \gamma(s+\phi) > s$. Hence from Lemma S14 again, we have $\alpha^+ > s$. On the other hand, since $F(\alpha, 0) \leq \mathbb{E}|Z_{1,1}| \leq (\mathbb{E}Z_{1,1}^2)^{1/2} \leq (1+s^2)^{1/2} \leq 1+r$, we have $\alpha^+ = H(\alpha^+) \leq (1-\gamma)(1+r) + \alpha^+/2 + \gamma r + 1 \leq r + 2 + \alpha^+/2$, so $\alpha^+ \leq 2r+4$. Recalling the definition of q from (S32), by Lemma S15 again, we have

$$q(\alpha^+) = q(s) + \int_s^{\alpha^+} q'(\alpha)\, d\alpha \leq 1 - c_2\big((\alpha^+)^2 - s^2\big)$$

for some $c_2 > 0$ depending only on r. Consequently,

$$\begin{aligned}\alpha^+ = H(\alpha^+) &= (1-\gamma)\alpha^+\bigl\{q(\alpha^+) + c'\omega\bigr\} + \gamma(s+\phi)\\ &\le (1-\gamma)\alpha^+\bigl\{1 - c_2\bigl((\alpha^+)^2 - s^2\bigr) + c'\omega\bigr\} + \gamma(s + \omega\gamma^{-1/2}),\end{aligned}$$

so

$$(\alpha^+)^2 - s^2 \le \frac{c'\omega}{c_2} - \frac{\gamma}{(1-\gamma)c_2}\frac{\alpha^+ - s - \omega\gamma^{-1/2}}{\alpha^+}. \tag{S43}$$

We now prove that

$$\alpha^+ - s \lesssim_{r,c} \frac{\omega}{s} \wedge \frac{\omega}{\gamma^{1/2}} \tag{S44}$$

by considering two cases. If $\alpha^+ \le 2s$, then from (S43), we have

$$\alpha^+ - s \le \frac{(1-\gamma)c'\omega + \gamma^{1/2}\omega/\alpha^+}{(1-\gamma)c_2(\alpha^+ + s) + \gamma/\alpha^+} \lesssim_{r,c} \frac{\omega}{s}\cdot\frac{1+\gamma^{1/2}/s}{1+\gamma/s^2} \lesssim \frac{\omega}{s} \wedge \frac{\omega}{\gamma^{1/2}}.$$

On the other hand, if $\alpha^+ > 2s$, then we have from (S43) again that

$$\frac{3(\alpha^+)^2}{4} + \frac{\gamma}{2c_2} \le (\alpha^+)^2 - s^2 + \frac{\gamma(\alpha^+ - s)}{(1-\gamma)c_2\alpha^+} \le \frac{(c' + 2\gamma^{1/2}/\alpha^+)\omega}{c_2}. \tag{S45}$$

In particular, $\gamma/2 \le (c' + \gamma^{1/2}/s)\omega \le c'\omega + C^{-1}(\gamma^{1/2}\omega^{1/2} \vee \gamma)$, so $\gamma \lesssim_{r,c} \omega$ and $\alpha^+ \gtrsim_{r,c} \zeta \gtrsim_{r,c} \gamma^{1/2}$. Consequently from (S45),

$$\alpha^+ - s \le \alpha^+ \lesssim_{r,c} \omega^{1/2} \lesssim_{r,c} \frac{\omega}{\alpha^+} \wedge \frac{\omega}{\gamma^{1/2}} \lesssim \frac{\omega}{s} \wedge \frac{\omega}{\gamma^{1/2}},$$

which establishes (S44). We now consider $(\alpha_t^-)_{t\ge t_0}$. Define $\tilde{L} : [0,\infty) \to [0,\infty)$ by $\tilde{L}(\alpha) := L\bigl(\alpha \wedge (r+3)\bigr)$. Since $\alpha_t^- \le \alpha_t \le r+3$ for all $t \ge 0$, we have $\alpha_{t+1}^- = \tilde{L}(\alpha_t^-)$ for all $t \ge t_0$. From the properties of L derived above, we see that $\tilde{L}$ satisfies the conditions of Lemma S14, and hence α_t^- converges to a limit, denoted α^-, satisfying $\alpha^- = \tilde{L}(\alpha^-) = L(\alpha^-)$. By Lemma S15, $F(s,0) = s$, so we have $\tilde{L}(s) = L(s) \le (1-\gamma)(s - c'\omega s) + \gamma s < s$, so by Lemma S14, we must have $\alpha^- < s$. By Lemma S15 again, we have

$$q(\alpha^-) = q(s) - \int_{\alpha^-}^{s} q'(\alpha)\,d\alpha \ge 1 + c_2'\bigl(s^2 - (\alpha^-)^2\bigr),$$

where $c_2' > 0$ depends only on r. Consequently, we have

$$\alpha^- = L(\alpha^-) \geq (1-\gamma)\alpha^-\bigl\{1 + c_2'\bigl(s^2 - (\alpha^-)^2\bigr) - c'\omega\bigr\} + \gamma(s - \omega\gamma^{-1/2}),$$

which after rearranging and using the fact that $\alpha^- \geq \tilde{\alpha} \gtrsim_r s$ leads to

$$s - \alpha^- \leq \frac{(1-\gamma)c'\omega + \gamma^{1/2}\omega/\alpha^-}{(1-\gamma)c_2'(s+\alpha^-) + \gamma/\alpha^-} \lesssim_{r,c} \frac{\omega}{s} \cdot \frac{1+\gamma^{1/2}/s}{1+\gamma/s^2} \lesssim \frac{\omega}{s} \wedge \frac{\omega}{\gamma^{1/2}}. \tag{S46}$$

Combining (S42), (S44) and (S46), we have established (S34).

We now turn to prove (S35). By increasing C if necessary, we have for all sufficiently large t that $s/2 \leq \alpha_t \leq 2s$. Consequently, we have by (S21) that for all large t,

$$\beta_{t+1} \leq \beta_t(1-\gamma)\biggl(1+\omega-\frac{s^2/4 \wedge 1}{6}\biggr)+\gamma\phi+2s\omega \leq \beta_t(1-\gamma)(1+\omega-c_3 s^2)+(\gamma^{1/2}+2s)\omega, \tag{S47}$$

with $c_3 := 1/(6r^2+24)$. Denote $\beta_\infty := \limsup_{t\to\infty}\beta_t$. If $\gamma \leq 2\omega$, then $\zeta \in [(\omega/2)^{1/2}, \omega^{1/2}]$ so $\omega \leq 2s^2/C^2 \leq c_3 s^2/2$ for C sufficiently large. Hence, from (S47), and the fact that $\gamma^{1/2} \leq 2\zeta \leq s$,

$$\beta_\infty \leq \frac{(\gamma^{1/2}+2s)\omega}{c_3 s^2/2} \lesssim_r \frac{\omega}{s} = \frac{\omega}{s} \wedge \frac{\omega}{\gamma^{1/2}}.$$

On the other hand, if $\gamma > 2\omega$, then $(1-\gamma)(1+\omega-c_3s^2) \leq 1-\gamma/2-c_3s^2/2$ and from (S47), we obtain

$$\beta_\infty \leq \frac{(\gamma^{1/2}+2s)\omega}{(\gamma+c_3s^2)/2} \lesssim_r \frac{\omega}{s} \cdot \frac{1+\gamma^{1/2}/s}{1+\gamma/s^2} \lesssim \frac{\omega}{s} \wedge \frac{\omega}{\gamma^{1/2}}.$$

Combining the above two cases establishes (S35).

Finally, recalling the decomposition of $\hat{\mu}^{(t)}$ in (S15), we see that (S36) follows immediately from (S34) and (S35). □

Next, we show that provided the initialisation is not too uncorrelated with the true parameter, $|\alpha_t|$ reaches a level that makes Proposition S5 applicable after a sufficient number of iterations.

Proposition S6. *Assume that $n \geq 3$, that $\phi\gamma^{1/2} \leq \omega \leq \min\{1/12, 1/(r+3)\}$ and that $\gamma \in [0, 1/2)$. Suppose that $\hat{\mu}^{(0)}$ is chosen such that $c'(\zeta \vee r\omega) \leq \|\hat{\mu}^{(0)}\| \leq 60(\zeta \vee r\omega)$ for some $c' \in (0,1)$ and that $|\langle \hat{\mu}^{(0)}/\|\hat{\mu}^{(0)}\|, \eta\rangle| \geq \sqrt{1/(d\log n_{\mathrm{U}})}$. Then there exist $c_4, c_5 > 0$,*

depending only on r and c', such that if $s \geq c_4\zeta\sqrt{d\log n_{\mathrm{U}}}$, then on $\Omega_1(\omega)\cap\Omega_2(\phi)$, we have

$$|\alpha_t| \geq c_5 s$$

for some $t > 0$.

Proof. By flipping the sign of μ^* if necessary, we may assume without loss of generality that $\alpha_0 \geq 0$. Assuming that the desired result is not true, we will prove by induction that on $\Omega_1(\omega)\cap\Omega_2(\phi)$, (a) $\alpha_t/\beta_t \geq c'/(60\sqrt{d\log n_{\mathrm{U}}})$ and (b) $\alpha_{t+1} \geq (1+\omega\sqrt{d\log n_{\mathrm{U}}})\alpha_t$ for all $t \geq 0$. We show this by first verifying the base case of (a), then proving that (a) implies (b) for each t, and finally proving that $\alpha_{t+1}/\beta_{t+1} \geq 1/(60\sqrt{d\log n_{\mathrm{U}}})$ once (b) holds for a given t.

For the base case, from the assumption on $\hat{\mu}^{(0)}$, we have

$$\frac{\alpha_0}{\beta_0} \geq \frac{\alpha_0}{\|\hat{\mu}^{(0)}\|} = \left\langle \frac{\hat{\mu}^{(0)}}{\|\hat{\mu}^{(0)}\|}, \eta \right\rangle \geq \frac{1}{\sqrt{d\log n_{\mathrm{U}}}} \geq \frac{c'}{60\sqrt{d\log n_{\mathrm{U}}}}$$

since $c' \leq 60$.

Now assume that $\alpha_t/\beta_t \geq c'/(60\sqrt{d\log n_{\mathrm{U}}})$ and that $\alpha_0 \leq \alpha_t < c_5 s$ for some $t \geq 0$. We aim to show that (b) holds for the same t, and start by controlling $e_1^\top f(\hat{\mu}^{(t)})$. Let $W = (W_1,\ldots,W_d)^\top$ be an independent copy of Z_1, independent of all other randomness in the problem, and define $u_t := \bigl\{\tanh(W_1\alpha_t + W_{-1}^\top\hat{\mu}_{-1}^{(t)}) - \tanh(W_1\alpha_t - W_{-1}^\top\hat{\mu}_{-1}^{(t)})\bigr\}/2$. Then by applying the second part of Lemma S13 with $a = W_1\alpha_t$ and $b = W_{-1}^\top\hat{\mu}_{-1}^{(t)}$ (so that $a+b = W^\top\hat{\mu}^{(t)}$), we have

$$\begin{aligned}
e_1^\top f(\hat{\mu}^{(t)}) &= \mathbb{E}\bigl\{W_1\tanh(W_1\alpha_t + W_{-1}^\top\hat{\mu}_{-1}^{(t)})\bigr\} \\
&\geq \mathbb{E}\left\{\alpha_t W_1^2 - \frac{\alpha_t^3 W_1^4}{3} - \alpha_t W_1^2 (W_{-1}^\top\hat{\mu}_{-1}^{(t)})^2\right\} + \mathbb{E}(W_1 u_t) \\
&= \alpha_t(1+s^2)(1-\beta_t^2) - \alpha_t^3(1+2s^2+s^4/3),
\end{aligned}$$

where in the final step we have used the fact that u_t is an odd function of $W_{-1}^\top\hat{\mu}_{-1}^{(t)}$, which has a symmetric distribution about 0, conditional on $(W_1,\hat{\mu}_{-1}^{(t)})$, and hence $\mathbb{E}(W_1u_t) = \mathbb{E}\bigl\{\mathbb{E}(W_1u_t \mid W_1,\hat{\mu}_{-1}^{(t)})\bigr\} = 0$. From the assumption $s \geq c_4\zeta$, and using $\beta_t \leq 60(1+r)\zeta$ from Proposition S3, we have for sufficiently large c_4 that $\beta_t^2(1+s^2) \leq \{60(1+r)\}^2(1+r^2)s^2/c_4^2 \leq s^2/4$. By choosing $c_5 > 0$, depending only on r, sufficiently small, we may assume that $\alpha_t^2(1+2s^2+s^4/3) < c_5^2(1+2r^2+r^4/3)s^2 \leq s^2/4$. Recall the definition of $f_{n_{\mathrm{U}}}$ from (S16).

Since on the event $\Omega_1(\omega) \cap \Omega_2(\phi)$, we have $\|\hat{\mu}^{(t)}\| \leq r + 3 \leq 2(r + \sqrt{d})$ as in the first line of the proof of Proposition S3, we have on the event $\Omega_1(\omega) \cap \Omega_2(\phi)$ that

$$\begin{aligned}
\alpha_{t+1} &= (1-\gamma) e_1^\top f_{n_{\mathrm{U}}}(\hat{\mu}^{(t)}) + \gamma e_1^\top \hat{\mu}_{n_{\mathrm{L}}} \\
&\geq (1-\gamma)\bigl\{e_1^\top f(\hat{\mu}^{(t)}) - \omega \|\hat{\mu}^{(t)}\|\bigr\} + \gamma(s - \phi) \\
&\geq (1-\gamma)\bigl\{\alpha_t(1 + s^2/2) - \omega(\alpha_t + \beta_t)\bigr\} + \gamma(s - \phi).
\end{aligned}$$

If $\gamma \geq \omega$, then $\phi \leq \omega\gamma^{-1/2} = \zeta \leq s/2$ (assuming $c_4 \geq 2$). If $\gamma < \omega$, then

$$\gamma\phi \leq \omega\gamma^{1/2} < \omega^{3/2} = \omega\zeta \leq \frac{\alpha_0 \omega}{c'}\sqrt{d \log n_{\mathrm{U}}} \leq \frac{2(1-\gamma)\alpha_t \omega}{c'}\sqrt{d \log n_{\mathrm{U}}}.$$

Hence, in either case, we have on $\Omega_1(\omega) \cap \Omega_2(\phi)$ that

$$\begin{aligned}
\alpha_{t+1} &\geq (1-\gamma)\alpha_t\bigl\{1 + s^2/2 - \omega(1 + 62/c')\sqrt{d \log n_{\mathrm{U}}}\bigr\} + \frac{\gamma s}{2} \\
&\geq (1-\gamma)\alpha_t\bigl\{1 + s^2/2 + (1 + 62/c')(2\gamma - \omega\sqrt{d \log n_{\mathrm{U}}})\bigr\},
\end{aligned}$$

where the final bound holds provided we reduce c_5 to be at most $1/(2 + 124/c')$ if necessary.

Now, when $\gamma \leq \omega\sqrt{d \log n_{\mathrm{U}}}$, we have $\zeta = \omega^{1/2} \wedge \omega\gamma^{-1/2} \geq \omega^{1/2}(d \log n_{\mathrm{U}})^{-1/4}$ and hence by the condition on s in the proposition, we have $s^2 \geq c_4^2 \zeta^2 d \log n_{\mathrm{U}} \geq c_4^2 \omega\sqrt{d \log n_{\mathrm{U}}}$. Thus, by increasing c_4 to be at least $\sqrt{4 + 248/c'}$ if necessary, we have $(1 + 62/c')\omega\sqrt{d \log n_{\mathrm{U}}} \leq s^2/4$. Hence, in this case, and on the event $\Omega_1(\omega) \cap \Omega_2(\phi)$,

$$\alpha_{t+1} \geq (1-\gamma)(1 + s^2/4 + (2 + 124/c')\gamma)\alpha_t \geq \left(1 + \frac{s^2}{8}\right)\alpha_t \geq (1 + \omega\sqrt{d \log n_{\mathrm{U}}})\alpha_t.$$

On the other hand, when $\gamma > \omega\sqrt{d \log n_{\mathrm{U}}}$, we have

$$\alpha_{t+1} \geq (1-\gamma)(1 + (1 + 62/c')\gamma)\alpha_t \geq (1 + \gamma)\alpha_t \geq (1 + \omega\sqrt{d \log n_{\mathrm{U}}})\alpha_t.$$

Combining the two bounds above proves (b) for this given t.

It remains to verify (a) for $t + 1$, assuming that (a) and (b) hold up to and including t. Since $\beta_0 \leq \|\hat{\mu}^{(0)}\| \leq 60(\zeta \vee r\omega)$, we have by Proposition S3 that $\beta_{t+1} \leq 60(\zeta \vee r\omega) \leq 60(c')^{-1}\|\hat{\mu}^{(0)}\|$. Thus,

$$\frac{\alpha_{t+1}}{\beta_{t+1}} \geq \frac{\alpha_0}{60\|\hat{\mu}^{(0)}\|/c'} \geq \frac{c'}{60\sqrt{d \log n_{\mathrm{U}}}},$$

which completes the induction. In particular, the geometric growth of α_t implied by (b) means that α_t will exceed $c_5 s$ for sufficiently large $t > 0$. This establishes our desired contradiction, and hence proves the result. $\square$

Proof of Proposition 5. Define $\phi_0 := \omega_0 \gamma^{-1/2}$, and recall the definitions of $\Omega_1(\omega)$ and $\Omega_2(\phi)$ from (S18). By Proposition S2, there exists $C_r \geq 1$, depending only on r, such that for $\omega = C_r \omega_0$ and $\phi = C_r \phi_0$, we have $\mathbb{P}\big(\Omega_1(\omega) \cap \Omega_2(\phi)\big) \geq 1 - 2\delta$.

(i) By the definition of ω and ϕ, we have $\omega = \phi\gamma^{1/2}$. If we choose c such that $c \leq C_r^{-1} \min\{1/12, 1/(r+3)\}$, then $\omega \leq \min\{1/12, 1/(r+3)\}$. Thus, we may apply Propositions S3 and S4 to obtain that on $\Omega_1(\omega) \cap \Omega_2(\phi)$, we have

$$\limsup_{t\to\infty} \|\hat{\mu}^{(t)} - \mu^*\| \leq \limsup_{t\to\infty} (|\alpha_t| + \beta_t + \|\mu^*\|) \lesssim_r \zeta \vee \|\mu^*\|.$$

The first claim follows.

(ii) From Lemma S16 and by considering the case $d = 1$ separately, for the chosen η_0, we have

$$\mathbb{P}\big(|e_1^\top \eta_0| \leq 1/\sqrt{d \log n_{\mathrm{U}}}\big) \leq \sqrt{\frac{2}{\pi \log n_{\mathrm{U}}}}.$$

Again, if we choose $c \leq C_r^{-1} \min\{1/12, 1/(r+3)\}$, then $\phi\gamma^{1/2} = \omega \leq \min\{1/12, 1/(r+3)\}$. Also, $\|\hat{\mu}^{(0)}\| = \zeta_0 \vee r\omega_0 \in [C_r^{-1}(\zeta \vee r\omega), \zeta \vee r\omega]$. Thus, applying Proposition S6 with $c' = 1/C_r$, there exists $c > 0$, depending only on r, and $t_0 \in \mathbb{N}$ such that on $\Omega_1(\omega) \cap \Omega_2(\phi) \cap \{|e_1^\top \eta_0| > 1/\sqrt{d \log n_{\mathrm{U}}}\}$, we have $|\alpha_{t_0}| \geq cs$.

Since $\beta_0 \leq \|\hat{\mu}^{(0)}\| \leq \zeta \vee r\omega$, we can apply Proposition S3 to obtain that $\beta_t \leq 60(\zeta \vee r\omega)$ for all $t \geq 0$. Hence all conditions of Proposition S5 are satisfied, and the desired result then follows from (S36). $\square$

To prove Theorem 6, we need the following proposition, which relates the loss of estimating μ^* to the operator norm loss of estimating $\mu^* \mu^{*\top}$.

Proposition S7. *Assume that $n \geq 3$ and that $(Z_1, Y_1, Y_1^*), \ldots, (Z_n, Y_n, Y_n^*)$ are independent with*

$$Y_i^* \sim \mathrm{Unif}\{-1, 1\},\ Z_i \mid Y_i^* \sim \mathcal{N}_d(Y_i^* \mu^*, I_d),\ Y_i = Y_i^* \mathbb{1}_{\{i \leq n_{\mathrm{L}}\}} \quad \textit{for } i \in [n].$$

For $\mu \in \mathbb{R}^d$ and $i \in [n]$, let $L_i(\mu) := Y_i \mathbb{1}_{\{Y_i \neq 0\}} + \tanh\langle Z_i, \mu\rangle \mathbb{1}_{\{Y_i = 0\}}$, $\mu_{\mathrm{tot}}(\mu) := \mu n^{-1} \sum_{i=1}^n L_i(\mu)$ and $\Sigma_{\mathrm{b}}(\mu) := \mu\mu^\top - \mu_{\mathrm{tot}}(\mu)\mu_{\mathrm{tot}}(\mu)^\top$. For any $\delta \in (0, 1)$ and $B > 0$, we have with probability

at least $1-\delta$ *that*

$$\sup_{\mu:\|\mu\|\le B}\bigl\{\|\Sigma_{\mathrm{b}}(\mu)-\mu^*\mu^{*\top}\|_{\mathrm{op}}-(B+s)L(\mu,\mu^*)\bigr\}\lesssim B^2(s^2\vee 1)\biggl(\frac{d\log(2Bn+e)+\log(1/\delta)}{n}\biggr).$$

Proof. For any $\mu\in\mathbb{R}^d$ with $\|\mu\|\le B$, we have

$$\begin{aligned}\|\Sigma_{\mathrm{b}}(\mu)-\mu^*\mu^{*\top}\|_{\mathrm{op}}&\le\|\mu\mu^\top-\mu^*\mu^{*\top}\|_{\mathrm{op}}+\|\mu_{\mathrm{tot}}(\mu)\mu_{\mathrm{tot}}(\mu)^\top\|_{\mathrm{op}}\\&\le(B+s)L(\mu,\mu^*)+B^2\biggl(\frac{1}{n}\sum_{i=1}^n L_i(\mu)\biggr)^2.\end{aligned}$$

Thus, it is enough to show that $\sup_{\mu:\|\mu\|\le B} n^{-1}\sum_{i=1}^n L_i(\mu)\lesssim\sqrt{\frac{(s^2\vee 1)\{d\log(2Bn+e)+\log(1/\delta)\}}{n}}$ with probability at least $1-\delta$. To this end, we have

$$\sup_{\mu:\|\mu\|\le B}\frac{1}{n}\sum_{i=1}^n L_i(\mu)=\frac{1}{n}\sum_{i=1}^{n_{\mathrm{L}}}Y_i+\frac{1}{n}\sup_{\mu:\|\mu\|\le B}\sum_{i=n_{\mathrm{L}}+1}^{n}\tanh\langle Z_i,\mu\rangle. \tag{S48}$$

For the first term on the right-hand side of (S48), by Hoeffding's inequality, we have

$$\mathbb{P}\biggl(\frac{1}{n}\sum_{i=1}^{n_{\mathrm{L}}}Y_i>\frac{\sqrt{2n_{\mathrm{L}}\log(3/\delta)}}{n}\biggr)\le\frac{\delta}{3}. \tag{S49}$$

For the second term on the right-hand side of (S48), let $\mathcal{N}$ be a ε-net of $\{v:\|v\|\le B\}$ with respect to the Euclidean distance, for some $\varepsilon\in(0,1/2]$ to be specified later. Since a maximal ε-packing set is an ε-net, we may assume that $|\mathcal{N}|\le(B+\varepsilon/2)^d/(\varepsilon/2)^d=(2B/\varepsilon+1)^d$. Using the fact that $x\mapsto\tanh x$ is 1-Lipschitz, together with the Cauchy–

Schwarz inequality, we have

$$\begin{aligned}
&\sup_{v:\|v\|\le B}\sum_{i=n_{\mathrm{L}}+1}^{n}\tanh\langle Z_i,v\rangle \\
&\qquad\le \sup_{v\in\mathcal{N}}\sum_{i=n_{\mathrm{L}}+1}^{n}\tanh\langle Z_i,v\rangle+\sup_{u,v:\|u-v\|\le\varepsilon}\sum_{i=n_{\mathrm{L}}+1}^{n}(\tanh\langle Z_i,u\rangle-\tanh\langle Z_i,v\rangle) \\
&\qquad\le \sup_{v\in\mathcal{N}}\sum_{i=n_{\mathrm{L}}+1}^{n}\tanh\langle Z_i,v\rangle+\varepsilon\sum_{i=n_{\mathrm{L}}+1}^{n}\|Z_i\|.
\end{aligned}$$

Hence taking $\varepsilon = 1/n$, and defining $\tau := \frac{\log(3/\delta)}{d\log(2Bn+e)} > 0$, we have

$$\begin{aligned}
&\mathbb{P}\biggl(\frac{1}{n}\sup_{v:\|v\|\le B}\sum_{i=n_{\mathrm{L}}+1}^{n}\tanh\langle Z_i,v\rangle > 2\sqrt{\frac{2(s^2\vee 1)(1+\tau)d\log(2Bn+e)}{n}}\biggr) \\
&\le \mathbb{P}\biggl(\frac{1}{n}\sup_{v\in\mathcal{N}}\sum_{i=n_{\mathrm{L}}+1}^{n}\tanh\langle Z_i,v\rangle > \sqrt{\frac{2(s^2\vee 1)(1+\tau)d\log(2Bn+e)}{n}}\biggr) \\
&\qquad\qquad + \mathbb{P}\biggl(\frac{1}{n}\sum_{i=n_{\mathrm{L}}+1}^{n}\|Z_i\| \ge \sqrt{2(s^2\vee 1)(1+\tau)nd\log(2Bn+e)}\biggr) \\
&\le \frac{|\mathcal{N}|}{e^{(1+\tau)d\log(2Bn+e)}} + \mathbb{P}\biggl(\frac{1}{n}\sum_{i=n_{\mathrm{L}}+1}^{n}\|Z_i\|^2 \ge 2(s^2\vee 1)(1+\tau)nd\log(2Bn+e)\biggr) \le \frac{2\delta}{3}, \qquad \text{(S50)}
\end{aligned}$$

where the penultimate bound uses Hoeffding's inequality and the Cauchy–Schwarz inequality and the final bound uses the fact that $\sum_{i=n_{\mathrm{L}}+1}^{n}\|Z_i\|^2 \sim \chi^2_{n_{\mathrm{U}}d}(n_{\mathrm{U}}s^2)$ and **?**, Lemma 8.1. Combining (S48), (S49) and (S50), we have with probability at least $1-\delta$ that

$$\begin{aligned}
\sup_{\mu:\|\mu\|\le B}\frac{1}{n}\sum_{i=1}^{n}L_i(\mu) &\le \frac{\sqrt{2n_{\mathrm{L}}\log(3/\delta)}}{n} + 2\sqrt{\frac{2(s^2\vee 1)(1+\tau)d\log(2Bn+e)}{n}} \\
&\lesssim \sqrt{\frac{(s^2\vee 1)\{d\log(2Bn+e)+\log(1/\delta)\}}{n}},
\end{aligned}$$

as desired. □

Proof of Theorem 6. We write $\hat{\mu}_{[m]} \equiv \hat{\mu}_{[m]}^{(T)}$ for the Tth (final) iterate of the EM update in

Algorithm 3 starting from the mth random initializer $\hat{\mu}^{(0)}_{[m]}$. Let $\omega = C_r\omega_0$, $\phi = C_r\phi_0$, $\Omega_1(\omega)$ and $\Omega_2(\phi)$ be defined as in the proof of Proposition 5. Further, let $\Gamma_{\mathrm{b}}(\mu)$ be defined as in Proposition S7. By Proposition S2, the first claim in the proof of Proposition S3 and Proposition S7, we have for some $C > 0$ depending only on r that

$$\mathbb{P}\biggl[\max_{m\in[M]}\sup_{T\in\mathbb{N}}\Bigl\{\|\Gamma_{\mathrm{b}}(\hat{\mu}^{(T)}_{[m]}) - \mu^*\mu^{*\top}\|_{\mathrm{op}} - (2r+3)L(\hat{\mu}^{(T)}_{[m]}, \mu^*)\Bigr\} \leq \frac{C\{d\log(rn)+\log(1/\delta)\}}{n}\biggr] \geq 1-\delta.$$

In this balanced two-cluster setup, for the tth EM iteration starting from the mth random initializer, we have $-\hat{\mu}_1 = \hat{\mu}_2 = \hat{\mu}$, where we suppress the dependence on t and m for convenience. For $i \geq n_{\mathrm{L}}+1$, we have $L_{i,1} = e^{z_i^\top\hat{\mu}_1}/(e^{z_i^\top\hat{\mu}_1}+e^{z_i^\top\hat{\mu}_2})$, $L_{i,2} = e^{z_i^\top\hat{\mu}_2}/(e^{z_i^\top\hat{\mu}_1}+e^{z_i^\top\hat{\mu}_2})$ and hence $L_{i,2}-L_{i,1} = \tanh\langle Z_i,\hat{\mu}\rangle$. Thus, $\hat{\mu}_{\mathrm{tot}} = n^{-1}\hat{\mu}\sum_{i=1}^n\{\tanh\langle Z_i,\hat{\mu}\rangle\mathbb{1}_{\{Y_i=0\}}+Y_i\mathbb{1}_{\{Y_i\neq 0\}}\}$. Also, we note that

$$\hat{\Gamma}_{\mathrm{b}} = \frac{1}{n}\sum_{i=1}^n\sum_{k=1}^2 L_{i,k}(\hat{\mu}_k-\hat{\mu}_{\mathrm{tot}})(\hat{\mu}_k-\hat{\mu}_{\mathrm{tot}})^\top = \frac{1}{n}\sum_{i=1}^n\sum_{k=1}^2 L_{i,k}\hat{\mu}_k\hat{\mu}_k^\top - \hat{\mu}_{\mathrm{tot}}\hat{\mu}_{\mathrm{tot}}^\top = \hat{\mu}\hat{\mu}^\top - \hat{\mu}_{\mathrm{tot}}\hat{\mu}_{\mathrm{tot}}^\top.$$

Consequently, using the notation of Proposition S7 and Algorithm 3, we have $\hat{Q} \equiv \hat{Q}^{(T)} \equiv \hat{Q}^{[\hat{m}]} = \Gamma_{\mathrm{b}}\bigl(\hat{\mu}^{(T)}_{[\hat{m}]}\bigr)$.

We consider two cases. If $\|\mu^*\| \leq \omega_0^{1/3}\wedge\zeta_0^{1/2}$, then by the proof of Proposition 5*(i)*, we have on the event $\Omega_1(\omega)\cap\Omega_2(\phi)$ that $\limsup_{T\to\infty} L(\hat{\mu}^{(T)}_{[m]},\mu^*) \lesssim_r \zeta_0 \vee \|\mu^*\| \lesssim_r \omega_0^{1/3}\wedge\zeta_0^{1/2}$ for every $m \in [M]$. Thus, by Proposition S7, with probability at least $\mathbb{P}\bigl(\Omega_1(\omega)\cap\Omega_2(\phi)\bigr)-\delta \geq 1-3\delta$, we have

$$\begin{aligned}\limsup_{T\to\infty}\|\hat{Q}-\mu^*\mu^{*\top}\|_{\mathrm{op}} &\lesssim_r (\omega_0^{1/3}\wedge\zeta_0^{1/2})\limsup_{T\to\infty}\max_{m\in[M]} L(\hat{\mu}^{(T)}_{[m]},\mu^*) \\ &\quad + (\omega_0^{2/3}\wedge\zeta_0)\biggl(\frac{d\log n+\log(1/\delta)}{n}\biggr) \\ &\lesssim_r \omega_0^{2/3}\wedge\zeta_0 = \frac{\omega_0}{\omega_0^{1/3}\wedge\zeta_0^{1/2}}\wedge\zeta_0 \leq \frac{\omega_0}{\|\mu^*\|}\wedge\zeta_0. \end{aligned} \tag{S51}$$

We now turn to the case where $\|\mu^*\| > \omega_0^{1/3}\wedge\zeta_0^{1/2}$. Let $\mathcal{M}_0$ be the set of $m \in [M]$ such that $|\langle\hat{\mu}^{(0)}_{[m]},\mu^*\rangle|/(\|\mu^*\|\|\hat{\mu}^{(0)}_{[m]}\|) \geq \sqrt{1/(d\log n_{\mathrm{U}})}$ and let $M_0 := |\mathcal{M}_0|$. By definition of the EM initializers, the random variables $\bigl\{\langle\hat{\mu}^{(0)}_{[m]},\mu^*\rangle/(\|\mu^*\|\|\hat{\mu}^{(0)}_{[m]}\|) : m \in [M]\bigr\}$ are independent,

and moreover, by Lemma S16 we have

$$\mathbb{P}\biggl(\frac{|\langle \hat{\mu}^{(0)}_{[m]}, \mu^* \rangle|}{\|\mu^*\|\|\hat{\mu}^{(0)}_{[m]}\|} \geq \sqrt{\frac{1}{d \log n_{\mathrm{U}}}}\biggr) \geq 1 - \sqrt{\frac{2}{\pi \log n_{\mathrm{U}}}} > \frac{3}{5}.$$

Defining $\Omega_3 := \{M_0 > M/2\}$, by Hoeffding's inequality, we have

$$\mathbb{P}(\Omega_3^{\mathrm{c}}) \leq e^{-M/50}.$$

Let

$$\mathcal{M}_1 := \bigl\{m \in [M] \setminus \{\hat{m}\} : \|\hat{Q}^{[m]} - \hat{Q}^{[\hat{m}]}\|_{\mathrm{op}} \leq \mathrm{median}(\|\hat{Q}^{[m']} - \hat{Q}^{[\hat{m}]}\|_{\mathrm{op}} : m' \in [M] \setminus \{\hat{m}\})\bigr\}.$$

Since $|\mathcal{M}_1 \cup \{\hat{m}\}| \geq \lceil (M-1)/2 \rceil + 1 > M/2$, we have on Ω_3 that $\mathcal{M}_0 \cap (\mathcal{M}_1 \cup \{\hat{m}\}) \neq \emptyset$. Thus, on the event $\Omega_1(\omega) \cap \Omega_2(\phi) \cap \Omega_3$, we can let $\tilde{m} := \min\bigl(\mathcal{M}_0 \cap (\mathcal{M}_1 \cup \{\hat{m}\})\bigr)$, so by definition of $\hat{m}$, we have

$$\begin{aligned}
\|\hat{Q}^{[\hat{m}]} - \mu^* \mu^{*\top}\|_{\mathrm{op}} &\leq \|\hat{Q}^{[\hat{m}]} - \hat{Q}^{[\tilde{m}]}\|_{\mathrm{op}} + \|\hat{Q}^{[\tilde{m}]} - \mu^* \mu^{*\top}\|_{\mathrm{op}} \\
&\leq \mathrm{median}(\|\hat{Q}^{[m']} - \hat{Q}^{[\tilde{m}]}\|_{\mathrm{op}} : m' \in [M] \setminus \{\hat{m}\}) + \|\hat{Q}^{[\tilde{m}]} - \mu^* \mu^{*\top}\|_{\mathrm{op}} \\
&\leq \mathrm{median}(\|\hat{Q}^{[m']} - \hat{Q}^{[\tilde{m}]}\|_{\mathrm{op}} : m' \in [M] \setminus \{\tilde{m}\}) + \|\hat{Q}^{[\tilde{m}]} - \mu^* \mu^{*\top}\|_{\mathrm{op}} \\
&\leq \max_{m, m' \in \mathcal{M}_0} \|\hat{Q}^{[m]} - \hat{Q}^{[m']}\|_{\mathrm{op}} + \|\hat{Q}^{[\tilde{m}]} - \mu^* \mu^{*\top}\|_{\mathrm{op}} \\
&\leq 3 \max_{m \in \mathcal{M}_0} \|\hat{Q}^{[m]} - \mu^* \mu^{*\top}\|_{\mathrm{op}}.
\end{aligned}$$

Since $\omega_0 \leq (d \log n)^{-3}$, by discussing cases of $\gamma < \omega$, $\omega \leq \gamma \leq \omega^{2/3}$ and $\gamma > \omega^{2/3}$, we see that $\omega_0^{1/3} \wedge \zeta_0^{1/2} \geq \zeta_0 \sqrt{d \log n}$. From the proof of Proposition 5*(ii)*, we have on the event $\Omega_1(\omega) \cap \Omega_2(\phi)$ that $\limsup_{T \to \infty} \max_{m \in \mathcal{M}_0} L(\hat{\mu}^{(T)}_{[m]}, \mu^*) \lesssim_r \frac{\omega_0}{\|\mu^*\|} \wedge (\omega_0 \gamma^{-1/2})$. Let Ω_4 be the event on which the conclusion of Proposition S7 holds. Then on $\Omega_1(\omega) \cap \Omega_2(\phi) \cap \Omega_3 \cap \Omega_4$,

we therefore have

$$\limsup_{T\to\infty} \max_{j\in[d]} \bigl|\hat{Q}_{jj}^{(T)} - (\mu_j^*)^2\bigr| \le \limsup_{T\to\infty} \|\hat{Q}^{[\hat{m}]} - \mu^*\mu^{*\top}\|_{\mathrm{op}} \tag{S52}$$

$$\lesssim_r \limsup_{T\to\infty} \max_{m\in\mathcal{M}_0} L(\hat{\mu}_{[m]}^{(T)}, \mu^*) + \frac{d\log n + \log(1/\delta)}{n}$$

$$\lesssim_r \biggl(\frac{\omega_0}{\|\mu^*\|} \wedge \frac{\omega_0}{\gamma^{1/2}}\biggr) + \frac{d\log n + \log(1/\delta)}{n}$$

$$\lesssim_r \frac{\omega_0}{\|\mu^*\|} \wedge \zeta_0. \tag{S53}$$

Since $\|\overrightarrow{\mathrm{diag}}(\hat{Q}) - (\mu^*)^{\odot 2}\|_\infty \le \|\hat{Q} - \mu^*\mu^{*\top}\|_{\mathrm{op}}$, the desired result follows by combining (S51) and (S53), and the fact that $\mathbb{P}\bigl(\Omega_1(\omega)\cap\Omega_2(\phi)\cap\Omega_3\cap\Omega_4\bigr) \ge 1 - 3\delta - e^{-M/50}$. □

S1.5 Proof of Corollary 7

Proof of Corollary 7. Fix $P \in \mathcal{P}_d$, define $Z_i := PX_i$ for $i \in [n]$, $\mu^* := P\nu^*$, $\delta := \varepsilon/\bigl\{4\binom{p}{d}\bigr\}$, $\omega_0 := \sqrt{\frac{d\log n + \log(1/\delta)}{n_{\mathrm{U}}}}$ and $\zeta_0 := \omega_0^{1/2} \wedge \omega_0\gamma^{-1/2}$. Then, provided $C_1 > 2$, we have $\delta \ge 2e^{-n/2}/p^d > 2e^{-n}$, and $\|\mu^*\| \le \|\nu^*\| \le r$. Let $c > 0$ be chosen, depending only on r, to satisfy Theorem 6. By increasing $C_1 > 0$, depending only on r, if necessary, we may assume that $\omega_0 \le \min\{c, (d\log n)^{-3}\}$. Hence, since $(P\Sigma_{\mathrm{w}}P^\top)^{-1}P\Sigma_{\mathrm{b}}P^\top = \mu^*\mu^{*\top}$, and $\overrightarrow{\mathrm{diag}}\bigl((P\Sigma_{\mathrm{w}}P^\top)^{-1}P\Sigma_{\mathrm{b}}P^\top\bigr)\bigr\|_\infty = (\mu^*)^{\odot 2}$, we can apply Theorem 6 to obtain that for some $C_2' > 0$ depending only on r, with probability at least $1 - 3\delta - e^{-M/50}$ we have that, abbreviating $\psi^{(T)} = \psi^{(M,T)}$

$$\limsup_{T\to\infty} \bigl\|\psi^{(T)}\bigl((PX_i, Y_i)_{i\in[n]}\bigr) - \overrightarrow{\mathrm{diag}}\bigl((P\Sigma_{\mathrm{w}}P^\top)^{-1}P\Sigma_{\mathrm{b}}P^\top\bigr)\bigr\|_\infty \le C_2'\zeta_0$$

$$\le C_2 \min\Biggl[\biggl\{\frac{d\log(p\vee n) + \log(1/\varepsilon)}{n}\biggr\}^{1/4}, \sqrt{\frac{d\log(p\vee n) + \log(1/\varepsilon)}{n_{\mathrm{L}}}}\Biggr] \le \frac{(\nu_{\min}^*)^2}{4d}.$$

Since $\psi^{(T)}$ is permutation equivariant for each $T \geq 0$, by Fatou's lemma and a union bound, we have that

$$\begin{aligned}
&\limsup_{T\to\infty} \mathbb{P}\biggl(\max_{P\in\mathcal{P}_d}\bigl\|\psi^{(T)}\bigl((PX_i,Y_i)_{i\in[n]}\bigr) - \overrightarrow{\mathrm{diag}}\bigl((P\Sigma_{\mathrm{w}}P^\top)^{-1}P\Sigma_{\mathrm{b}}P^\top\bigr)\bigr\|_\infty > \frac{(\nu^*_{\min})^2}{4d}\biggr) \\
&\quad\leq \mathbb{P}\biggl(\limsup_{T\to\infty}\max_{P\in\mathcal{P}_d}\bigl\|\psi^{(T)}\bigl((PX_i,Y_i)_{i\in[n]}\bigr) - \overrightarrow{\mathrm{diag}}\bigl((P\Sigma_{\mathrm{w}}P^\top)^{-1}P\Sigma_{\mathrm{b}}P^\top\bigr)\bigr\|_\infty > \frac{(\nu^*_{\min})^2}{4d}\biggr) \\
&\quad\leq \sum_{P\in\mathcal{P}_d}\mathbb{P}\biggl(\limsup_{T\to\infty}\bigl\|\psi^{(T)}\bigl((PX_i,Y_i)_{i\in[n]}\bigr) - \overrightarrow{\mathrm{diag}}\bigl((P\Sigma_{\mathrm{w}}P^\top)^{-1}P\Sigma_{\mathrm{b}}P^\top\bigr)\bigr\|_\infty > \frac{(\nu^*_{\min})^2}{4d}\biggr) \\
&\quad\leq \binom{p}{d}(3\delta + e^{-M/50}) \leq \frac{3}{4}\varepsilon + e^{-M/50+d\log p} \leq \varepsilon.
\end{aligned}$$

The result now follows from Theorem 2, noting that $\gamma_{\min} = (\nu^*_{\min})^2$ and $\gamma_{\max} = (\nu^*_{\max})^2$. □

S2 Extension to high-dimensional quadratic discriminant analysis

In this subsection, we extend our methods from Section 3.2, providing Algorithm S1 for supervised learning in the more general case of high-dimensional quadratic discriminant analysis, where each class can have a different covariance matrix. For positive integers n, d, p, for $K \geq 2$, for $(\pi_k)_{k\in[K]} \in (0,1)^K$, and for $\varepsilon > 0$, we denote

$$\tilde{E}_1\bigl(n,d,p,K,(\pi_k)_{k\in[K]},\varepsilon\bigr) := \frac{\log\bigl(dK\binom{p}{d}/\varepsilon\bigr)}{n\min_{k\in[K]}\pi_k} + \sqrt{\frac{\log\bigl(dK\binom{p}{d}/\varepsilon\bigr)}{n\min_{k\in[K]}\pi_k}}$$

and

$$\tilde{E}_2\bigl(n,d,p,K,\varepsilon\bigr) := \frac{K\log\bigl(dK\binom{p}{d}/\varepsilon\bigr)}{n} + \sqrt{\frac{K\log\bigl(dK\binom{p}{d}/\varepsilon\bigr)}{n}}.$$

The next result provides a guarantee for the output of Algorithm S1 for all axis-aligned d-dimensional projected datasets.

Theorem S8. *Fix $\varepsilon \in (0,1]$, $K \in \{2,3,\ldots,\}$, and $p,d,n \in \mathbb{N}$ with $d \leq \min(p, n-K)$. Suppose that $(X_1,Y_1),\ldots,(X_n,Y_n)$ are independent and identically distributed pairs on $\mathbb{R}^p \times [K]$, with $\mathbb{P}(Y_1 = k) = \pi_k$, and $X_1 \mid Y_1 = k \sim \mathcal{N}_p(\nu^*_k, \Sigma_{\mathrm{w},k})$ for $k \in [K]$. For*

Algorithm S1: Base learning using labeled data with class-specific covariance matrices

Input: $(z_1, y_1), \dots, (z_n, y_n) \in \mathbb{R}^d \times ([K] \cup \{0\})$. A closed constraint set $\mathcal{C} \subseteq \mathbb{S}^{d\times d}$, with default $\mathcal{C} = \mathbb{S}^{d\times d}$.

for $k \in [K]$ **do**

  Set $n_k := |\{i : y_i = k\}|$.

  Estimate the class mean $\hat{\mu}_k := n_k^{-1} \sum_{i:y_i=k} z_i$ and the within-class covariance matrix as $\hat{\Gamma}_{\mathrm{w},k} := n_k^{-1} \sum_{i:y_i=k} (z_i - \hat{\mu}_k)(z_i - \hat{\mu}_k)^\top$ (with the convention that $\hat{\mu}_k := 0$ and $\hat{\Gamma}_{\mathrm{w},k} := 0$ if $n_k = 0$).

end

Compute $n' := \sum_{k=1}^K n_k$ and $\hat{\mu} := (n')^{-1} \sum_{i=1}^n z_i$.

Output: $\psi\big((z_i, y_i)_{i\in[n]}\big) := \Big(\big(\sum_{k=1}^K \frac{n_k}{n'} \{\mathrm{Proj}_{\mathcal{C}} \hat{\Gamma}_{\mathrm{w},k}\}^{-1} (\hat{\mu}_k - \hat{\mu})(\hat{\mu}_k - \hat{\mu})^\top\big)_{j,j}\Big)_{j=1}^d$, where $\mathrm{Proj}_{\mathcal{C}} : \mathbb{S}^{d\times d} \to \mathcal{C}$ denotes the Euclidean projection operator onto $\mathcal{C}$; here we take the pseudoinverse if $\mathrm{Proj}_{\mathcal{C}} \hat{\Gamma}_{\mathrm{w}}$ is not invertible.

$P \in \mathcal{P}_d$, let $\psi\big((PX_i, Y_i)_{i\in[n]}\big)$ be the output of Algorithm S1 with input $(PX_i, Y_i)_{i\in[n]}$, and with $\mathcal{C}$ as the set of $d \times d$ diagonal positive semi-definite matrices. Suppose that $\max_{k\in[K]} \|\nu_k^* - \nu^*\|_\infty \le R_1$ where $\nu^* := \sum_{k=1}^K \pi_k \nu_k^*$, that $\Sigma_{\mathrm{w},k}$ is diagonal for all $k \in [K]$, and that $\max_{k\in[K],j\in[p]} \max\{\Sigma_{\mathrm{w},k,jj}, \Sigma_{\mathrm{w},k,jj}^{-1}\} \le R_2$ for some $R_2 \ge 1$. For $P \in \mathcal{P}_d$, define $w^P \in [0,\infty)^p$ with entries $w_j^P := \sum_{k=1}^K \pi_k (P\Sigma_{\mathrm{w},k}P^\top)^{-1}\big((P\nu_k^*)_j - (P\nu^*)_j\big)^2$. Then there exists $c_1 > 0$, depending only on R_1 and R_2, such that if $\varepsilon \in (0,1)$,

$$n \ge \frac{8\log\big(4K\binom{p}{d}/\varepsilon\big)}{\min_{k\in[K]} \pi_k} \quad \text{and} \quad \tilde{E}_1\big(n, d, p, K, (\pi_k)_{k\in[K]}, \varepsilon\big) \le c_1, \tag{S54}$$

then with probability at least $1 - \varepsilon$, we have

$$\max_{P\in\mathcal{P}_d} \big\|\psi\big((PX_i, Y_i)_{i\in[n]}\big) - w^P\big\|_\infty \lesssim_{R_1,R_2} \tilde{E}_2(n, d, p, K, \varepsilon).$$

Before presenting the proof of Theorem S8, let us first consider some implications of this result. In the classification problem with class-specific covariance matrices from The-

orem S8, define the set of signal coordinates

$$S_0 := \left\{ j \in [p] : \left(\sum_{k=1}^{K} \pi_k \Sigma_{\mathrm{w},k}^{-1} (\nu_k^* - \nu^*)^{\odot 2} \right)_{j,j} > 0 \right\}.$$

For $j \in [p]$, write $(\nu^*)_j$ for the jth coordinate of ν^*. As in Corollary 4, suppose that $R_3 > 0$ is such that

$$\min_{j \in S_0} \min_{k \in [K]} \pi_k \big(\nu_{k,j}^* - (\nu^*)_j\big)^2 \geq \frac{1}{R_3}.$$

Then by Theorem 2, we have under the conditions of Theorem S8 that there exist $C_1, C_2 > 0$, depending only on R_1, R_2 and R_3, such that whenever $C_1 \tilde{E}_2(n, d, p, K, \varepsilon) \leq 1/d$, the output $\hat{S}$ of Algorithm 1 with A groups of projections and base procedure Algorithm S1 satisfies

$$\mathbb{P}(S_0 \subseteq \hat{S}) \geq 1 - \varepsilon - p \exp\left(-\frac{A}{C_2 p^2} \right).$$

Proof of Theorem S8. The proof is similar to that of Theorem 3 from Section S1.3, so we only sketch it and highlight the differences, reusing notation from that proof. Defining $\delta := \binom{p}{d}^{-1} \varepsilon \in (0, 1)$, it suffices by a union bound to show that for every $P \in \mathcal{P}_d$, with probability at least $1 - \delta$, the desired upper bound holds for $\big\|\psi\big((PX_i, Y_i)_{i \in [n]}\big) - w^P\big\|_\infty$. For $k \in [K]$ and $P \in \mathcal{P}_d$, we write $\Gamma_{\mathrm{w},k,P} := P \Sigma_{\mathrm{w},k} P^\top$, $\Gamma_{\mathrm{b},k,P} := P(\nu_k^* - \nu^*)(\nu_k^* - \nu^*)^\top P^\top$, $\hat{\pi}_k := n_k/n$, as well as

$$\hat{\Gamma}_{\mathrm{w},k,P} := \frac{1}{n_k} \sum_{i : Y_i = k} (PX_i - \hat{\mu}_{k,P})(PX_i - \hat{\mu}_{k,P})^\top \quad \text{and} \quad \hat{\Gamma}_{\mathrm{b},k,P} := (\hat{\mu}_{k,P} - \hat{\mu}_P)(\hat{\mu}_{k,P} - \hat{\mu}_P)^\top.$$

Here, we adopt the convention that $\hat{\Gamma}_{\mathrm{w},k,P} := 0$ when $n_k = 0$. Moreover, we will denote $\mathcal{D}_{\mathrm{w},k,P} := \overrightarrow{\mathrm{diag}}(\Gamma_{\mathrm{w},k,P})$, and define $\mathcal{D}_{\mathrm{b},k,P}, \hat{\mathcal{D}}_{\mathrm{w},k,P}, \hat{\mathcal{D}}_{\mathrm{w},k,P}$ similarly. By Proposition S9, for each $P \in \mathcal{P}_d$ there exists an event Ω_P with probability at least $1 - \delta/2$, on which for all $k \in [K]$,

$$\begin{aligned} \|\hat{\mathcal{D}}_{\mathrm{w},k,P} - \mathcal{D}_{\mathrm{w},k,P}\|_\infty &\leq \frac{2R_1 \log(8dK/\delta)}{n_k} + 2R_1 \sqrt{\frac{\log(8dK/\delta)}{n_k}}, \\ \|\hat{\mathcal{D}}_{\mathrm{b},k,P} - \mathcal{D}_{\mathrm{b},k,P}\|_\infty &\leq \frac{4R_2 \log(8dK/\delta)}{n_k} + 2R_1 \sqrt{\frac{2R_2 \log(8dK/\delta)}{n_k}}. \end{aligned} \tag{S55}$$

By a multiplicative Chernoff bound (e.g. ?, Theorem 2.3(c)), the first condition in (S54),

and a union bound over $k \in [K]$, there is an event Ω'_P with probability at least $1 - \delta/4$, such that $n_k \geq n\pi_k/2$ for all $k \in [K]$. Thus, on $\Omega'_P \cap \Omega_P$, it follows that by choosing $c_1 > 0$, depending only on R_1 and R_2, sufficiently small in the second condition in (S54), we have for all $k \in [K]$ that

$$\|\hat{\mathcal{D}}_{\mathrm{w},k,P} - \mathcal{D}_{\mathrm{w},k,P}\|_\infty \leq \frac{4R_1 \log(8dK/\delta)}{n\pi_k} + 2R_1\sqrt{\frac{2\log(8dK/\delta)}{n\pi_k}} \leq \frac{1}{2R_2}, \tag{S56}$$

$$\|\hat{\mathcal{D}}_{\mathrm{b},k,P} - \mathcal{D}_{\mathrm{b},k,P}\|_\infty \leq \frac{8R_2 \log(8dK/\delta)}{n\pi_k} + 4R_1\sqrt{\frac{R_2\log(8dK/\delta)}{n\pi_k}} \lesssim_{R_1,R_2} 1. \tag{S57}$$

Observe from (S56) that $\hat{\mathcal{D}}_{\mathrm{w},k,P}$ is invertible for all $k \in [K]$ on $\Omega_P \cap \Omega'_P$. By the triangle inequality, for each $P \in \mathcal{P}_d$, we have

$$\begin{aligned}
\big\|\psi\big((PX_i, Y_i)_{i\in[n]}\big) - w^P\big\|_\infty &= \bigg\|\sum_{k=1}^K \hat{\pi}_k \hat{\mathcal{D}}_{\mathrm{w},k,P}^{-1} \odot \hat{\mathcal{D}}_{\mathrm{b},k,P} - \sum_{k=1}^K \pi_k \mathcal{D}_{\mathrm{w},k,P}^{-1} \odot \mathcal{D}_{\mathrm{b},k,P}\bigg\|_\infty \\
&\leq \bigg\|\sum_{k=1}^K (\hat{\pi}_k - \pi_k)\mathcal{D}_{\mathrm{w},k,P}^{-1} \odot \mathcal{D}_{\mathrm{b},k,P}\bigg\|_\infty + \bigg\|\sum_{k=1}^K \hat{\pi}_k(\hat{\mathcal{D}}_{\mathrm{w},k,P}^{-1} - \mathcal{D}_{\mathrm{w},k,P}^{-1}) \odot \mathcal{D}_{\mathrm{b},k,P}\bigg\|_\infty \\
&\quad + \bigg\|\sum_{k=1}^K \hat{\pi}_k \hat{\mathcal{D}}_{\mathrm{w},k,P}^{-1} \odot (\hat{\mathcal{D}}_{\mathrm{b},k,P} - \mathcal{D}_{\mathrm{b},k,P})\bigg\|_\infty \\
&\leq \sum_{k=1}^K |\hat{\pi}_k - \pi_k| \big\|\mathcal{D}_{\mathrm{w},k,P}^{-1} \odot \mathcal{D}_{\mathrm{b},k,P}\big\|_\infty + \sum_{k=1}^K \hat{\pi}_k \big\|\hat{\mathcal{D}}_{\mathrm{w},k,P}^{-1} - \mathcal{D}_{\mathrm{w},k,P}^{-1}\big\|_\infty \big\|\mathcal{D}_{\mathrm{b},k,P}\big\|_\infty \\
&\quad + \sum_{k=1}^K \hat{\pi}_k \big\|\hat{\mathcal{D}}_{\mathrm{w},k,P}^{-1}\big\|_\infty \big\|\hat{\mathcal{D}}_{\mathrm{b},k,P} - \mathcal{D}_{\mathrm{b},k,P}\big\|_\infty.
\end{aligned} \tag{S58}$$

For the first term in (S58), as in the proof of Proposition S1, we have by McDiarmid's inequality that there exists an event Ω''_P, with probability at least $1 - \delta/4$, on which

$$\sum_{k=1}^K |\hat{\pi}_k - \pi_k| \big\|\mathcal{D}_{\mathrm{w},k,P}^{-1} \odot \mathcal{D}_{\mathrm{b},k,P}\big\|_\infty \leq R_1^2 R_2 \sum_{k=1}^K |\hat{\pi}_k - \pi_k| \lesssim_{R_1,R_2} \sqrt{\frac{2\log(4/\delta)}{n}}. \tag{S59}$$

For the second term in (S58), we have by (S56) that for each $k \in [K]$ and on $\Omega_P \cap \Omega'_P$,

$$
\begin{aligned}
\big\|\hat{\mathcal{D}}_{\mathrm{w},k,P}^{-1} - \mathcal{D}_{\mathrm{w},k,P}^{-1}\big\|_\infty \big\|\mathcal{D}_{\mathrm{b},k,P}\big\|_\infty &\leq \big\|\mathcal{D}_{\mathrm{w},k,P}^{-1}\big\|_\infty \big\|\hat{\mathcal{D}}_{\mathrm{w},k,P}^{-1}\big\|_\infty \big\|\mathcal{D}_{\mathrm{b},k,P}\big\|_\infty \big\|\hat{\mathcal{D}}_{\mathrm{w},k,P} - \mathcal{D}_{\mathrm{w},k,P}\big\|_\infty \\
&\leq \frac{\big\|\mathcal{D}_{\mathrm{b},k,P}\big\|_\infty \big\|\hat{\mathcal{D}}_{\mathrm{w},k,P} - \mathcal{D}_{\mathrm{w},k,P}\big\|_\infty}{\min(\mathcal{D}_{\mathrm{w},k,P})\big(\min(\mathcal{D}_{\mathrm{w},k,P}) - \big\|\hat{\mathcal{D}}_{\mathrm{w},k,P} - \mathcal{D}_{\mathrm{w},k,P}\big\|_\infty\big)} \\
&\leq \frac{R_1^2 R_2 \big\|\hat{\mathcal{D}}_{\mathrm{w},k,P} - \mathcal{D}_{\mathrm{w},k,P}\big\|_\infty}{1/R_2 - \big\|\hat{\mathcal{D}}_{\mathrm{w},k,P} - \mathcal{D}_{\mathrm{w},k,P}\big\|_\infty} \\
&\lesssim_{R_1,R_2} \big\|\hat{\mathcal{D}}_{\mathrm{w},k,P} - \mathcal{D}_{\mathrm{w},k,P}\big\|_\infty. \qquad \text{(S60)}
\end{aligned}
$$

Similarly, for the third term in (S58), for each $k \in [K]$ and on $\Omega_P \cap \Omega'_P$,

$$
\big\|\hat{\mathcal{D}}_{\mathrm{w},k,P}^{-1}\big\|_\infty \|\hat{\mathcal{D}}_{\mathrm{b},k,P} - \mathcal{D}_{\mathrm{b},k,P}\|_\infty \leq \frac{\big\|\hat{\mathcal{D}}_{\mathrm{b},k,P} - \mathcal{D}_{\mathrm{b},k,P}\big\|_\infty}{1/R_2 - \big\|\hat{\mathcal{D}}_{\mathrm{w},k,P} - \mathcal{D}_{\mathrm{w},k,P}\big\|_\infty} \lesssim_{R_1,R_2} \big\|\hat{\mathcal{D}}_{\mathrm{b},k,P} - \mathcal{D}_{\mathrm{b},k,P}\big\|_\infty. \qquad \text{(S61)}
$$

By combining (S55), (S58), (S59), (S60) and (S61), we have on $\Omega_P \cap \Omega'_P \cap \Omega''_P$ that

$$
\begin{aligned}
&\big\|\psi\big((PX_i, Y_i)_{i\in[n]}\big) - w^P\big\|_\infty \\
&\quad\lesssim_{R_1,R_2} \sqrt{\frac{2\log(4/\delta)}{n}} + \sum_{k=1}^K \Big(\hat{\pi}_k \big\|\hat{\mathcal{D}}_{\mathrm{w},k,P} - \mathcal{D}_{\mathrm{w},k,P}\big\|_\infty + \hat{\pi}_k \big\|\hat{\mathcal{D}}_{\mathrm{b},k,P} - \mathcal{D}_{\mathrm{b},k,P}\big\|_\infty\Big) \\
&\quad\lesssim_{R_1,R_2} \sqrt{\frac{2\log(4/\delta)}{n}} + \sum_{k=1}^K \hat{\pi}_k \bigg(\frac{\log(dK/\delta)}{n_k} + \sqrt{\frac{\log(dK/\delta)}{n_k}}\bigg) \\
&\quad\lesssim \frac{K\log(dK/\delta)}{n} + \frac{\sum_{k=1}^K n_k^{1/2}\sqrt{\log(dK/\delta)}}{n} \leq \frac{K\log(dK/\delta)}{n} + \sqrt{\frac{K\log(dK/\delta)}{n}},
\end{aligned}
$$

where we used the Cauchy–Schwarz inequality in the last step. The conclusion follows since $\mathbb{P}(\Omega_P \cap \Omega'_P \cap \Omega''_P) \geq 1 - \delta$ for each $P \in \mathcal{P}_d$. □

The following proposition controls the rate of convergence of parameter estimation in a Gaussian quadratic discriminant analysis model, and in used in Theorem S8.

Proposition S9. *Suppose that* $(Z_1, Y_1), \ldots, (Z_n, Y_n)$ *are independent and identically distributed pairs, with* $\mathbb{P}(Y_1 = k) = \pi_k$ *and* $Z_1 \mid Y_1 = k \sim \mathcal{N}_d(\mu_k, \Gamma_{\mathrm{w},k})$ *for* $k \in [K]$*. Recall the definitions of* μ, $\hat{\mu}$, $\hat{\mu}_k$, n_k *from Proposition S1. For* $k \in [K]$*, write* $\Gamma_{\mathrm{b},k} := (\mu_k - \mu)(\mu_k - \mu)^\top$, $\hat{\Gamma}_{\mathrm{b},k} := (\hat{\mu}_k - \hat{\mu})(\hat{\mu}_k - \hat{\mu})^\top$*, and let* $\hat{\Gamma}_{\mathrm{w},k} := n_k^{-1}\sum_{i:Y_i=k}(Z_i - \hat{\mu}_k)(Z_i - \hat{\mu}_k)^\top$ *with the convention*

that $\hat{\Gamma}_{\mathrm{w},k} := 0$ *if* $n_k = 0$. *If* $\max_{k\in[K]} \|\mu_k - \mu\|_\infty \leq R_1$ *and* $\max_{k\in[K]} \|\overrightarrow{\mathrm{diag}}(\Gamma_{\mathrm{w},k})\|_\infty \leq R_2$ *for some* $R_1, R_2 > 0$, *then for every* $\delta \in (0,1]$, *we have with probability at least* $1-\delta$ *that for all* $k \in [K]$,

$$\big\|\overrightarrow{\mathrm{diag}}(\hat{\Gamma}_{\mathrm{w},k}) - \overrightarrow{\mathrm{diag}}(\Gamma_{\mathrm{w},k})\big\|_\infty \leq 2\|\Gamma_{\mathrm{w},k}\|_\infty \sqrt{\frac{\log(4dK/\delta)}{n_k}} + 2\frac{\|\Gamma_{\mathrm{w},k}\|_\infty \log(4dK/\delta)}{n_k} \tag{S62}$$

and

$$\big\|\overrightarrow{\mathrm{diag}}(\hat{\Gamma}_{\mathrm{b},k}) - \overrightarrow{\mathrm{diag}}(\Gamma_{\mathrm{b},k})\big\|_\infty \leq 2R_1 \sqrt{\frac{2R_2\log(4dK/\delta)}{n_k}} + \frac{4R_2\log(4dK/\delta)}{n_k}. \tag{S63}$$

Proof of Proposition S9. The proof is similar to that of Proposition S1 from Section S1.3. We prove the stronger statement that the bounds (S62) and (S63) hold for a given $k \in [K]$ with probability $1-\delta/K$, conditional on $n_1, \ldots, n_K$. If $n_k = 0$, then there is nothing to prove, so we assume henceforth that $n_k \geq 1$. We have that

$$\big\|\overrightarrow{\mathrm{diag}}(\hat{\Gamma}_{\mathrm{w},k}) - \overrightarrow{\mathrm{diag}}(\Gamma_{\mathrm{w},k})\big\|_\infty = \bigg\|\frac{1}{n_k}\sum_{i:Y_i=k}(Z_i - \hat{\mu}_k)^{\odot 2} - \overrightarrow{\mathrm{diag}}(\Gamma_{\mathrm{w},k})\bigg\|_\infty.$$

For $j \in [d]$, denote the (j,j)th element of $\Gamma_{\mathrm{w},k}$ by $\Gamma_{\mathrm{w},k,jj}$, and define $\hat{\Gamma}_{\mathrm{w},k,jj}$ similarly. We have for $j \in [d]$ that $\sum_{i:Y_i=k}(Z_i - \hat{\mu}_k)_j^2/\Gamma_{\mathrm{w},k,jj} \mid (n_1, \ldots, n_K) \sim \chi^2_{n_k-1}/n_k$ (with the convention that the χ^2_0 distribution is a point mass at zero). By **?**, Lemma 1, for every $\delta' \in (0, 1/2]$ and $j \in [d]$, we have with conditional probability at least $1-2\delta'$ that

$$-\frac{2}{n_k}\sqrt{(n_k-1)\log(1/\delta')} \leq \frac{\hat{\Gamma}_{\mathrm{w},k,jj}}{\Gamma_{\mathrm{w},k,jj}} - \frac{n_k-1}{n_k} \leq \frac{2}{n_k}\sqrt{(n_k-1)\log(1/\delta')} + \frac{2}{n_k}\log(1/\delta').$$

Consequently, with the same conditional probability, we have

$$\big|\hat{\Gamma}_{\mathrm{w},k,jj} - \Gamma_{\mathrm{w},k,jj}\big| \leq 2\Gamma_{\mathrm{w},k,jj}\sqrt{\frac{\log(1/\delta')}{n_k}} + \frac{2\log(1/\delta')\Gamma_{\mathrm{w},k,jj}}{n_k}.$$

Setting $\delta' = \delta/(4dK)$, and taking a union bound over $j \in [d]$, it follows that (S62) holds

with conditional probability at least $1-\delta/(2K)$. Next,

$$\big\|\overrightarrow{\mathrm{diag}}(\hat{\Gamma}_{\mathrm{b},k}) - \overrightarrow{\mathrm{diag}}(\Gamma_{\mathrm{b},k})\big\|_\infty = \|(\hat{\mu}_k - \hat{\mu})^{\odot 2} - (\mu_k - \mu)^{\odot 2}\|_\infty.$$

The random variables $(\hat{\mu}_{\ell,j})_{\ell\in[K],j\in[d]}$ are independent conditonal on $(n_1,\ldots,n_K)$, with $\hat{\mu}_{\ell,j} \sim \mathcal{N}(\mu_{\ell,j}, \Gamma_{\mathrm{w},\ell,jj}/n_\ell)$. Hence, denoting by $(\hat{\mu})_j$ and $(\mu)_j$ the jth coordinates of $\hat{\mu}$ and μ respectively, writing

$$\Psi_k := \frac{(1-n_k/n)^2}{n_k}\cdot \Gamma_{\mathrm{w},k,jj} + \sum_{\ell\neq k}\frac{n_\ell}{n^2}\Gamma_{\mathrm{w},\ell,jj},$$

we have $\hat{\mu}_{k,j} - (\hat{\mu})_j \sim \mathcal{N}(\mu_{k,j}-\mu_j, \Psi_k)$. Hence $\big(\hat{\mu}_{k,j} - (\hat{\mu})_j\big)^2/\Psi_k \mid (n_1,\ldots,n_K) \sim \chi^2_1(B_{k,j})$, where $B_{k,j} := \big(\mu_{k,j} - (\mu)_j\big)^2/\Psi_k$. By **?**, Lemma 8.1, for every $\delta' \in (0,1/2]$, we have with probability at least $1-2\delta'$, that

$$\begin{aligned}-2\Psi_k\sqrt{(1+2B_{k,j})\log(1/\delta')} &\le \big(\hat{\mu}_{k,j} - (\hat{\mu})_j\big)^2 - \Psi_k(1+B_{k,j}) \\ &\le 2\Psi_k\sqrt{(1+2B_{k,j})\log(1/\delta')} + 2\Psi_k\log(1/\delta').\end{aligned}$$

Now, $\Psi_k \le R_2(1/n_k - 1/n) \le R_2/n_k$ and $\Psi_k B_{k,j} \le R_1^2$. Setting $\delta' = \delta/(4dK)$, noting that $\hat{\Gamma}_{\mathrm{b},k,jj} - \Gamma_{\mathrm{b},k,jj} = (\hat{\mu}_{k,j} - (\hat{\mu})_j)^2 - \Psi_k B_{k,j}$ and taking a union bound over $j \in [d]$, it follows that with conditional probability at least $1-\delta/(2K)$, we have

$$\begin{aligned}\big\|\overrightarrow{\mathrm{diag}}(\hat{\Gamma}_{\mathrm{b},k}) - \overrightarrow{\mathrm{diag}}(\Gamma_{\mathrm{b},k})\big\|_\infty &\le \frac{2R_2^{1/2}}{n_k^{1/2}}\sqrt{\Big(\frac{R_2}{n_k} + 2R_1^2\Big)\log\Big(\frac{4dK}{\delta}\Big)} + \frac{2R_2}{n_k}\log\Big(\frac{4dK}{\delta}\Big) \\ &\le 2R_1\sqrt{\frac{2R_2\log(4dK/\delta)}{n_k}} + \frac{4R_2\log(4dK/\delta)}{n_k},\end{aligned}$$

as required. □

S3 Auxiliary lemmas

Lemma S10. *Suppose that $K=2$ and $\mathcal{C}$ is defined as in (10). Let $(-\hat{\mu}^{(t)}, \hat{\mu}^{(t)}, I_d) \in \mathcal{C}$ be the tth iterate of the EM iteration described in (4) and (5) with data $(Z_1,Y_1),\ldots,(Z_n,Y_n)$,*

starting from $(-\hat{\mu}^{(0)}, \hat{\mu}^{(0)}, I_d)$*. Then for all* $t \geq 1$*, we have*

$$\hat{\mu}^{(t)} = \frac{1}{n}\biggl\{\sum_{i:Y_i \neq 0} (-1)^{Y_i} Z_i + \sum_{i:Y_i=0} Z_i \tanh\langle Z_i, \hat{\mu}^{(t-1)}\rangle\biggr\}.$$

Proof. At step $t \geq 1$, in the E-step, by (4), we have for $k \in \{1, 2\}$ that $L_{i,k} = \mathbb{1}_{\{Y_i = k\}}$ if $Y_i \neq 0$ and

$$L_{i,k} = \frac{e^{-\|Z_i - (-1)^k \hat{\mu}^{(t-1)}\|^2/2}}{e^{-\|Z_i - \hat{\mu}^{(t-1)}\|^2/2} + e^{-\|Z_i + \hat{\mu}^{(t-1)}\|^2/2}}$$

otherwise. In the M-step, defining

$$Q(\mu \mid \hat{\mu}^{(t-1)}) := \frac{1}{n}\sum_{i=1}^{n}(L_{i,1}\|Z_i + \mu\|^2 + L_{i,2}\|Z_i - \mu\|^2),$$

we have $\hat{\mu}^{(t)} = \operatorname{argmin}_{\mu \in \mathbb{R}^d} Q(\mu \mid \hat{\mu}^{(t-1)})$. Differentiating $Q(\mu \mid \hat{\mu}^{(t-1)})$ with respect to μ, we obtain

$$\hat{\mu}^{(t)} = \frac{1}{n}\sum_{i=1}^{n}(L_{i,2} - L_{i,1})Z_i.$$

The desired result follows since $L_{i,2} - L_{i,1} = (-1)^{Y_i}$ if $Y_i \in \{1, 2\}$, and

$$L_{i,2} - L_{i,1} = \frac{e^{-\|Z_i - \hat{\mu}^{(t-1)}\|^2/2} - e^{-\|Z_i + \hat{\mu}^{(t-1)}\|^2/2}}{e^{-\|Z_i - \hat{\mu}^{(t-1)}\|^2/2} + e^{-\|Z_i + \hat{\mu}^{(t-1)}\|^2/2}} = \frac{e^{\langle Z_i, \hat{\mu}^{(t-1)}\rangle} - e^{-\langle Z_i, \hat{\mu}^{(t-1)}\rangle}}{e^{\langle Z_i, \hat{\mu}^{(t-1)}\rangle} + e^{-\langle Z_i, \hat{\mu}^{(t-1)}\rangle}} = \tanh\langle Z_i, \hat{\mu}^{(t-1)}\rangle$$

if $Y_i = 0$. □

Lemma S11. *Let* $X_1, \ldots, X_n \overset{\text{iid}}{\sim} P$ *for some distribution* P *on* $\mathbb{R}^d$*. If* $\|\mu^*\| \leq n^{-1/4}$*, then for any Borel measurable function* $\psi : (\mathbb{R}^d)^n \to \{0, 1\}$ *of the null hypothesis* $H_0 : P = \mathcal{N}_d(0, I_d)$ *against the alternative* $H_1 : P = \frac{1}{2}\mathcal{N}_d(\mu^*, I_d) + \frac{1}{2}\mathcal{N}_d(-\mu^*, I_d)$*, we have*

$$\mathbb{P}_{H_0}\bigl(\psi(X_1, \ldots, X_n) = 1\bigr) + \mathbb{P}_{H_1}\bigl(\psi(X_1, \ldots, X_n) = 0\bigr) > 1/2.$$

Proof. Write $X = (X_1, \ldots, X_n)^\top$. Observe that, writing d_{TV} for the total variation distance

between probability measures,

$$
\begin{aligned}
\mathbb{P}_{H_0}\bigl(\psi(X)=1\bigr)+\mathbb{P}_{H_1}\bigl(\psi(X)=0\bigr) &\geq 1-d_{\mathrm{TV}}(\mathbb{P}_{H_0},\mathbb{P}_{H_1})\\
&= 1-\frac{1}{2}\int\biggl|\frac{d\mathbb{P}_{H_1}}{d\mathbb{P}_{H_0}}-1\biggr|\,d\mathbb{P}_{H_0} \geq 1-\frac{1}{2}\biggl\{\int\biggl(\frac{d\mathbb{P}_{H_1}}{d\mathbb{P}_{H_0}}-1\biggr)^2 d\mathbb{P}_{H_0}\biggr\}^{1/2}\\
&= 1-\frac{1}{2}\biggl\{\int\biggl(\frac{d\mathbb{P}_{H_1}}{d\mathbb{P}_{H_0}}\biggr)^2 d\mathbb{P}_{H_0}-1\biggr\}^{1/2}. \qquad \text{(S64)}
\end{aligned}
$$

To control the chi-squared divergence in the right-hand side of (S64) above, we let $\xi = (\xi_1,\ldots,\xi_n)^\top$ have independent Rademacher components and $W=(W_{i,j})_{i\in[n],j\in[d]}$ be a random matrix with independent $N(0,1)$ entries, independent of ξ. Then $X \overset{\mathrm{d}}{=} W$ under H_0 and $X \overset{\mathrm{d}}{=} \xi\mu^{*\top}+W$ under H_1. Let $\tilde{\xi}$ be an independent copy of ξ. Using the Ingster–Suslina device, see, e.g., **?**, **?**, Lemma 21, we have that

$$
\int\biggl(\frac{d\mathbb{P}_{H_1}}{d\mathbb{P}_{H_0}}\biggr)^2 d\mathbb{P}_{H_0} = \mathbb{E}\exp\langle\xi\mu^{*\top},\tilde{\xi}\mu^{*\top}\rangle = \cosh^n(\|\mu^*\|^2) \leq e^{n\|\mu^*\|^4/2} \leq e^{1/2},
$$

where we used the fact that $\cosh x \leq e^{x^2/2}$ for all $x\in\mathbb{R}$ in the penultimate step. The desired result follows from substituting the above bound into (S64) and the fact that $1-(e^{1/2}-1)^{1/2}/2 > 1/2$. □

We prove a generalization of Cochran's theorem for quadratic forms of independent Gaussian random vectors with a common covariance matrix, which result in independent noncentral Wishart distributions. Recall that if X is a matrix, then $\mathrm{vec}(X)$ is the vectorization of X, obtained by stacking its columns on top of each other. The Kronecker product between matrices $A=(A_{i,j})_{i\in[m],j\in[n]}$ and B is defined as

$$
A\otimes B := \begin{pmatrix} A_{1,1}B & \cdots & A_{1,n}B\\ \vdots & \ddots & \vdots\\ A_{m,1}B & \cdots & A_{m,n}B\end{pmatrix}.
$$

Recall also that when $X_1,\ldots,X_n \overset{\mathrm{iid}}{\sim} \mathcal{N}_d(0,\Sigma)$, the matrix $\sum_{i=1}^n X_iX_i^\top$ has a d-dimensional Wishart distribution with n degrees of freedom and covariance matrix $\Sigma\in\mathbb{S}_+^{d\times d}$, denoted $\mathcal{W}_d(n,\Sigma)$. More generally, $\sum_{i=1}^n(X_i+\mu_i)(X_i+\mu_i)^\top$ has a non-central Wishart distribution with n degrees of freedom, covariance matrix Σ and non-centrality matrix $\Omega=\sum_{i=1}^n\mu_i\mu_i^\top$,

written $\mathcal{W}_d(n, \Sigma; \Omega)$. Thus $\mathcal{W}_d(n, \Sigma; 0) \overset{d}{=} \mathcal{W}_d(n, \Sigma)$.

Lemma S12. *Let $Z_1, \ldots, Z_n$ be independent with $Z_i \sim \mathcal{N}_d(\mu_i, \Sigma)$ for $i \in [n]$, and write $Z := (Z_1, \ldots, Z_n)^\top \in \mathbb{R}^{n \times d}$ and $M := \mathbb{E}(Z)$. If $P_1, \ldots, P_k \in \mathbb{R}^{n \times n}$ are positive semidefinite matrices such that $P_1 + \cdots + P_k = I_n$ and $\mathrm{rank}(P_1) + \cdots + \mathrm{rank}(P_k) = n$, then $Z^\top P_1 Z, \ldots, Z^\top P_k Z$ are independent with $Z^\top P_r Z \sim \mathcal{W}_d\bigl(\mathrm{rank}(P_r), \Sigma; M^\top P_r M\bigr)$.*

Proof. As in the proof of the classical Cochran's theorem (**?**), we first note that $P_1, \ldots, P_k$ can be simultaneously diagonalized such that

$$P_r = Q D_r Q^\top,$$

for some $Q \in \mathbb{O}^{n \times n}$ and $D_r = \mathrm{diag}\bigl((\mathbb{1}_{\{j \in S_r\}})_{j \in [n]}\bigr)$, where $S_r \subseteq [n]$, $|S_r| = \mathrm{rank}(P_r)$ and $S_r \cap S_{r'} = \emptyset$ for all $r \neq r'$. In particular, $P_1, \ldots, P_k$ satisfy $P_r^2 = P_r$ for $r \in [k]$ and $P_r P_{r'} = 0$ for all $r \neq r'$. Since $P_1 Z, \ldots, P_k Z$ are jointly Gaussian, with

$$\begin{aligned} \mathrm{Cov}\bigl(\mathrm{vec}(P_r Z), \mathrm{vec}(P_{r'} Z)\bigr) &= \mathrm{Cov}\bigl((I_d \otimes P_r)\mathrm{vec}(Z), (I_d \otimes P_{r'})\mathrm{vec}(Z)\bigr) \\ &= (I_d \otimes P_r)(\Sigma \otimes I_n)(I_d \otimes P_{r'})^\top = 0, \end{aligned}$$

we have that $P_1 Z, \ldots, P_k Z$ are independent. But $Z^\top P_r Z = (P_r Z)^\top P_r Z$, so it follows that $Z^\top P_1 Z, \ldots, Z^\top P_k Z$ are independent. Moreover, writing $W = (W_1, \ldots, W_n)^\top := Q^\top Z$, we have $\mathrm{vec}(Z) \sim \mathcal{N}_{nd}\bigl(\mathrm{vec}(M), \Sigma \otimes I_n\bigr)$, so

$$\mathrm{vec}(W) = (I_d \otimes Q^\top)\mathrm{vec}(Z) \sim \mathcal{N}_{nd}\bigl((I_d \otimes Q^\top)\mathrm{vec}(M), \Sigma \otimes I_n\bigr) \overset{d}{=} \mathcal{N}_{nd}\bigl(\mathrm{vec}(Q^\top M), \Sigma \otimes I_n\bigr).$$

Therefore,

$$\begin{aligned} Z^\top P_r Z = W^\top D_r W = \sum_{i \in S_r} W_i W_i^\top &\sim \mathcal{W}_d\biggl(|S_r|, \Sigma; \sum_{i \in S_r} \mathbb{E}(W_i)\mathbb{E}(W_i)^\top\biggr) \\ &\overset{d}{=} \mathcal{W}_d\bigl(\mathrm{rank}(P_r), \Sigma; M^\top P_r M\bigr), \end{aligned}$$

as desired. □

Lemma S13. *For any $a, b \in \mathbb{R}$, we have*

$$\frac{1}{2}\{\tanh(a + b) - \tanh(a - b)\} \leq |b|$$

and

$$\frac{a}{2}\{\tanh(a+b)+\tanh(a-b)\} \geq a^2 - \frac{a^4}{3} - a^2b^2$$

Proof. For the first inequality, since the left-hand side is an increasing function of b, and an even function of a, we may assume that $a \geq 0$ and $b \geq 0$. Notice that $\frac{\partial}{\partial a}\big(\tanh(a+b) - \tanh(a-b)\big) = 1/\cosh^2(a+b) - 1/\cosh^2(|a-b|) \leq 0$, since $x \mapsto \cosh(x)$ is an increasing function on $[0,\infty)$. Hence

$$\frac{1}{2}\{\tanh(a+b)-\tanh(a-b)\} \leq \tanh b \leq b.$$

as desired.

For the second inequality, since both sides are even functions of both a and b, we may again assume without loss of generality that $a > 0$ and $b \geq 0$. We may also assume that $b \leq 1$ since otherwise, the right-hand side is negative and the inequality holds trivially. But then

$$\begin{aligned}\frac{1}{2}\{\tanh(a+b)+\tanh(a-b)\} &= \frac{1}{2}\left(\frac{\tanh a+\tanh b}{1+\tanh a\tanh b} + \frac{\tanh a-\tanh b}{1-\tanh a\tanh b}\right)\\ &= \frac{\tanh a}{(1-\tanh^2 a\tanh^2 b)\cosh^2 b} \geq \frac{\tanh a}{\cosh^2 b}\\ &\geq (1-b^2)\tanh a \geq (1-b^2)\left(a - \frac{a^3}{3}\right) \geq a - \frac{a^3}{3} - ab^2,\end{aligned}$$

as desired. Here, the second inequality holds because $(1-b^2)\cosh^2 b \leq (1-b^2)e^{b^2} \leq 1$. □

Lemma S14. *Let $H : [0,\infty) \to [0,\infty)$ be an increasing, concave function with $H'(x_0) < 1$ for some $x_0 \geq 0$ and either $H(0) > 0$ or both $H(0) = 0$ and $H'(0) > 1$. Then there exists a unique $\alpha^* > 0$ such that*

$$H(\alpha) - \alpha \begin{cases} > 0 & \alpha \in (0,\alpha^*) \\ = 0 & \alpha = \alpha^* \\ < 0 & \alpha \in (\alpha^*,\infty). \end{cases}$$

Moreover, if $\alpha_0 > 0$, then the sequence $(\alpha_t)_{t\geq 0}$ given by $\alpha_t := H(\alpha_{t-1})$ monotonically converges to α^*.

Proof. For the first claim, consider the concave function $\tilde{H}(x) := H(x) - x$, which satisfies

$\tilde{H}(x) > 0$ for sufficiently small $x > 0$, and for $x \geq x_0$, we have that any supergradient $v_x \in \mathbb{R}$ of $\tilde{H}$ at x satisfies $v_x \leq -\{1 - H'(x_0)\} < 0$. It follows that $\tilde{H}(x) \to -\infty$ as $x \to \infty$, so by the intermediate value theorem, there exists $\alpha^* \in (0, \infty)$ such that $\tilde{H}(\alpha^*) = 0$, i.e. $H(\alpha^*) = \alpha^*$. Again using the facts that $\tilde{H}(x) > 0$ for sufficiently small $x > 0$, and $\tilde{H}(x) \to -\infty$ as $x \to \infty$, we see that the concave function $\tilde{H}$ can only cross the x-axis at one positive value α^*, and $\tilde{H}(\alpha) > 0$ for $\alpha \in (0, \alpha^*)$ and $\tilde{H}(\alpha) < 0$ for $\alpha \in (\alpha^*, \infty)$.

Next, note that if $\alpha \in (0, \alpha^*)$, then $\alpha < H(\alpha) < H(\alpha^*) = \alpha^*$. Thus, if $\alpha_0 < \alpha^*$, then $(\alpha_t)_{t \geq 0}$ is an increasing sequence, bounded above by α^*, so it converges to a limit. But then, taking limits on both sides of the recursion $\alpha_t := H(\alpha_{t-1})$, we deduce that this limit must be α^*. A similar argument can be used to show that if $\alpha_0 \in (\alpha^*, \infty)$ then $(\alpha_t)_{t \geq 0}$ decreases down to the limit α^*, while if $\alpha_0 = \alpha^*$, then $\alpha_t = \alpha^*$ for all t. □

Lemma S15. *Let μ^* be a non-zero vector in $\mathbb{R}^d$, let $Z \sim \frac{1}{2}\mathcal{N}_d(-\mu^*, I_d) + \frac{1}{2}\mathcal{N}_d(\mu^*, I_d)$, let $\eta := \mu^*/\|\mu^*\|$, and define $q : [0, \infty) \to [0, \infty)$ by*

$$q(\alpha) := \begin{cases} \alpha^{-1}\eta^\top \mathbb{E}(Z \tanh\langle \alpha\eta, Z\rangle) & \text{if } \alpha > 0 \\ 1 + \|\mu^*\|^2 & \text{if } \alpha = 0. \end{cases}$$

Then q is a differentiable function with $q(\|\mu^*\|) = 1$ and for any $h \geq \|\mu^*\|$, we have

$$\sup_{\alpha \in [0,h]} \frac{q'(\alpha)}{\alpha} \leq -\frac{e^{-h^2/2}}{3 \cdot 2^{11}\sqrt{2\pi}(h^5 \vee 1)}.$$

Proof. Write $s := \|\mu^*\|$. The fact that $q(s) = 1$ follows from **?**, Theorem 1. By Wu and Zhou (2022, Lemma 3(4)), q is differentiable with $q'(\alpha) \leq -(2\alpha/3) \cdot \mathbb{E}\big(\tilde{Z}^4/\cosh^2(\alpha\tilde{Z})\big)$ for

$\alpha \in [0, \infty)$, where $\tilde{Z} \sim \frac{1}{2}\mathcal{N}(-s, 1) + \frac{1}{2}\mathcal{N}(s, 1)$. We can now compute that for $\alpha, s \in [0, h]$,

$$\begin{aligned}
\mathbb{E}\biggl(\frac{\tilde{Z}^4}{\cosh^2(\alpha\tilde{Z})}\biggr) &\geq \mathbb{E}(\tilde{Z}^4 e^{-2\alpha|\tilde{Z}|}) \geq \frac{1}{2\sqrt{2\pi}} \int_0^\infty y^4 e^{-2\alpha y} e^{-(y-s)^2/2}\, dy \\
&= \frac{1}{2\sqrt{2\pi}} \int_0^\infty y^4 e^{-(y-s+2\alpha)^2/2 - 2\alpha s + 2\alpha^2}\, dy \\
&\geq \begin{cases} \frac{e^{-2\alpha s+2\alpha^2}}{2\sqrt{2\pi}} \int_0^{\frac{1}{2(2\alpha-s)}} \frac{1}{2} y^4 e^{-(2\alpha-s)^2/2}\, dy & \text{if } s+1 \leq 2\alpha \\ \frac{e^{-2\alpha s+2\alpha^2}}{2\sqrt{2\pi}} \int_{s-2\alpha+1}^{s-2\alpha+2} y^4 e^{-2}\, dy & \text{if } s+1 > 2\alpha \end{cases} \\
&\geq \begin{cases} \frac{e^{-s^2/2}}{2^7 \cdot 5\sqrt{2\pi}} \Bigl(\frac{1}{2\alpha-s}\Bigr)^5 & \text{if } s+1 \leq 2\alpha \\ \frac{e^{-2\alpha s+2\alpha^2-2}}{10\sqrt{2\pi}} & \text{if } s+1 > 2\alpha \end{cases} \\
&\geq \frac{e^{-h^2/2}}{2^{12} \cdot 5\sqrt{2\pi}(h^5 \vee 1)},
\end{aligned}$$

which establishes the desired bound. □

Lemma S16. *Let $d \geq 2$, and let $\eta = (\eta_1, \ldots, \eta_d)^\top \sim \mathrm{Unif}(\mathcal{S}^{d-1})$. Then for any $a > 0$, we have*

$$\mathbb{P}\biggl(|\eta_1| \leq \frac{a}{\sqrt{d}}\biggr) \leq \sqrt{\frac{2}{\pi}}a.$$

Proof. Leting $Z = (Z_1, \ldots, Z_d)^\top \sim N_d(0, I_d)$, we have $\eta \overset{\mathrm{d}}{=} Z/\|Z\|$ and in particular $\eta_1^2 \overset{\mathrm{d}}{=} Z_1^2/(Z_1^2 + \cdots + Z_d^2) \sim \mathrm{Beta}\bigl(1/2, (d-1)/2\bigr)$. Thus,

$$\begin{aligned}
\mathbb{P}\biggl(|\eta_1| \leq \frac{a}{\sqrt{d}}\biggr) = \mathbb{P}\biggl(\frac{Z_1^2}{\|Z\|^2} \leq \frac{a^2}{d}\biggr) &= \frac{\Gamma(d/2)}{\Gamma(1/2)\Gamma\bigl((d-1)/2\bigr)} \int_0^{a^2/d} t^{-1/2}(1-t)^{(d-3)/2}\, dt \\
&\leq \frac{2\Gamma(d/2)a}{\sqrt{d}\Gamma(1/2)\Gamma((d-1)/2)} \leq \sqrt{\frac{2}{\pi}}a,
\end{aligned}$$

where the final bound uses, e.g., **?**, Corollary 11. □

S4 Additional simulation results

S4.1 Choice of tuning parameters

The purpose of this subsection is to investigate the effect of the various input parameters A, B, d and ℓ in Algorithm 1, and to recommend sensible default choices. In Figure S1, we plot the misclustering rate with Algorithm 3 as a base procedure in our Gaussian semi-supervised learning setting as each of these parameters varies, for four different SNR levels. After applying Algorithm 1, we obtain our final estimated cluster labels by using Algorithm 3 again on the data projected onto the selected coordinates with a single hierarchical clustering initialization. We then output the predicted labels, computed as $\hat{y}_i := \mathrm{sargmax}_{k\in[K]} L_{i,k}$ for $i \in [n]$, instead of $\hat{Q}$.

The panels in Figure S1 reveal that the misclustering rate is quite robust to the choices of A, B and d, and that it is less serious (and may even help) to choose ℓ larger—rather than smaller—than s_0. Our intuition regarding these parameters is as follows: the effect of the choice of A is relatively straightforward in that, subject to the fact that the computational burden increases with A, one would always like to aggregate over a larger number of good random projections than a smaller one. This is quantified theoretically by the bound in Theorem 2. One can also see this effect in the bottom-left panel of Figure S1, which indicates that $A = 150$ suffices for almost optimal performance (though there appears to be some penalty for choosing it to be as small as 50). On the other hand, the effect of B is a little more complicated: the hypothetical choice $B = \infty$ would lead to us selecting the same random projection in each group, and there would be no benefit to choosing A to be larger than 1. This does not appear to be ideal in practice, and our empirical experience is that some diversity in the A selected projections allows greater benefit from the effect of aggregation. Based on the bottom-right panel of Figure S1, we recommend $B = 75$ as a default choice.

There is no clear trend on performance with the choice of d, so for simplicity we took $d = s_0$ in our remaining simulations below. Finally, the misclustering rate appears to decrease as ℓ increases, with an elbow in the curve visible at the highest value of the SNR when ℓ is set to the true sparsity level s_0. Of course, if ℓ is chosen to be very large, then we will include many noise variables, and the misclustering rate will eventually deteriorate. Nevertheless, the bottom-right panel of Figure S1 indicates that the gain in increasing the probability of including all signal variables may outweigh the penalty of also including more noise variables—as expected, this effect is larger when the SNR is larger. For simplicity we choose $\ell = s_0$ in our remaining simulations, though we recommend practitioners err on the

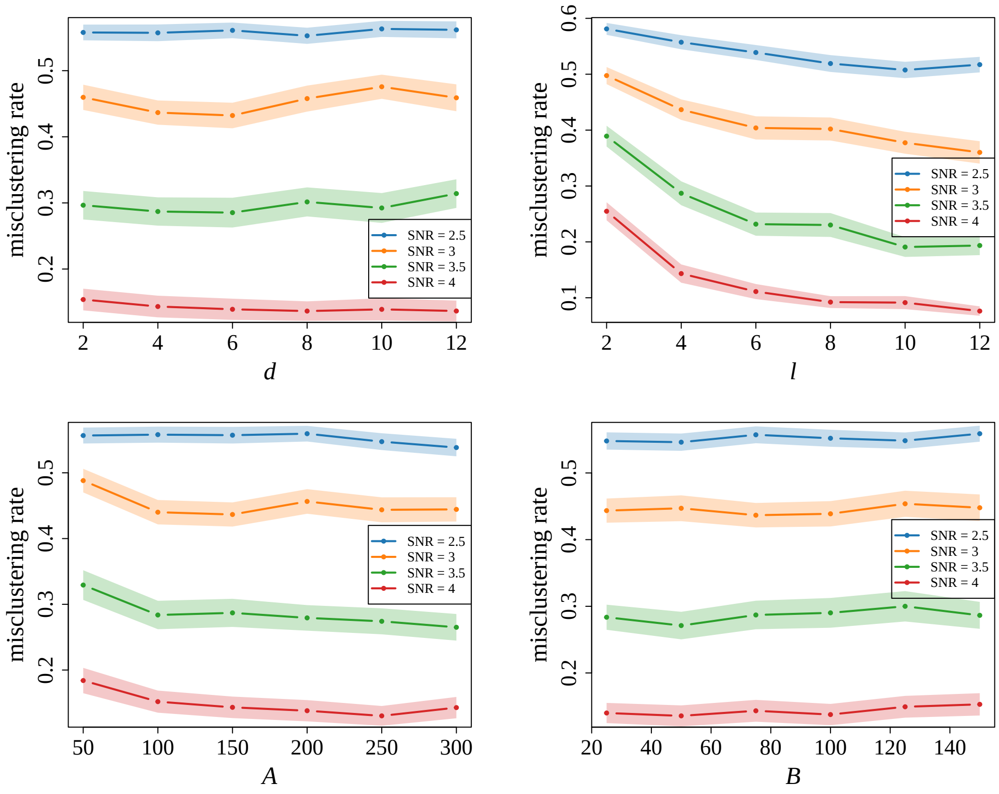

Figure S1: Average misclustering rate over 200 repetitions in our anisotropic Gaussian semi-supervised learning problem with $n = 250$, $p = 600$, $s = 4$, $K = 3$, $\gamma = 0.05$, $\mathrm{SNR} \in \{2.5, 3, 3.5, 4\}$ and $\Sigma_{\mathrm{w}} = V\Lambda V^{\top}$, where $\Lambda \in \mathbb{R}^{p\times p}$ is diagonal V is independent of Λ, and generated according to the Haar measure on $\mathbb{O}^{p\times p}$. For each of the four panels, we fix three of $d = 4$, $\ell = 4$, $A = 150$, $B = 75$, and vary the remaining one. The shaded regions represent interpolated 95% confidence intervals at each of the points.

side of choosing larger ℓ.

S4.2 Model misspecification

In this subsection we present some additional simulation results where the modeling assumptions in Section 3.3 are violated. We consider two different types of departure from our basic model: in the first, we study a setting where the discriminant directions between the different classes are not particularly sparse; in the second, we allow the class-conditional distributions to have tails that are heavier than Gaussian. To be more precise, in Figure S2, we set $p \in \{200, 600\}$, and chose $K = 3$ cluster means where the magnitudes of their signal coordinates decrease in a harmonic fashion, with $s = 120$ signal coordinates in total, so that

$$\begin{aligned}
\mu_1 &= a(1, 1/2, 0, 1/4, 1/5, 0, \ldots, 1/118, 1/119, 0, \mathbf{0}_{p-120})^\top, \\
\mu_2 &= a(-1, 0, 1/3, -1/4, 0, 1/6, \ldots, -1/118, 0, 1/120, \mathbf{0}_{p-120})^\top, \\
\mu_3 &= a(0, -1/2, -1/3, 0, -1/5, -1/6, \ldots, 0, -1/119, -1/120, \mathbf{0}_{p-120}),
\end{aligned}$$

where $a > 0$ is chosen to ensure that the minimal pairwise distance between the clusters is equal to the given SNR. In other respects, the set-up is the same as that for Figure 1. The `Sharp-SSL+EM` algorithm continues to perform very well in this misspecified setting, though we remark that the `SPCA+Kmeans` algorithm is also very competitive here.

In Figure S3, we again consider a similar set-up to that in Figure 1, but now choose the class-conditional distributions to have either independent t_6 components or independent Laplace components. The performance of the `Sharp-SSL+EM` algorithm is again robust to these departures from our modeling assumptions, though we note that in these examples, the method of Witten and Tibshirani (2010) also performs very well. These additional simulations provide reassurance that the `Sharp-SSL+EM` algorithm is robust to these types of model misspecification.

S4.3 Initialization

As mentioned in Section 4, when applying the `Sharp-SSL` algorithm using EM as the base procedure, we initialize the EM algorithm with the output of hierarchical clustering labels as implemented in the `mclust` `R` package. Since hierarchical clustering requires computation of pairwise distances between $X_1, \ldots, X_n$, the initialization step has a time complexity of $O(dn^2)$ and so can be more costly than the actual EM iterations for large n. However, as

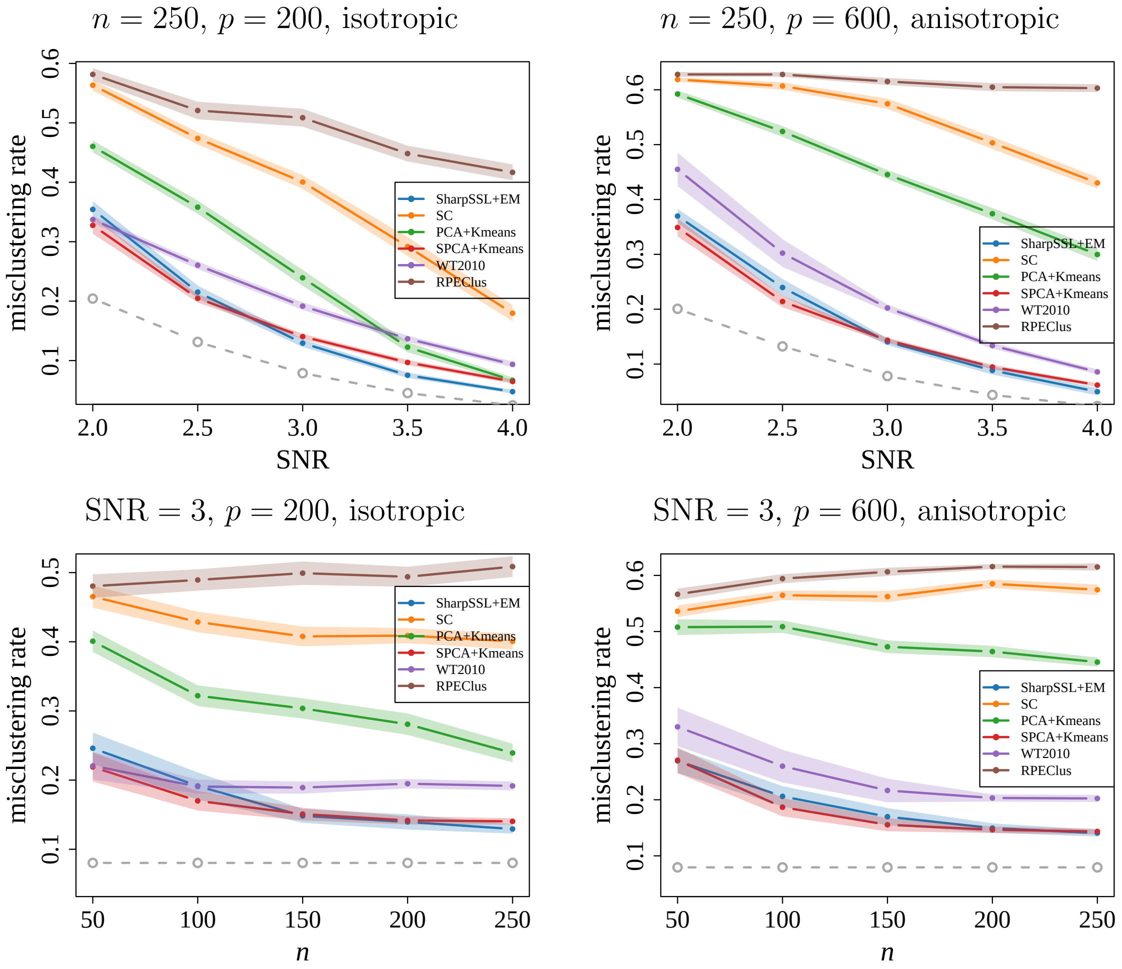

Figure S2: Average misclustering rate over 100 repetitions using `Sharp-SSL+EM` followed by the EM algorithm, as well as using the other methods from Section 4.2. Data are generated from the normal mixture distribution described at the beginning of Section 4 with $K = 3$ and $p = 200$ (left) as well as $p = 600$ (right). The three cluster means are chosen as described in Section S4.2. For isotropic settings (left), $\Sigma_{\mathrm{w}} = I_p$; for anisotropic settings (right), $\Sigma_{\mathrm{w}} = V \Lambda V^\top$, where $\Lambda \in \mathbb{R}^{p\times p}$ is diagonal with independent $\mathrm{Unif}[0, 2]$ diagonal entries and V is independent of Λ, and sampled from the Haar measure on $\mathbb{O}^{p\times p}$. In the top panels, $n = 250$ and the SNR varies; in the bottom panels, $\mathrm{SNR} = 3$ and n varies. The shaded regions represent interpolated 95% confidence intervals at each of the points.

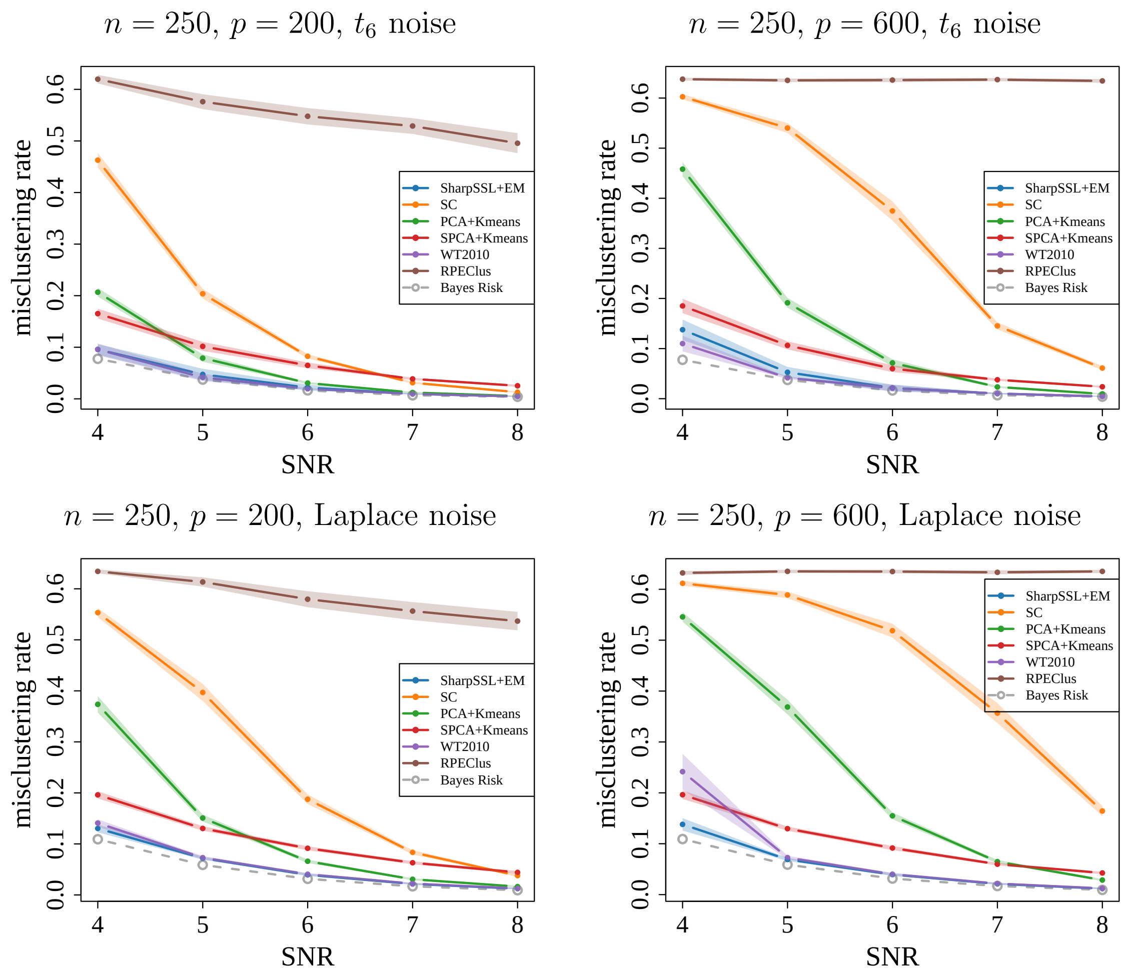

Figure S3: Average misclustering rate over 100 repetitions using `Sharp-SSL+EM` followed by the EM algorithm, as well as using the other methods from Section 4.2. Data are generated as in Figure 2 of the main text, except that the Gaussian class-conditional distributions are replaced with independent t_6 components (top) or independent Laplace components (bottom). The shaded regions represent interpolated 95% confidence intervals at each of the points.

suggested by the theoretical results in Section 3.3, as the sample size increases, we would expect the sample EM iterations to closely follow the population EM iterations (cf. (S17)), which converge to population parameters under mild conditions (Balakrishnan et al., 2017, Corollary 1). This suggests that initialization is less important for larger sample sizes. This theoretical intuition is confirmed empirically by Figure S4. Here, we study four different initializing cluster labels for the EM algorithm: (i) random initialization where labels are drawn from a discrete uniform distribution on $[K]$; (ii) the hierarchical clustering initializer as implemented in the `mclust R` package; (iii) the cluster labels assigned by Lloyd's algorithm for k-means clustering, which is itself randomly initialized with K data points as starting cluster centroids; and (iv) the cluster labels assigned by the k-means++ algorithm of **?**. The left panel of Figure S4 reports, for various n and SNR, the maximum Frobenius norm difference among the estimated cluster centroids using the EM algorithm with each of the four different initializers. We see that for SNR above 1.5, the outputs from all four initializers are very close when $n \geq 800$. The right panel plots the excess misclustering error rate of initializers (ii), (iii) and (iv) over initializer (i) for the same choices of n and SNR. We see that for all SNR and when n is large, the random initialization has a very similar misclustering rate to the remaining initializing methods, if not slightly better. Motivated by this, in our implementation, we suggest using initialization with hierarchical clustering labels when $n \leq 1000$, and uniformly random initialization of labels when $n > 1000$.

S4.4 Timing comparison

An advantage of working with axis-aligned projections is that they are extremely cheap to compute: we just have to select a d-dimensional subvector of a p-dimensional vector, which takes $O(p + d \log d)$ operations (by contrast, generating a non-axis-aligned projection requires $O(d^2 p)$ operations). We also mention that the grouping structure of the random projections makes it very convenient to parallelise the `Sharp-SSL` procedure, where we can generate each group of random projections on a different core.

As an empirical comparison, we studied the running times of several different alternative algorithms across the settings used in Figure 1. For the methods that involve random projections, i.e. `Sharp-SSL+EM`, `SPCA+Kmeans`, `SPCA+EM` and `RPEClus`, which are trivially parallelizable, we used 50 nodes. The results are presented in Table S1. We see that while the `Sharp-SSL+EM` algorithm is not the fastest available method, it is by no means slow, and can be applied on high-dimensional problem instances without difficulty; the very substantial gain in statistical performance more than compensates for the running time.

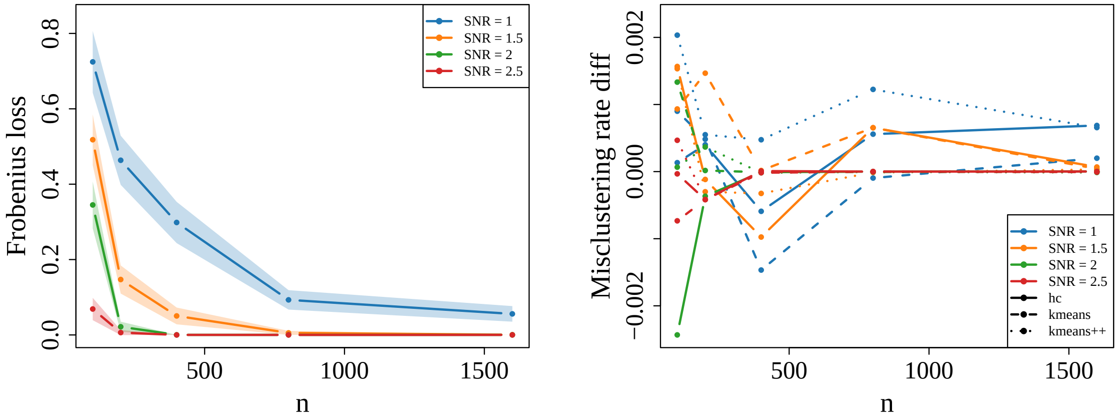

Figure S4: Comparison of parameter estimators and misclustering rates of the `Sharp-SSL+EM` using the four different initialization methods mentioned in Section S4.3. Data are generated from the normal mixture distribution as in Figure 1 with $K = 3$ and $p = 3$, $\gamma = 0$, SNR $\in \{1, 1.5, 2, 2.5\}$ and $n \in \{100, 200, 400, 800, 1600\}$. The left panel displays the maximum Frobenius distance between estimated cluster centroids of the EM algorithm with the four different initialization methods, averaged over 1000 Monte Carlo repetitions. The right panel shows the excess misclustering rate from using hierarchical clustering, k-means and k-means++ initializers, in comparison with the random initializer, where again the average value is computed over 1000 Monte Carlo repetitions.

Table S1: Average running times in seconds per instance of various algorithms in settings of Figure 1.

SharpSSL+EM	SC	KM	PCA+KMeans	PCA+EM	SPCA+KMeans	SPCA+EM	WT10	RPEClus
1.05	0.049	0.043	0.025	0.045	0.97	1.03	3.17	6.23

Our empirical observation is that the main SharpSSL+EM algorithm scales approximately linearly in n, though as mentioned in Section S4.3 the hierarchical clustering initialization scales as $O(n^2)$. Using random cluster label initialization, with labels drawn from a discrete uniform distribution on $[K]$ for $n > 1000$ as suggested at the end of Section S4.3, we observed a running time of 13.76 seconds for the SharpSSL+EM algorithm with $n = 128000$ on a 50-node cluster, and 18 minutes on a 4-core laptop machine with a 3.2 GHz CPU. This confirms the SharpSSL+EM algorithm could be applied in genomics applications, for example, where such sample sizes are frequently encountered.
