# Supplementary material for: Sharp-SSL: Selective High-Dimensional Axis-Aligned Random Projections for Semi-Supervised Learning
Source: J Am Stat Assoc. 2024 Apr 12;120(549):395–407. doi: 10.1080/01621459.2024.2340792 (PMC12012707; doi:10.1080/01621459.2024.2340792)
Supplement: acc-form-2021.pdf [file UASA_A_2340792_SM6404.pdf]

Author Contributions Checklist Form

This form documents the artifacts associated with the article (i.e., the data and code supporting the computational findings) and describes how to reproduce the findings.

Part 1: Data

☐ This paper **does not** involve analysis of external data (i.e., no data are used or the only data are generated by the authors via simulation in their code).

☒ I certify that the author(s) of the manuscript have legitimate access to and permission to use the data used in this manuscript.

Abstract

We used a gene expression dataset available in the ‘datamicroarray’ R package.

Availability

☒ Data **are** publicly available
☐ Data **cannot be made** publicly available

If the data are publicly available, see the *Publicly available data* section. Otherwise, see the *Non-publicly available data* section, below.

Publicly available data

☒ Data are available online at:
https://github.com/ramhiser/datamicroarray/blob/master/data/alon.RData
☒ Data are available as part of the paper’s supplementary material.
☐ Data are publicly available by request, following the process described here:

☐ Data are or will be made available through some other mechanism, described here:

Non-publicly available data

Discussion of lack of publicly available data:

Description

File format(s)

☐ CSV or other plain text:
☒ Software-specific binary format (.Rda, Python pickle, etc.): .RData
☐ Standardized binary format (e.g., netCDF, HDF5, etc.):
☐ Other (described here):

Data dictionary

☒ Provided by the authors in the following file(s): data_description.txt
☐ Data file(s) is (are) self-describiing (e.g., netCDF files)
☐ Available at the following URL:

Click or tap here to enter text.

Additional information (optional)

Part 2: Code

Abstract

Code for the algorithm is contained in algorithms.R, with helper functions defined in ancillary.R.
Code for generating all figures in the paper are contained in fig1_simulation.R, fig1_plot.R etc.

Description

Code format(s)

☒ Script files
- ☒ R ☐ Python ☐ Matlab
- ☐ Other:

☐ Package
- ☐ R ☐ Python ☐ MATLAB toolbox
- ☐ Other:

☐ Reproducible report
- ☐ R Markdown ☐ Jupyter notebook
- ☐ Other:

☐ Shell script

☐ Other (described here):

Supporting software requirements

Version of primary software used

R4.1.1

Libraries and dependencies used by the code

R packages from CRAN: clue v0.3-65, mclust v6.0.1, SPCAvRP v0.4
R package installable from GitHub: putils v1.3.2

Supporting system/hardware requirements (optional)

Code is run in parallel in a 100 machines slurm cluster.

Parallelization used

☐ No parallel code used
☐ Multi-core parallelization on a single machine/node
Number of cores used:
☒ Multi-machine/multi-node parallelization
Number of nodes and cores used: 100

License

☒ MIT License (default)
☐ BSD
☐ GPL v3.0
☐ Creative Commons
☐ Other (described here):

Additional information (optional)

Part 3: Reproducibility workflow

Scope

The provided workflow reproduces:
☐ Any numbers provided in text in the paper
☒ The computational method(s) presented in the paper (i.e., code is provided that implements the method(s))
☒ All tables and figures in the paper
☐ Selected tables and figures in the paper, as explained and justified here:

Workflow details

Format(s)

☐ Single master code file
☐ Wrapper (shell) script(s)
☐ Self-contained R Markdown file, Jupyter notebook, or other literate programming approach
☒ Text file (e.g., a readme-style file) that documents workflow
☐ Makefile
☐ Other (more detail in 'Instructions' below)

Instructions

Workflow detailed in readme.md

Expected run-time

Approximate time needed to reproduce the analyses on a standard desktop machine:
☐ <1 minute
☐ 1-10 minutes
☐ 10-60 minutes

☐ 1-8 hours
☒ >8 hours
☒ Not feasible to run on a desktop machine, as described here:

While the code can be run on a desktop machine for a small number of repetitions, to fully generate the figures with the required number, simulations should be carried out in a cluster in parallel.

Additional documentation (optional)

Notes (optional)
