# Supplementary material for: Sharp-SSL: Selective High-Dimensional Axis-Aligned Random Projections for Semi-Supervised Learning
Source: J Am Stat Assoc. 2024 Apr 12;120(549):395–407. doi: 10.1080/01621459.2024.2340792 (PMC12012707; doi:10.1080/01621459.2024.2340792)
Supplement: suppl_data__2_.zip [file UASA_A_2340792_SM3155.zip › suppl_data.zip!/suppl_data/figures/fig3a.pdf]

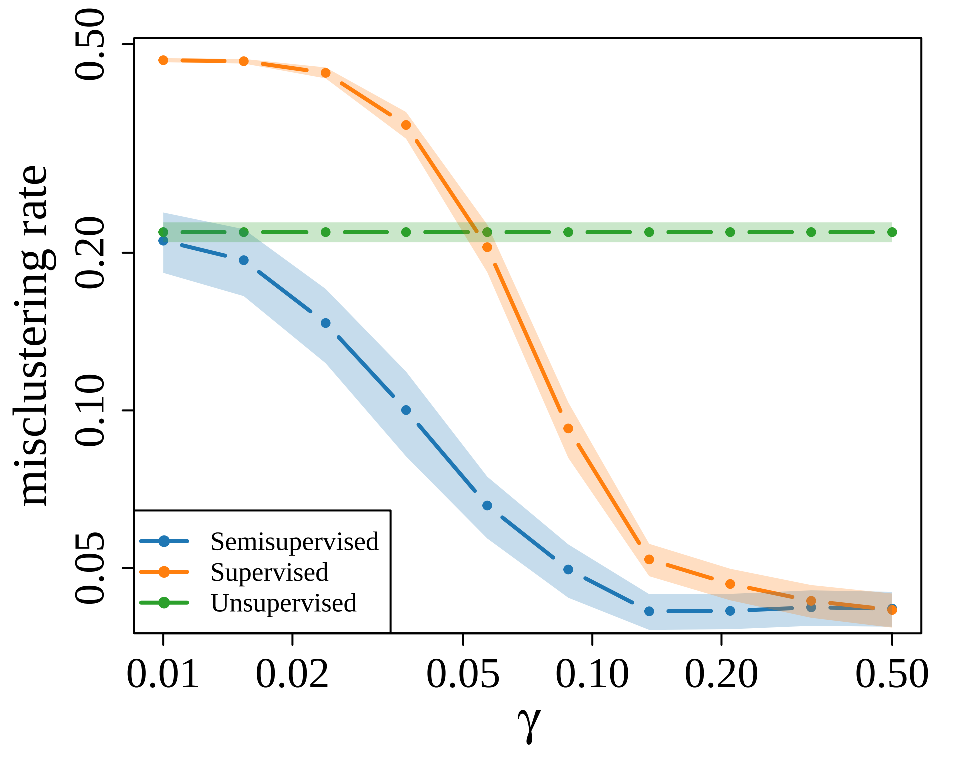

misclustering rate
0.50
0.20
0.10
0.05
0.01
0.02
0.05
0.10
0.20
0.50
γ
Semisupervised
Supervised
Unsupervised
