# Supplementary material for: Sharp-SSL: Selective High-Dimensional Axis-Aligned Random Projections for Semi-Supervised Learning
Source: J Am Stat Assoc. 2024 Apr 12;120(549):395–407. doi: 10.1080/01621459.2024.2340792 (PMC12012707; doi:10.1080/01621459.2024.2340792)
Supplement: suppl_data__2_.zip [file UASA_A_2340792_SM3155.zip › suppl_data.zip!/suppl_data/figures/figS4a.pdf]

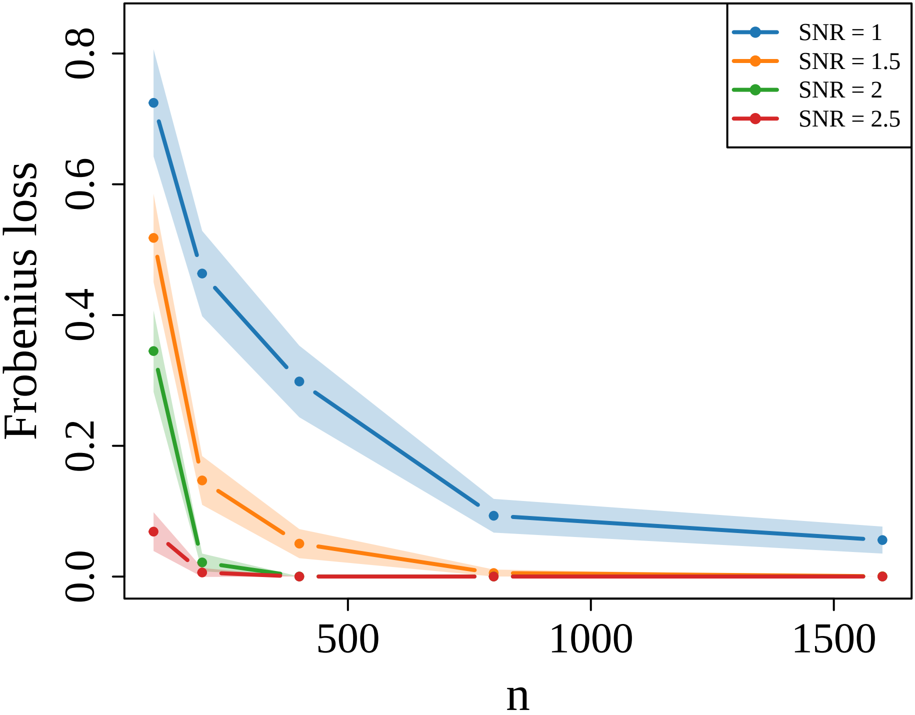

SNR = 1
SNR = 1.5
SNR = 2
SNR = 2.5
Frobenius loss
0.0
0.2
0.4
0.6
0.8
500
1000
1500
n
